# Supplementary material for: The use of a speaking book® to enhance vaccine knowledge among caregivers in The Gambia: A study using qualitative and quantitative methods
Source: BMJ Open. 2021 Mar 8;11(3):e040507. doi: 10.1136/bmjopen-2020-040507 (PMC7942236; doi:10.1136/bmjopen-2020-040507)
Supplement: Supplementary data [file bmjopen-2020-040507supp001.pdf]

Supplementary material 1_Photos of the Speaking Book

Photos of the speaking book

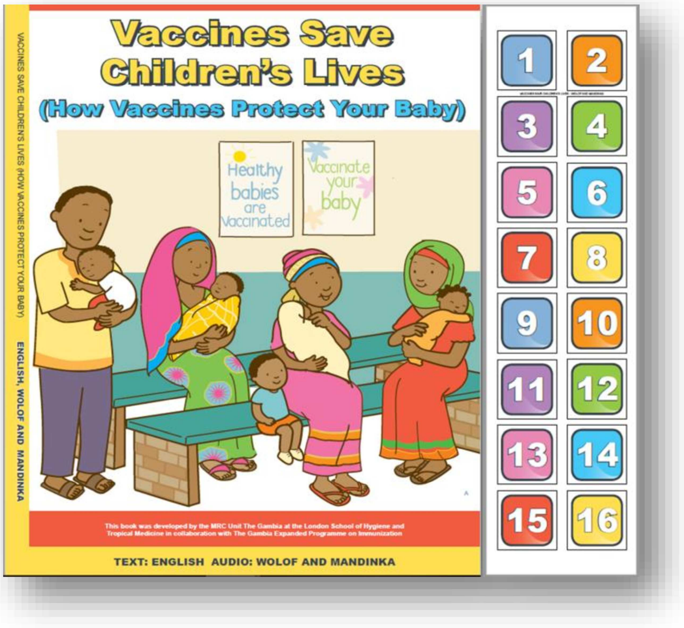

Front view of the SB

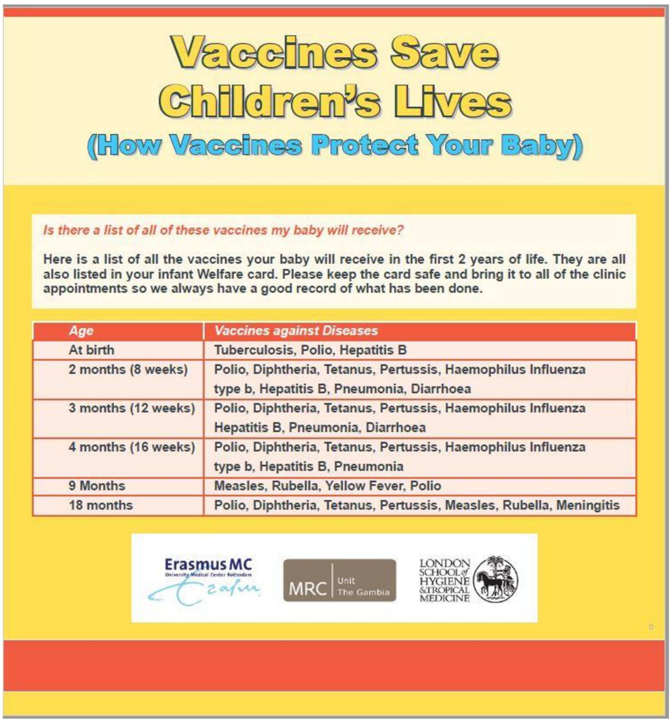

Vaccines Save Children's Lives
(How Vaccines Protect Your Baby)

Is there a list of all of these vaccines my baby will receive?

Here is a list of all the vaccines your baby will receive in the first 2 years of life. They are all also listed in your infant Welfare card. Please keep the card safe and bring it to all of the clinic appointments so we always have a good record of what has been done.

Age	*Vaccines against Diseases*
At birth	Tuberculosis, Polio, Hepatitis B
2 months (8 weeks)	Polio, Diphtheria, Tetanus, Pertussis, Haemophilus Influenza type b, Hepatitis B, Pneumonia, Diarrhoea
3 months (12 weeks)	Polio, Diphtheria, Tetanus, Pertussis, Haemophilus Influenza Hepatitis B, Pneumonia, Diarrhoea
4 months (16 weeks)	Polio, Diphtheria, Tetanus, Pertussis, Haemophilus Influenza type b, Hepatitis B, Pneumonia
9 Months	Measles, Rubella, Yellow Fever, Polio
18 months	Polio, Diphtheria, Tetanus, Pertussis, Measles, Rubella, Meningitis

Erasmus MC University Medical Center Rotterdam
MRC Unit The Gambia
LONDON SCHOOL of HYGIENE &TROPICAL MEDICINE

Back view of the SB

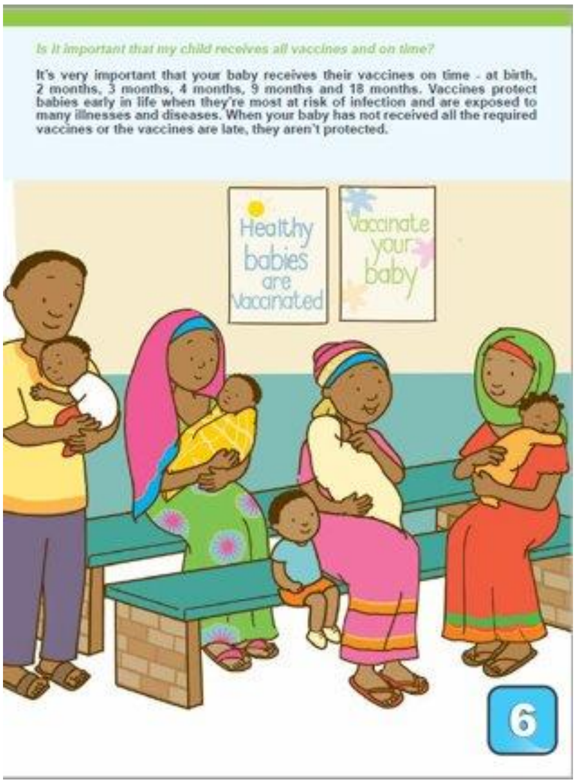

Page 6 of the SB

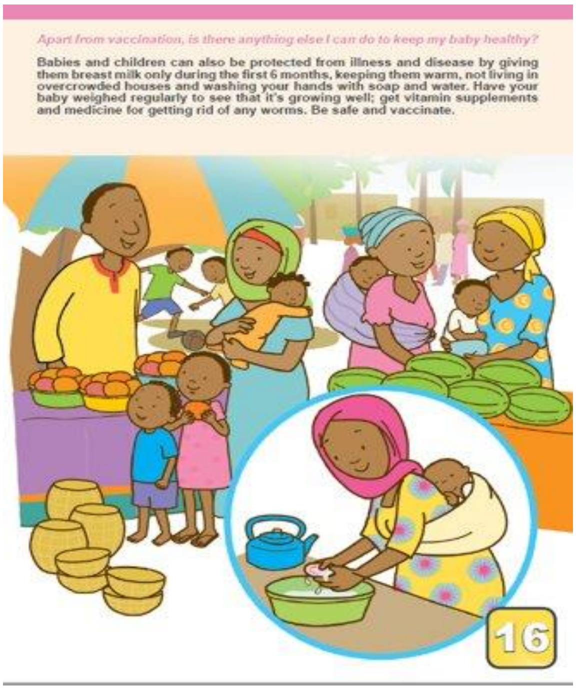

Page 16 of the SB
