# Supplementary material for: The use of a speaking book® to enhance vaccine knowledge among caregivers in The Gambia: A study using qualitative and quantitative methods
Source: BMJ Open. 2021 Mar 8;11(3):e040507. doi: 10.1136/bmjopen-2020-040507 (PMC7942236; doi:10.1136/bmjopen-2020-040507)
Supplement: Supplementary data [file bmjopen-2020-040507supp002.pdf]

BMJ Publishing Group Limited (BMJ) disclaims all liability and responsibility arising from any reliance placed on this supplemental material which has been supplied by the author(s)

Supplementary material 2_Questionnaire at baseline visit

Version 2.1 – 29th January 2019

MRC Unit The Gambia at the London School of Hygiene and Tropical Medicine

Study Title: The Vaccines Speaking Book Project

SCC:	1598	Version:	2.1

Sponsor & Funder: MRC & Bull City Learning

Primary Care Givers for the Implementation Study: Section A

Record ID: ____________________

Facility name: ____________________

Field worker initials: ____________________

Participants ID: ____________________

Date of entry: ____________________

Primary Care Givers (PCGs)

1. Full Name: ____________________
2. Contact details (Please collect all available numbers):
   ____
   ____
3. Detailed Address (You can draw behind):
   ____
   ____
4. Age (years): ____________________
5. Sex:
   - ☐ 1. Female
   - ☐ 2. Male
6. Religion:
   - ☐ Muslim
   - ☐ Christian
   - ☐ Others

6a. If others, specify ____________________

7. Marital status:
   - ☐ Single
   - ☐ Married
   - ☐ Widowed
   - ☐ Others

7a. If others, specify ____________________

8. Highest level of Education (Primary care giver):
   - ☐ No education
   - ☐ Primary (Grade 1-6)

Version	1.2	Date	8th January 2019

Nkereuwem OO, *et al. BMJ Open* 2021; 11:e040507. doi: 10.1136/bmjopen-2020-040507

☐ Secondary (Grade 7–9)
☐ Higher Secondary (Grade 10–12)
☐ Tertiary (University education)
☐ Others

8a. If others, specify ____________________________________

9. Education level of other parent (If applicable): ____________________________________
10. Employment status: ☐ Employed ☐ Self Employed ☐ Unemployed
11. Employment status of other parent (If applicable): ☐ Employed ☐ Self Employed ☐ Unemployed
12. Occupation: ____________________________________
13. Occupation of other parent if applicable: ____________________________________
14. Monthly household income: ☐ I don't know ☐ Below D2500 ☐ D2, 500-D5, 000 ☐ D5, 000- D10, 000 ☐ D10, 000- D20, 000 ☐ Above D20, 000
15. Presence of a working radio in the house: ☐ Yes ☐ No
16. Presence of a working television in the house: ☐ Yes ☐ No
17. Family size (number of people eating from one pot): ____________________________________
18. Mothers age at marriage (years): ____________________________________
19. Age at first child (years): ____________________________________

IMMUNISATION

20. Mothers receipt of Tetanus Toxoid during pregnancy: ☐ Yes ☐ No
21. How many times: ____________________________________
22. Distance from home to Health Facility (Minutes/hours): ____________________________________
23. Source of information regarding Immunisation: ☐ Healthcare Worker ☐ Information written on the vaccination cards ☐ Family member or friends ☐ Radio/TV ☐ Poster ☐ Others

23a. If others, specify: ____________________________________

SCC:	1598	Version v 1.2

24. What do you consider to be the challenge or problem you face attending immunization clinic

____

____

CHILD

25. Age: ______________________________
26. Date of Birth: (DD/MON/YEAR) ______________________________
27. Sex: ☐ Female ☐ Male
28. Birth order: ______________________________
29. Date of starting vaccination: ______________________________
30. Evidence of completeness of immunizations if available: ☐ Yes ☐ No ☐ Information not available
31. Place of Delivery of this child: ☐ Home ☐ Health Facility ☐ Others

31a. If others, specify: ______________________________

32. Mother's utilization of ANC during the pregnancy: ☐ Yes ☐ No
33. How many visits? ______________________________

Nkereuwem OO, *et al. BMJ Open* 2021; 11:e040507. doi: 10.1136/bmjopen-2020-040507

SCC:	1598	Version v 1.2

Primary Care Givers for the Implementation Study: Section B

Record ID: ______________________________

Facility name: ______________________________

Field worker initials: ______________________________

Participants ID: ______________________________

Date of entry: ______________________________

1. How do vaccines work?
   - ☐ Vaccines help a baby or a pregnant woman's body fight illness and diseases
   - ☐ Vaccines work by making security guards in the body called 'anti-bodies'
   - ☐ I don't know
   - ☐ Others

1a. If others specify: ______________________________

____

____

2. Why are vaccines given to pregnant women?
   - ☐ Vaccines ensure mother and child are protected against infectious diseases
   - ☐ If the pregnant woman is vaccinated, the baby is also protected for a little while after birth
   - ☐ I don't know
   - ☐ Others

2a. If others, specify: ______________________________

____

____

3. What do you do if your child is not well on the day the vaccines need to be given?
   - ☐ I will take my child to the hospital for the doctor or nurse to decide if my child can be immunised
   - ☐ I will wait at home until my child is well
   - ☐ I don't know
   - ☐ Others

3a. If others, specify: ______________________________

____

____

4. Do you think it is proper for your child to be given more than one vaccine at a visit?
   - ☐ Yes, different vaccines prevent different infections/diseases
   - ☐ No, administering more than one vaccine at a time prevents the vaccine from working effectively
   - ☐ I don't know
   - ☐ Others

4a. If others, specify: ______________________________

____

____

5. Why do you think your child is sometimes given the same vaccines more than once?
   - ☐ Repeat doses are needed to make the antibodies (body soldiers) stronger
   - ☐ Repeat doses help the baby's body learn to be strong against infections for a long time
   - ☐ I don't know
   - ☐ Others

Nkereuwem OO, *et al. BMJ Open* 2021; 11:e040507. doi: 10.1136/bmjopen-2020-040507

SCC:	1598	Version v 1.2

5a. If others, specify: ____
____
____

6. When do I need to consider the adverse event of a vaccine serious?
   - ☐ If my baby has high fever for more than a day
   - ☐ If my baby is sleeping a lot
   - ☐ I don't know
   - ☐ Others

6a. If others, specify: ____
____
____

7. What vaccines does your baby receive at each visit? Tick all applicable

7a. At birth
- ☐ Tuberculosis
- ☐ Polio
- ☐ Hepatitis B
- ☐ Diphtheria
- ☐ Whooping Cough
- ☐ Meningitis
- ☐ Pneumonia
- ☐ Diarrhoea
- ☐ Measles
- ☐ Yellow Fever
- ☐ I don't know

7b. At 2 Months
- ☐ Tuberculosis
- ☐ Polio
- ☐ Hepatitis B
- ☐ Diphtheria
- ☐ Whooping Cough
- ☐ Meningitis
- ☐ Pneumonia
- ☐ Diarrhoea
- ☐ Measles
- ☐ Yellow Fever
- ☐ I don't know

7c. At 3 Months
- ☐ Tuberculosis
- ☐ Polio
- ☐ Hepatitis B
- ☐ Diphtheria
- ☐ Whooping Cough
- ☐ Meningitis
- ☐ Pneumonia
- ☐ Diarrhoea
- ☐ Measles
- ☐ Yellow Fever
- ☐ I don't know

7d. At 4 Months
- ☐ Tuberculosis
- ☐ Polio

SCC:	1598	Version v 1.2

- ☐ Hepatitis B
- ☐ Diphtheria
- ☐ Whooping Cough
- ☐ Meningitis
- ☐ Pneumonia
- ☐ Diarrhoea
- ☐ Measles
- ☐ Yellow Fever
- ☐ I don't know

7e. At 9 Months

- ☐ Tuberculosis
- ☐ Polio
- ☐ Hepatitis B
- ☐ Diphtheria
- ☐ Whooping Cough
- ☐ Meningitis
- ☐ Pneumonia
- ☐ Diarrhoea
- ☐ Measles
- ☐ Yellow Fever
- ☐ I don't know

7f. At 18 Months

- ☐ Tuberculosis
- ☐ Polio
- ☐ Hepatitis B
- ☐ Diphtheria
- ☐ Whooping Cough
- ☐ Meningitis
- ☐ Pneumonia
- ☐ Diarrhoea
- ☐ Measles
- ☐ Yellow Fever
- ☐ I don't know

8. What other ways can you keep your baby healthy apart from vaccination?
   - ☐ Exclusive breast feeding for 6 months
   - ☐ Keeping baby warm
   - ☐ Regular hand washing with soap and water
   - ☐ Regular weighing of the baby at the clinic
   - ☐ Others

8a. If others, specify: ____
____
____

Nkereuwem OO, *et al. BMJ Open* 2021; 11:e040507. doi: 10.1136/bmjopen-2020-040507
