# Supplementary material for: The use of a speaking book® to enhance vaccine knowledge among caregivers in The Gambia: A study using qualitative and quantitative methods
Source: BMJ Open. 2021 Mar 8;11(3):e040507. doi: 10.1136/bmjopen-2020-040507 (PMC7942236; doi:10.1136/bmjopen-2020-040507)
Supplement: Supplementary data [file bmjopen-2020-040507supp003.pdf]

Supplementary material 3_Questionnaire at follow-up visits

Version 2.1 – 29th January 2019

MRC Unit The Gambia at the London School of Hygiene and Tropical Medicine

Study Title: The Vaccines Speaking Book Project

SCC:	1598	Version:	2.1

Sponsor & Funder: MRC & Bull City Learning

Primary Care Givers for the Implementation Study: Section B

Record ID: ____________________

Facility name: ____________________

Field worker initials: ____________________

Participants ID: ____________________

Date of entry: ____________________

1. How do vaccines work?
   - ☐ Vaccines help a baby or a pregnant woman's body fight illness and diseases
   - ☐ Vaccines work by making security guards in the body called 'anti-bodies'
   - ☐ I don't know
   - ☐ Others

1a. If others specify: ____________________

2. Why are vaccines given to pregnant women?
   - ☐ Vaccines ensure mother and child are protected against infectious diseases
   - ☐ If the pregnant woman is vaccinated, the baby is also protected for a little while after birth
   - ☐ I don't know
   - ☐ Others

2a. If others, specify: ____________________

3. What do you do if your child is not well on the day the vaccines need to be given?
   - ☐ I will take my child to the hospital for the doctor or nurse to decide if my child can be immunised
   - ☐ I will wait at home until my child is well
   - ☐ I don't know
   - ☐ Others

3a. If others, specify: ____________________

4. Do you think it is proper for your child to be given more than one vaccine at a visit?
   - ☐ Yes, different vaccines prevent different infections/diseases

Version	1.2	Date	8th January 2019

Nkereuwem OO, *et al. BMJ Open* 2021; 11:e040507. doi: 10.1136/bmjopen-2020-040507

SCC:	1598	Version v 1.2

- ☐ No, administering more than one vaccine at a time prevents the vaccine from working effectively
- ☐ I don't know
- ☐ Others

4a. If others, specify: ____________________

5. Why do you think your child is sometimes given the same vaccines more than once?
   - ☐ Repeat doses are needed to make the antibodies (body soldiers) stronger
   - ☐ Repeat doses help the baby's body learn to be strong against infections for a long time
   - ☐ I don't know
   - ☐ Others

5a. If others, specify: ____________________

6. When do I need to consider the adverse event of a vaccine serious?
   - ☐ If my baby has high fever for more than a day
   - ☐ If my baby is sleeping a lot
   - ☐ I don't know
   - ☐ Others

6a. If others, specify: ____________________

7. What vaccines does your baby receive at each visit? Tick all applicable

7a. At birth
- ☐ Tuberculosis
- ☐ Polio
- ☐ Hepatitis B
- ☐ Diphtheria
- ☐ Whooping Cough
- ☐ Meningitis
- ☐ Pneumonia
- ☐ Diarrhoea
- ☐ Measles
- ☐ Yellow Fever
- ☐ I don't know

7b. At 2 Months
- ☐ Tuberculosis
- ☐ Polio
- ☐ Hepatitis B
- ☐ Diphtheria
- ☐ Whooping Cough
- ☐ Meningitis
- ☐ Pneumonia
- ☐ Diarrhoea
- ☐ Measles
- ☐ Yellow Fever
- ☐ I don't know

7c. At 3 Months
- ☐ Tuberculosis
- ☐ Polio
- ☐ Hepatitis B

Nkereuwem OO, *et al. BMJ Open* 2021; 11:e040507. doi: 10.1136/bmjopen-2020-040507

SCC:	1598	Version v 1.2

- ☐ Diphtheria
- ☐ Whooping Cough
- ☐ Meningitis
- ☐ Pneumonia
- ☐ Diarrhoea
- ☐ Measles
- ☐ Yellow Fever
- ☐ I don’t know

7d. At 4 Months

- ☐ Tuberculosis
- ☐ Polio
- ☐ Hepatitis B
- ☐ Diphtheria
- ☐ Whooping Cough
- ☐ Meningitis
- ☐ Pneumonia
- ☐ Diarrhoea
- ☐ Measles
- ☐ Yellow Fever
- ☐ I don’t know

7e. At 9 Months

- ☐ Tuberculosis
- ☐ Polio
- ☐ Hepatitis B
- ☐ Diphtheria
- ☐ Whooping Cough
- ☐ Meningitis
- ☐ Pneumonia
- ☐ Diarrhoea
- ☐ Measles
- ☐ Yellow Fever
- ☐ I don’t know

7f. At 18 Months

- ☐ Tuberculosis
- ☐ Polio
- ☐ Hepatitis B
- ☐ Diphtheria
- ☐ Whooping Cough
- ☐ Meningitis
- ☐ Pneumonia
- ☐ Diarrhoea
- ☐ Measles
- ☐ Yellow Fever
- ☐ I don’t know

8. What other ways can you keep your baby healthy apart from vaccination?
   - ☐ Exclusive breast feeding for 6 months
   - ☐ Keeping baby warm
   - ☐ Regular hand washing with soap and water
   - ☐ Regular weighing of the baby at the clinic
   - ☐ Others

SCC:	1598	Version v 1.2

8a. If others, specify: ____
____
____

SCC:	1598	Version v 1.2

Primary Care Givers for the Implementation Study: Section C

Record ID: ____

Facility name: ____

Field worker initials: ____

Participants ID: ____

Date of entry: ____

1. Did you find the book easy to use? Yes ☐ No ☐

1a. Can you please give reasons for your response above?

____
____
____
____

2. Did you like the pictures in the book? Yes ☐ No ☐

2a. Can you please give reasons for your response above?

____
____
____
____

3. Could you hear the person talking to you clearly? Yes ☐ No ☐

3a. Can you please give reasons for your response above?

____
____
____
____

4. Did you understand all the information that you were told in the book? Yes ☐ No ☐

4b. Can you please give reasons for your response above?

____
____
____
____

5. Did you find any information in the book useful? Yes ☐ No ☐

Nkereuwem OO, *et al. BMJ Open* 2021; 11:e040507. doi: 10.1136/bmjopen-2020-040507

SCC:	1598	Version v 1.2

5a. Can you please give reasons for your response above?

____
____
____
____

5b. If yes, what was the information you found most useful?

____
____
____
____

6. Did you trust the messages in the book? Yes ☐ No ☐

6a. Can you please give reasons for your response above?

____
____
____
____

7. Did you learn new information from the book? Yes ☐ No ☐

7a. Can you please give reasons for your response above?

____
____
____
____

8. How many times did you go through the whole book?
1 ☐
2-5 ☐
6-9 ☐
10 or more times ☐

9. Can you clearly explain the information in the book to your family and friends? Yes ☐ No ☐

9a. Can you please give reasons for your response above?

____
____
____
____

10. Do you think that the information in the book gives you all the information needed to make a decision to immunize your child? Yes ☐ No ☐

SCC:	1598	Version v 1.2

10b. Can you please give reasons for your response above?

____
____
____
____

11. Were there any problems you had when you were using the book? Yes ☐ No ☐

11b. If Yes, what problem(s) did you experience?

____
____
____
____

12. Do you think the book should include any other information or have any changes? Yes ☐ No ☐

12b. If Yes, what changes would you suggest?

____
____
____
____

13. Did you show the book to anyone in your Family? Yes ☐ No ☐

13a. If yes, how many people ______________

13b. Did you show the book to anyone in your Community? Yes ☐ No ☐

13c. If yes, how many people ______________

13d. Did you show the book to anyone in your Mosque/Church? Yes ☐ No ☐

13e. If yes, how many people ______________

13f. Did you show the book to anyone at work? Yes ☐ No ☐

13g. If yes, how many people ______________

13h. Did you show the book to anyone in your clinic or mosque? Yes ☐ No ☐

13i. If yes, how many people ______________

13j. Can you please specify any other place you shared the book? ______________

14. Do you think members of your family, mosque/church, and community will understand the information in the book if they were given this book to listen to? Yes ☐ No ☐ I don't know ☐

SCC:	1598	Version v 1.2

14a. Can you please give reasons for your response above?

____
____
____
____

15. At what time do you think that the books should be given to a parent coming to get their child immunized?
    - At the time of first visit to the clinic only ☐
    - At first few visits to the clinic ☐
    - While speaking to the Health care worker ☐
    - Before speaking to the Healthcare Worker ☐
    - After speaking to the Healthcare worker ☐
    - Copy of the book is useful to stay with the parent at home ☐

16. Do you know where the on/off switch is in the book? Yes ☐ No ☐

17. Would you be able to change the battery? Yes ☐ No ☐

18. If you need to change the battery, do you have a screwdriver with which to do so? Yes ☐ No ☐
