# Supplementary material for: The use of a speaking book® to enhance vaccine knowledge among caregivers in The Gambia: A study using qualitative and quantitative methods
Source: BMJ Open. 2021 Mar 8;11(3):e040507. doi: 10.1136/bmjopen-2020-040507 (PMC7942236; doi:10.1136/bmjopen-2020-040507)
Supplement: Supplementary data [file bmjopen-2020-040507supp004.pdf]

Supplementary material 4_Interview guide sample for Health Care Workers

Version 2.1 – 29th January 2019

MRC Unit The Gambia at the
London School of Hygiene
and Tropical Medicine

Questionnaire for Health Care Workers (HCPs)/ Heads of the Health Centres where the SB invention was implemented

Record ID: ___________________________________

Facility name: ___________________________________

Field worker initials: ___________________________________

Participants ID: ___________________________________

Date of entry: ___________________________________

1. Age: ____________________
2. Gender: Female ☐ Male ☐
3. Time of working in the current position (years): ____________________
4. Do HCWs have enough time to make sure that Primary Care Givers (PCG) completely understand all the information explained to them about immunization? Yes ☐ No ☐

4a. If No, why not?

____
____
____
____

5. Did the book help you in addressing the Primary Care Givers (PCGs) questions and concerns? Yes ☐ No ☐

5a. Can you please give reasons for your response above?

____
____
____
____

6. Did the book make more efficient use of your time and energy? Yes ☐ No ☐

6a. Can you please give reasons for your response above?

____
____
____
____

Version	2.1	Date	8th January 2019

Nkereuwem OO, *et al. BMJ Open* 2021; 11:e040507. doi: 10.1136/bmjopen-2020-040507

SCC:	1598	Version v 2.1

7. Did the book increase knowledge of the PCG? Yes ☐ No ☐ I don't know ☐

7a. Can you please give a reason for your answer above?

____
____
____
____

8. Did the book increase communication between the PCG and you? Yes ☐ No ☐

8a. Can you please give a reason for your answer above?

____
____
____
____

9. Was there any other impact of the speaking book on the Primary Care Givers? Yes ☐ No ☐

If yes, what was the impact?

____
____
____

9a. Did the Primary Care Givers (PCGs) ask more questions? Yes ☐ No ☐

9b. Did you observe timelier Immunisation done of their child by the PCG? ` Yes ☐ No ☐ I don't know ☐

9c. Other observations? (Please mention)

____
____
____

10. When do you think the SB should be given to the PCGs?
- At the time of first visit to the clinic only ☐
- At first few visits to the clinic ☐
- While speaking to the Health care worker ☐
- Before speaking to the Healthcare Worker ☐
- After speaking to the Healthcare worker ☐
- Copy of the book is useful to stay with the parent at home ☐

11. Do you think the book should include any other information or have any changes? Yes ☐ No ☐

11a. What information would you suggest to be added?

____
____
____
____

Version	2.1	Date	8th January 2019
