# Supplementary material for: The use of a speaking book® to enhance vaccine knowledge among caregivers in The Gambia: A study using qualitative and quantitative methods
Source: BMJ Open. 2021 Mar 8;11(3):e040507. doi: 10.1136/bmjopen-2020-040507 (PMC7942236; doi:10.1136/bmjopen-2020-040507)
Supplement: Supplementary data [file bmjopen-2020-040507supp005.pdf]

Supplementary material 5_Themes, sub-themes and quotes

| Themes | Subthemes | Supporting quotes |
|---|---|---|
| General Challenges at the Clinics, independent of the SBs | Delivering Health Education | "… we normally conduct pre-clinic health talks… we discuss vaccines, and sometimes we choose other topics…. about their health and that of their babies" **(Male, Rural clinic, 18 months in current position)**<br><br>"When we have a large crowd, we also engage them in interpersonal communication while administering the vaccines, we tell them about the vaccines been administered and the possible side effects of the vaccine"**(Male, Rural clinic, 6 months in current position)** |
| | Attitude of caregiver | "When women come to the clinic, they are always in hurry to go back home to cook or going back to the garden, so most times they are in haste to go home" **(Male, Urban clinic, 5 years in current position)**<br><br>"What we do sometimes is to hold their cards and start weighing, and then when most of the women are around, we start giving the health education" **(Male, Rural clinic, 8 months in current position)** |
| | Organizational factors | "…most of them [caregivers] come around the same time and the health care worker might not have time to carefully explain in detail to the women … most times the environment is even very noisy" **(Male, Rural clinic, 3years in current position)**<br><br>"…. some of the women will be standing and distracted by the cry of their children and may not even have the patience to listen to what we are saying to them" Adding that: "Interpersonal health education is usually better for us with the crowd (High numbers of caregivers)" **(Female, Rural clinic, 3 years in current position)** |
| | Low manpower | "… when we started the implementation of the speaking books, I was the only Public Health Officer here without any form of assistance or back up." **(Male, Urban clinic, 5 years in current position)**<br><br>"Sometimes I will be the only one immunizing, weighing and screening so sometimes it's difficult to use the speaking books" **(Male, Urban clinic, 5 years in current position)** |
| SB Impact | Improved Knowledge | "Most times after listening to the books, we ask them questions on immunisation and they are able to give positive feedback" **(Male, Urban clinic, 18 months in current position)**<br><br>"… I cannot assure you on their [all caregivers attending the immunisation clinic] increase in knowledge compared to women who have personal copies [recruited participants] with them at home." **(Male, Rural clinic, 8months in current position)** |
| | Efficiency | "…for me it saves time because I just hand over the book to them [Caregiver] and then they listen to everything …. while I concentrate on the work and it makes work faster." **(Female, Rural clinic, 3 years in current position)** |

| | | |
|---|---|---|
| | | "… the issue is we have just one copy in the facility which …. is not enough…. they all [Caregiver] have to keep waiting for each other to listen to the books… if we have at least 5 copies it would be better than everyone sharing one copy." **(Male, Urban clinic, 18 months in current position)** |
| | Informative | "In the past they give so many complains about adverse effects following vaccination……. even blame public health officers that administer the vaccines, they have a better understanding of the side effects after receiving the vaccines and appreciate the information gotten from the book" **(Female, Rural clinic, 3 years in current position)**<br>"It has addressed all the details and relevant information that will make mothers want to bring their children for immunization" **(Female, Rural clinic, 3 years in current position)** |
| | Improved Clinic Attendance | "Mothers are beginning to bring their babies to the clinic unlike before when they will just come to the clinic anytime they like" **(Female, Urban clinic, 10 years in current position)**<br>"In the past we used to have low coverages but with the introduction of the speaking books are now seeing more mothers coming" **(Male, Urban clinic, 5 years in current position)** |
| Outcome of use of the SBs | Enhanced Communication | "The book has made it easier for us to explain information on vaccines to the mothers…" **(Female, Urban clinic, 10 years in current position)**<br>"… most of the women don't understand English but with the use of the local languages they understand the information easily……. it has been easier for us to pass across the information because English is not our language, so it is easier for them to understand and easier for us to explain to them" **(Male, Urban clinic, 3 years in current position)** |
| | Health Promotion Tool | "… we are trying, by recording the audio on our phones and sharing with other health staff because not all health officers have access to the information in this book…. we learnt a lot from it." **(Male, Urban clinic, 3 years in current position)**<br>"…… because the book is not loud for all the mothers to hear, I recorded it on my phone, used a Bluetooth device to connect to a speaker for all the mothers to hear" **(Male, Urban clinic, 2 years in current position)** |
| | Shared responsibility and community ownership | "They share during lunch or when they sit together, they play the book" **(Male, Rural clinic, 8months in current position)**<br>"During the immunisation clinic days most of the women who have copies of the speaking books come along with the books and they normally take over the health talks with the use of their speaking books, sometimes I don't even play my own"**(Male, Urban clinic, 5 years in current position)** |
| Further Suggestions | SB utilization | "I think is better they are given the books at their homes …. in the clinic most times they are in hurry to go home …. at home they will take out time to listen to the book and gain more information compared to listening to the books in the clinic" **(Male, Urban clinic, 5 years in current position)** |

| | | |
|---|---|---|
| | | "…. I believe the women will make better use of the speaking books if they are used in the facilities than in their homes ... at the health facility the health workers will be around to guide them, this will ensure a better use of the book…." **(Male, Urban clinic, 18 months in current position)** |
| | Changes to the SB | "The only problem the speaking book has is that it cannot be used with a large audience because the volume is very low" **(Female, Rural clinic, 3years in current position)**<br><br>"….. it will be good to have a book that is very loud so we don't have to talk, we will just place it at the front of the Clinic, press the buttons and the book will start playing for the women to listen to it" **(Male, Rural clinic, 6 months in current position)** |
| | Additions to the SB | "…other languages need to be added…. because caregivers who do not understand any of the languages used in the book did not benefit from the information in the book…." **(Male, Urban clinic, 4 years in current position)**<br><br>"…. we can also consider adding information on nutrition and importance of antenatal care by pregnant women" **(Female, Urban clinic, 10 years in current position** |
