# Supplementary material for: The use of a speaking book® to enhance vaccine knowledge among caregivers in The Gambia: A study using qualitative and quantitative methods
Source: BMJ Open. 2021 Mar 8;11(3):e040507. doi: 10.1136/bmjopen-2020-040507 (PMC7942236; doi:10.1136/bmjopen-2020-040507)
Supplement: Author's manuscript [file bmjopen-2020-040507.draft_revisions.pdf]

BMJ Open is committed to open peer review. As part of this commitment we make the peer review history of every article we publish publicly available.

When an article is published we post the peer reviewers' comments and the authors' responses online. We also post the versions of the paper that were used during peer review. These are the versions that the peer review comments apply to.

The versions of the paper that follow are the versions that were submitted during the peer review process. They are not the versions of record or the final published versions. They should not be cited or distributed as the published version of this manuscript.

BMJ Open is an open access journal and the full, final, typeset and author-corrected version of record of the manuscript is available on our site with no access controls, subscription charges or pay-per-view fees (http://bmjopen.bmj.com).

If you have any questions on BMJ Open's open peer review process please email info.bmjopen@bmj.com

BMJ Open

“Getting the message across” - The use of a Speaking Book to enhance vaccine knowledge among caregivers in The Gambia

Journal:	*BMJ Open*
Manuscript ID	bmjopen-2020-040507
Article Type:	Original research
Date Submitted by the Author:	26-May-2020
Complete List of Authors:	Nkereuwem, Oluwatosin; MRC Unit The Gambia at London School of Hygiene and Tropical Medicine, Vaccines and Immunity Theme<br>Kochhar, Sonali; Global Healthcare Consulting, New Delhi, India., Global Health; Department of Global Health, University of Washington, Seattle, USA, Global Health<br>Wariri, Oghenebrume; MRC Unit The Gambia at London School of Hygiene and Tropical Medicine, Vaccines and Immunity Theme<br>Johm, Penda; MRC Unit The Gambia at London School of Hygiene and Tropical Medicine, Vaccines and Immunity Theme<br>Ceesay, Amie; MRC Unit The Gambia at London School of Hygiene and Tropical Medicine, Vaccines and Immunity Theme<br>Kinteh, Mamanding; Expanded Programme on Immunization, Ministry of Health and Social Welfare, The Gambia<br>Kampmann, Beate ; Vaccines and Immunity Theme, Medical Research Council Unit The Gambia at London School of Hygiene and Tropical Medicine, Fajara, P.O. Box 273, Banjul, The Gambia., Vaccines and Immunity ; The Vaccine Centre, Faculty of Infectious Diseases, London School of Hygiene and Tropical Medicine, Keppel Street, London, UK, The Vaccine Centre
Keywords:	EPIDEMIOLOGY, Infection control < INFECTIOUS DISEASES, Public health < INFECTIOUS DISEASES, PREVENTIVE MEDICINE, PUBLIC HEALTH

SCHOLARONE™
Manuscripts

I, the Submitting Author has the right to grant and does grant on behalf of all authors of the Work (as defined in the below author licence), an exclusive licence and/or a non-exclusive licence for contributions from authors who are: i) UK Crown employees; ii) where BMJ has agreed a CC-BY licence shall apply, and/or iii) in accordance with the terms applicable for US Federal Government officers or employees acting as part of their official duties; on a worldwide, perpetual, irrevocable, royalty-free basis to BMJ Publishing Group Ltd ("BMJ") its licensees and where the relevant Journal is co-owned by BMJ to the co-owners of the Journal, to publish the Work in this journal and any other BMJ products and to exploit all rights, as set out in our licence.

The Submitting Author accepts and understands that any supply made under these terms is made by BMJ to the Submitting Author unless you are acting as an employee on behalf of your employer or a postgraduate student of an affiliated institution which is paying any applicable article publishing charge ("APC") for Open Access articles. Where the Submitting Author wishes to make the Work available on an Open Access basis (and intends to pay the relevant APC), the terms of reuse of such Open Access shall be governed by a Creative Commons licence – details of these licences and which Creative Commons licence will apply to this Work are set out in our licence referred to above.

Other than as permitted in any relevant BMJ Author's Self Archiving Policies, I confirm this Work has not been accepted for publication elsewhere, is not being considered for publication elsewhere and does not duplicate material already published. I confirm all authors consent to publication of this Work and authorise the granting of this licence.

For peer review only

"GETTING THE MESSAGE ACROSS" - THE USE OF A SPEAKING BOOK TO ENHANCE VACCINE KNOWLEDGE AMONG CAREGIVERS IN THE GAMBIA

Oluwatosin Nkereuwem[1], Sonali Kochhar [2,3], Oghenebrume Wariri[1], Penda Johm[1,5], Amie Ceesay[1], Mamanding Kinteh[4], Beate Kampmann[1,5]

[1] Vaccines & Immunity Theme, MRC Unit the Gambia at The London School of Hygiene and Tropical Medicine, The Gambia

[2] Global Healthcare Consulting, New Delhi, India

[3] Department of Global Health, University of Washington, Seattle, USA

[4] Expanded Programme on Immunization, Ministry of Health and Social Welfare, The Gambia

[5] The Vaccine Centre, Faculty of Infectious Diseases, London School of Hygiene and Tropical Medicine, Keppel Street, London, UK

Correspondence to:

Prof. Beate Kampmann, Vaccines and Immunity Theme, Medical Research Council Unit The Gambia at London School of Hygiene and Tropical Medicine, Fajara, P.O. Box 273, Banjul, The Gambia.

Email: bkampmann@mrc.gm

Keywords:

Vaccine knowledge; Caregiver knowledge; Communication tools; Multi-media educational tool; Expanded Programme on Immunization (EPI)

For peer review only - http://bmjopen.bmj.com/site/about/guidelines.xhtml

ABSTRACT

Introduction

Worldwide, caregiver knowledge on maternal and childhood vaccines is limited, particularly in underprivileged communities with high illiteracy rates. New approaches to improve vaccine knowledge, decision-making and enable vaccine communication between caregivers and health care workers are therefore needed.

Methods

We developed a multi-media educational tool, the vaccine *Speaking Book*®, which contained pre-recorded information in two Gambian languages, *Wolof* and *Mandinka* about the vaccines provided in The Gambia's Expanded Programme on Immunization (EPI). Accompanying illustrations were designed to reflect the local context. Content and illustrations were developed in collaboration with the national EPI team, caregivers attending EPI clinics and health care workers (HCW) using focus group discussions to gain feedback, ensuring that the *Speaking Book*® reflected the local Gambian context. A sequential mixed-methods approach was used to assess the impact of the *Speaking Book*® as a health education tool on both caregivers and HCW in The Gambia. We assessed caregivers baseline knowledge about vaccines in 15 peripheral health centres throughout The Gambia that had not previously been involved in vaccine-related research. Caregivers were then given the *Speaking Book*® to take home and we repeated the same assessments one and three-months later. We also conducted structured interviews with the caregivers and HCW at the vaccine clinics.

Results

One hundred and thirteen caregivers participated. Compared to the baseline knowledge, mean vaccine-related knowledge doubled at one-month and tripled at three-months after receiving the *Speaking Book*®. Qualitative results showed high acceptability and enthusiasm for this tool among both caregivers and HCW. The *Speaking Book*® was widely shared in the community and facilitated communication with HCW at clinic level.

Conclusion

Context and subject specific *Speaking Books*® are a useful communication and educational tool to increase caregiver vaccine knowledge in low- and middle-income countries.

For peer review only - http://bmjopen.bmj.com/site/about/guidelines.xhtml

Strengths and limitations of this study

- The format and content drafted to fit into the Gambian context was well received by both health care workers and caregivers and facilitated communication about vaccination within communities.
- Although we encouraged the study participants to share the *SB* with their family and friends, we did not capture any effects on the overall vaccine knowledge at the community level. There was no control group that did not receive the books.
- We did not systematically measure the impact on subsequent uptake of immunization services.

For peer review only

INTRODUCTION

Immunization is one of the most effective public health interventions to control and prevent diseases.[1] Unfortunately, global immunization coverage rates remain sub-optimal: 13.5 million infants do not receive any vaccination and approximately 20 million infants never complete the recommended immunization schedule.[2]

Several factors may act as barriers to childhood immunization especially in low-and-middle income countries (LMIC) where caregivers often have low literacy levels.[3–6] These include caregivers lack of knowledge about the recommended immunization schedules and alternatives once their infants miss scheduled immunizations; concerns about vaccine safety and adverse events; infants being unwell at the time of the appointment; reluctance to receive multiple vaccinations at the same time; lack of trust in the medical community; being unable to remember the information provided and the date for the next immunisation visit; hesitancy in asking questions which might be perceived as trivial by health care workers (HCW); and HCW being too busy or lacking the knowledge or patience to answer the caregivers questions and addressing their concerns.[3,7–9]

The caregivers knowledge about vaccines influences their decision to access immunization services for their infants.[7,10] This decision in turn impacts on the uptake of vaccination services, and consequently, on the morbidity and mortality attributable to vaccine-preventable diseases (VPD).[8,9] Data from high-income countries (HIC) have shown that improving health awareness and knowledge of caregivers about disease prevention can improve health outcomes, especially among less literate populations.[13] This would be applicable especially in LMIC in sub-Saharan Africa where caregivers have been shown to have low knowledge about immunization and vaccine-related issues.[14]

Recently, audio-visual tools like the *Speaking Book*® (*SB*) have been developed and used to target HCW and care-seekers in human immunodeficiency virus (HIV) care and mental health services, with studies demonstrating their usefulness in the improvement of knowledge.[13,15,16] These studies have also shown that *SB* complements communication between HCW and health care seekers. In a previous study conducted in The Gambia, a clinical trials specific *SB* was shown to increase the knowledge of study participants, leading to a better informed decision about participation.[13] Therefore, using context-specific, pictorial depictions followed by recorded audio messages targeted at caregivers is an effective way to deliver messages about vaccines in general, especially in a low literacy setting such as The Gambia.[17]

For peer review only - http://bmjopen.bmj.com/site/about/guidelines.xhtml

In this study, we developed the vaccine *SB*, a new *SB* covering routinely recommended maternal and childhood vaccines. We assessed its use as an educational tool for enhancing knowledge, understanding and recall of key vaccine-related information among caregivers in The Gambia, as well as its acceptability and relevance as a health promotion tool for caregivers and HCW.

METHODS

Study design and setting

We conducted a sequential exploratory mixed-method study which enrolled caregivers and their infants attending immunization clinics in 15 purposively selected Primary Health Care (PHC) facilities (seven rural and eight urban) across four regions of The Gambia. The seven rural and eight urban PHC facilities had not previously participated in vaccine research studies or clinical trials. Based on existing records, the EPI team selected immunization facilities with poorer performance compared to expected national outcomes and lack of exposure to vaccine research activities. The project was conducted in close collaboration with the communication department of The Gambia Expanded Programme on Immunization (EPI). We defined a caregiver as an adult aged ≥18 years attending the immunisation clinic with an infant, and responsible for the day-to-day care of the child, including but not limited to biological parents. Immunization services are provided free-of-charge at all government facilities in The Gambia, and caregivers are encouraged to access the services closest to their home. Immunization-related information should be routinely delivered during immunization clinic days by public health officers (PHO). Ethical approval was obtained from The Gambia Government/MRC Joint Ethics Committee (SCC 1598). Patients or the public were not involved in the design, or conduct, or reporting, or dissemination plans of our research.

Approach

Quantitative methods

Data collection tools were informed by a wide literature review and a questionnaire designed and revised based on feedback from pre-tests carried out in two PHCs with caregivers and HCW (participants similar to the study's target population). The questionnaire consisted of three sections capturing: the socio-demographics of the caregiver, immunization history, experiences of the caregiver and socio-demographics of their child(ren);a series of multiple-choice questions assessing vaccines and immunization knowledge of the caregiver and;a series of open and close-ended questions on the experiences of the caregiver on the use of the *SB*.

For peer review only - http://bmjopen.bmj.com/site/about/guidelines.xhtml

Qualitative methods

A qualitative approach was used to explore the perception of HCW on the use of the *SB* as a health promotion tool when sharing vaccine information with caregivers during immunisation clinics. We carried out in-depth interviews (IDI) with HCW using general, open-ended and non-leading questions, including questions to determine challenges faced when sharing vaccine information with caregivers, impact of the *SB* tool on their work, outcomes since the utilization of the *SB*, and suggestions on the future use of the tool.

Study tool

The prototype of the richly illustrated, audio-visual educational tool called a *Speaking Book* (*SB*) was developed by a United States-based company (https://speakingbooks.com/), with whom we collaborated. The *SB* concept can be adapted for specific purposes, and in our case, we adapted the text, recording and illustrations to vaccines routinely delivered by the Gambia EPI program.

During an iterative pilot phase, we developed a bespoke *SB* version (Figure 1) and conducted three separate focus group discussions (FGD) with EPI programme managers, caregivers attending EPI clinics and HCW to ensure that the final version of the *SB* reflected the local Gambian context. The final version of the *SB* was an A4-size hard cover book consisting of 16 pages of colourful, culturally sensitive illustrations with short texts written in English and recorded narrations in the two most widely spoken local languages, *Wolof* and *Mandinka.* Each *SB* has a plastic panel with removable battery, which hosts a series of push buttons, each corresponding to a specific page in the *SB*. When activated, the push buttons trigger a soundtrack of the text on the relevant page. The soundtrack was narrated by two respected local actors, with the appropriate voice and tonal quality. The language could be selected via a switch button.

Participant selection and data collection

All study participants were sensitized on the study procedures and objectives. Purposive sampling strategy was applied to select participants who met the inclusion criteria of the study: caregivers with infants below six months of age attending PHC centres in The Gambia, who have been living in a given area for at least six months, able to communicate in either *Wolof* or *Mandinka,* and able to provide written consent.

For peer review only - http://bmjopen.bmj.com/site/about/guidelines.xhtml

Each caregiver participated in a baseline visit during which a pretested, interviewer-administered structured questionnaire was used to obtain socio-demographic information and to assess their baseline knowledge about childhood and maternal vaccines. Only one attempt was allowed for response to the questions. Questions from the questionnaire was broken down to a total of 39 questions. To assess the knowledge of caregivers, we computed a knowledge score which was calculated by assigning a score of 1 or 0 to each correctly or incorrectly answered question, with a maximum and minimum knowledge score of 39 and 0 respectively.

Subsequently, the mechanics of the *SB* were explained, and each caregiver was given a copy of the *SB* for use at home and each facility head also got a copy of the *SB* to be used in the facilities. Caregivers could listen to the *SB* as many times as they wished during the entire study period and were also encouraged to invite other people to listen along with them. Following receipt of the *SB,* the participants were requested to return to the PHCs after one month and three months for the researchers to assess their understanding, retention, utilisation of key information and experiences using the *SB*. During each of these follow-up visits, the caregivers were assessed using the same questionnaire, which also included qualitative questions.

After three months additional interviews were held with HCW and heads of the participating immunization clinics in order to assess their perception of the acceptability, potential efficacy and use of the *SB* as a health promotion tool for HCW delivering immunisation services in The Gambia. Qualitative data were audio recorded and transcribed by the research team.

Data analysis

The median knowledge scores were calculated at the baseline, 1-month and 3-month follow-up visits. We calculated the difference in the median of the knowledge scores between baseline and 1 month, and between baseline and 3 months for the entire cohort and by sub-groups using the Wilcoxon's matched-pairs signed-rank test for non-parametric paired data. We compared the difference in median score between groups (baseline and 1-month follow-up visits and baseline and 3-month follow-up visits). The distribution of the scores are presented in tables showing median scores and Inter-Quartile Range (IQR) as appropriate, with significant level set at $p<0.05$. All quantitative data were entered into Research Data Capture (**REDCap) for proper documentation and** analyses were performed using STATA version 13 (StataCorp, USA).

For peer review only - http://bmjopen.bmj.com/site/about/guidelines.xhtml

For the qualitative analysis, we applied inductive thematic analysis in deriving our themes and sub-themes as described by Braun et al.[18] We used NVivo® (version 12) software to organise the qualitative data during the analysis.

RESULTS

Between January and July 2019, we approached 200 caregivers during the initial visit, out of which a total of 113 (56%) agreed to participate (Figure 2). Of the 113 caregivers, 104 (92%) completed all 3 study visits, 108 (95.6%) completed the baseline and 1-month follow up visits, and 107 (94.7%) completed the baseline and 3-month follow up visits. Reasons for missed visits were ill-health of the mother, ill-health of infant and change in location.

The mean age of the caregivers was 26.7 years (±5.95). Most of the caregivers were married (n= 106, 93.8%), Muslim (n= 111, 98.2%), and unemployed (n=91, 80.5%). Over two-thirds of the caregivers reported that they lived within one hour of the immunization clinic, and most women (n=109, 96.5%) had received at least one dose of tetanus toxoid (TT) during their last pregnancy. Although none of the caregivers identified the media as their source of information regarding immunization, 90 (79.6%) households had a functional radio, and about half of them had a television in their homes. Over two-thirds of the caregivers acknowledged that HCW at the immunization clinics were their major source of information on immunization. Table 1 summarises the socio-demographic characteristics of the caregivers.

Table 1: Sociodemographic characteristics of Caregivers enrolled

Variable	N = 113	(%)
**Age (years)**		
<20	23	(20.4)
21-30	64	(56.6)
>30	26	(23.0)
**Religion**		
Muslim	111	(98.2)
Christian	2	(1.8)
**Region**		
Urban	52	(46.0)
Rural	61	(54.0)
**Marital status**		
Unmarried	7	(6.2)
Married	106	(93.8)
**Employment status**		
Employed	22	(19.5)
Unemployed	91	(80.5)
**Highest level of education**		
No Education	42	(37.2)
Primary	11	(9.7)
Secondary	21	(18.6)

For peer review only - http://bmjopen.bmj.com/site/about/guidelines.xhtml

Higher Secondary	21	(18.6)
Tertiary	2	(1.8)
Arabic/French	16	(14.2)
**Monthly household income**		
Below $50	19	(16.8)
$50-$100	38	(33.6)
$100-$200	14	(12.4)
Above $200	3	(2.7)
Don't Know	39	(34.5)
**Presence of working radio in the home**		
Yes	90	(79.6)
No	23	(20.4)
**Presence of working television in the home**		
Yes	56	(49.6)
No	57	(50.4)
**Reported distance to health facility**		
Less than 30 minutes	41	(36.3)
30 minutes to one hour	37	(32.7)
Over 1 hour	13	(11.5)
Not sure	22	(19.5)
**Mother's receipt of tetanus toxoid**		
Yes	109	(96.5)
No	4	(3.5)
**Usual source of information regarding immunization**		
Health workers	77	(68.1)
Infant welfare card	6	(5.3)
Family member or friend	4	(3.5)
Media	0	(0)
Not sure	26	(23.0)

Impact of *SB* on knowledge of caregivers

There was a significant increase in the median knowledge score from 6.0 (IQR 5.0-7.0) at baseline to 11.0 (IQR 8.0-14.0) at 1-month visit (P = <0.001), and 15.0 (IQR 10.0-20.0) at 3-month visit (P = <0.001). The highest median knowledge scores at 1-month and 3-month visits were seen among the caregivers above 30 years of age and families with monthly house-hold income above $100 (13.0 and 17.0 respectively). When analysed by subgroups (urban or rural, age of caregivers, household income or highest level of education), there was significant improvement in knowledge scores across all groups, as summarised in Table 2

For peer review only - http://bmjopen.bmj.com/site/about/guidelines.xhtml

Table 2: ***Median (IQR) of the knowledge scores at baseline and 1-month follow-up, as well as of the difference scores.***

Variable	N=108	Median baseline knowledge score [a]	Median knowledge score at 1-month	Median difference [c] (Baseline- 1-month score)	*P*	N=107	Median baseline Knowledge score [b]	Median knowledge score at 3-months	Median difference [c] (Baseline- 3-month score)	*P*
**All caregivers**	108	6.0 (5.0-7.0)	11.0 (8.0-14.0)	5.0 (2.0-8.0)	<0.0001	107	6.0 (5.0-7.0)	15.0 (10.0-20.0)	8.0 (5.0-14.0)	<0.0001
**Region**										
Rural	57	6.0 (5.0-7.0)	11.0 (8.0-15.0)	4.0 (2.0-9.0)	<0.0001	58	6.0 (5.0-7.0)	15.5 (10.0-21.0)	10.0 (5.0-15.0)	<0.0001
Urban	51	6.0 (4.5-7.0)	11.0 (7.0-14.0)	5.0 (2.0-7.0)	<0.0001	49	6.0 (4.5-7.0)	13.0 (11.0-19.0)	8.0 (5.0-12.0)	<0.0001
**Age (years)**										
<=20	23	6.0 (5.0-7.0)	9.0 (8.0-14.0)	4.0 (2.0-7.0)	0.0001	23	6.0 (5.0-7.0)	12.0 (8.0-19.0)	6.0 (2.0-14.0)	<0.0001
21-30	60	6.0 (5.0-7.0)	11.0 (7.0-14.0)	4.0 (2.0-8.0)	<0.0001	61	6.0 (5.0-7.0)	15.0 (11.0-21.0)	8.0 (5.0-14.0)	<0.0001
>30	25	6.0 (4.0-7.0)	13.0 (10.0-16.0)	6.0 (4.0-10.0)	<0.0001	23	6.0 (4.0-7.0)	16.0 (12.0-17.0)	9.0 (6.0-13.0)	<0.0001
**Household income**										
<$50	19	6.0 (5.0-7.0)	11.0 (10.0-14.0)	5.0 (4.0-8.0)	0.0002	18	6.0 (5.0-7.0)	15.5 (13.0-23.0)	9.5 (6.0-17.0)	0.0002
$50-100	38	6.0 (5.0-7.0)	11.0 (8.0-14.0)	5.0 (3.0-7.0)	<0.0001	37	6.0 (5.0-7.0)	14.0 (11.0-20.0)	8.0 (5.0-13.0)	<0.0001
>$100	15	6.0 (5.0-7.0)	12.0 (8.5-15.5)	7.0 (2.5-9.5)	0.0009	13	6.0 (5.0-7.0)	17.0 (14.0-20.0)	10.0 (7.0-13.0)	0.0004
Not sure	36	6.0 (5.0-6.0)	9.0 (7.0-15.0)	3.5 (1.5-9.5)	<0.0001	39	6.0 (5.0-6.0)	12.0 (8.0-20.0)	7.0 (3.0-15.0)	<0.0001
**Level of education**										
None	41	6.0 (5.0-7.0)	11.0 (8.0-15.0)	5.0 (2.0-10.0)	<0.0001	41	6.0 (5.0-7.0)	15.0 (11.0-21.0)	9.0 (5.0-15.0)	<0.0001
Primary	11	5.0 (4.0-6.0)	10.5 (8.0-15.0)	5.5 (4.0-9.0)	0.0057	11	5.0 (4.0-6.0)	16.5 (12.0-22.0)	12.0 (8.0-17.0)	0.005
Secondary	19	6.0 (5.0-7.0)	11.0 (9.0-12.0)	5.0 (3.0-7.0)	0.0001	19	6.0 (5.0-7.0)	13.0 (10.0-16.5)	7.5 (5.0-10.0)	0.0001
Higher sec./tertiary	22	6.0 (5.0-7.0)	10.5 (8.0-13.0)	4.5 (2.0-8.0)	0.0002	21	6.0 (5.0-7.0)	16.5 (10.0-23.0)	11.0 (5.0-16.0)	0.0001
Arabic/French	15	6.5 (6.0-7.0)	10.0 (7.0-15.0)	3.0 (1.5-8.5)	0.0048	15	6.0 (5.0-7.0)	13.0 (8.0-17.5)	6.0 (2.5-11.5)	0.0009

[a] *Median baseline knowledge score (median difference). Using the same participants present at both baseline visit and 1-month follow-up visit*
[b] *Median baseline knowledge score (median difference). Using the same participants present at both baseline visit and 3-month follow-up visit*
[c] *Since median (IQR) of the differences are reported, these might not match the difference of the medians at each time point.*

For peer review only - http://bmjopen.bmj.com/site/about/guidelines.xhtml

Opinions of the caregivers

In general, the *SB* was very well received by the caregivers, and their opinions are summarised in Table 3. The caregivers found the *SB* increasingly easy to use 103/108 (95.4%) at 1 month, versus 107/107 (100%) at 3 months respectively), and most of them stated that they understood all the information in the book 100/108 (92.6%) at 1 month vs 98/107; 91.6% at 3 months. Many participants also shared the *SB* in their communities and expressed trust in the information provided. The survey results also showed that using the book was easy, with only 10 (9.3%) and 7 (6.5%) caregivers reporting some problems at 1-month and 3-month visits respectively.

Table 3: Opinions of Caregivers about the Vaccine Speaking Book

**Questions**		**1-month visit n=108**	**(%)**	**3-month visit n=107**	**(%)**
Did you find the book easy to use?	Yes	103	95.4	107	100
	No	5	4.6	0	0
Did you like the pictures in the book	Yes	108	100	107	100
	No	0		0	0
Could you hear the person talking to you clearly?	Yes	108	100	107	100
	No	0		0	0
Did you understand all the information that you were told in the book	Yes	100	92.6	98	91.6
	No	8	7.4	9	8.4
Did you find the information in the book useful	Yes	107	99.1	106	99.1
	No	1	0.9	1	0.9
Did you trust the messages in the book?	Yes	108	100	106	99.1
	No	0	0	1	0.9
Did you learn new information from the book?	Yes	102	94.4	105	98.1
	No	6	5.6	2	1.9
How many times did you go through the whole book?	once	4	3.7	5	4.7
	2-5 times	40	37.0	33	30.8
	6-9 times	10	9.3	3	2.8
	≥10 times	54	50.0	66	61.7
Can you clearly explain the information in the book to your family and friends?	Yes	107	99.1	102	95.3
	No	1	1.0	5	4.7
Do you think that the information in the book gives you all the information needed to decide to immunize your child?	Yes	108	0	106	99.1
	No	0	0	1	0.9
Were there any problems you had when you were using the book?	Yes	10	9.3	7	6.5
	No	98	90.7	100	93.5
Do you think the book should include any other information or have any changes?	Yes	30	27.8	20	18.7
	No	78	72.2	87	81.3
Did you show the book to anyone in your Family?	Yes	107	99.1	106	99.1
	No	1	1.0	1	0.9
Did you show the book to anyone in your Community?	Yes	87	80.6	96	89.7
	No	17	15.7	11	10.3

For peer review only

For peer review only - http://bmjopen.bmj.com/site/about/guidelines.xhtml

	No Response	4	3.7	0	0.0
Did you show the book to anyone in your Mosque/Church?	Yes	4	3.7	2	1.9
	No	104	96.3	104	97.2
	No Response	0	0	1	0.9
Did you show the book to anyone at work?	Yes	21	19.4	16	15.0
	No	86	79.6	91	85.0
	No Response	1	0.9	0	0
Did you show the book to anyone at the clinic or hospital?	Yes	10	9.3	14	13.1
	No	95	88.0	90	84.1
	No Response	3	2.8	3	2.8
Do you think members of your family, mosque/church, and community will understand the information in the book if they were given this book to listen to?	Yes	106	98.1	100	93.5
	No	0	0.0	1	0.9
	I don't know	2	1.9	6	5.6
At what time do you think that the books should be given to a parent coming to get their child immunized?	At the time of first visit to the clinic only	82	75.9	102	95.3
	At first few visits to the clinic	11	10.2	5	4.7
	While speaking to the HCW	1	0.9	0	0
	Before speaking to the HCW	2	1.9	0	0
	After speaking to the HCW	10	9.3	0	0
	No Response	2	1.9	0	0
Do you know where the on/off switch is in the book?	Yes	108	100	107	100
	No	0	0.0	0	0
Would you be able to change the battery?	Yes	95	88.0	106	99.1
	No	11	10.2	1	0.9
	No Response	2	1.9	0	0
If you need to change the battery, do you have a screwdriver with which to do so?	Yes	83	76.9	97	90.7
	No	25	23.1	10	9.3

For peer review only

Themes from qualitative questions posed to Caregivers in questionnaire

In addition to the questions posed to caregivers on their experiences using the *SB,* below are reoccurring themes with supporting quotes from the open-ended part of the questionnaire:

- Information congruity

"I trust the messages in the book because the nurses tell me the same information as stated in the book." ***[Mother, 32 years old, French language certificate, urban dweller]***

- Clear presentation

"… because the book is written in English… and also clearly translated in two local languages." ***[Mother, 31 years old, Tertiary Education, urban dweller]***

"…the pictures make me believe the messages." ***[Mother, 30 years old, No Education, rural dweller]***

- Evidence of vaccine effectiveness

"… I trust the messages because some diseases we used to see…. [giving examples] have now drastically reduce." ***[Mother, 37 years old, Secondary (Grade 7–9), rural dweller]***

- Understanding information

"… I found it difficult understanding the book at first, but after some time I began to understand better" ***[Mother, 32 years old, Secondary (Grade 7–9), urban dweller]***

"… because I am not educated, I found the book difficult to learn despite the audio… I began to understand with the help of some students who read and explained to me." ***[Mother, 25 years old, Secondary (Grade 7–9), urban dweller]***

- Sense of responsibility

"… Initially I was afraid the book would go bad in my care but with time I was not afraid anymore" [Mother, 27 years old, No education, rural dweller]

- Challenges with the batteries

"… The battery died and then I had to change it" ***[Mother, 25 years old, Tertiary (University education), urban dweller]***

Themes and sub-themes from in-depth interviews with HCW

In addition to the evaluation of caregivers, IDI were conducted with 14/15 (93.3%) HCW at the immunization clinics where the study was carried out to assess their utilization and perceptions of the potential efficacy, acceptability and overall use of the *SB*. The mean age of the HCW interviewed was 33.4 years, and they were predominantly female (64.3%). One of the HCW could not be interviewed as they were absent from work due to ill-health. Themes and sub-themes are summarized in **Figure 3.**

For peer review only - http://bmjopen.bmj.com/site/about/guidelines.xhtml

Theme 1: General Challenges at the Clinics, independent of the *SB*s

Sub-theme 1.1: Delivering Health Education

"… we normally conduct pre-clinic health talks… we discuss vaccines, and sometimes we choose other topics…. about their health and that of their babies"

(Male, Rural clinic, 18 months in current position)

"When we have a large crowd, we also engage them in interpersonal communication while administering the vaccines, we tell them about the vaccines been administered and the possible side effects of the vaccine"

(Male, Rural clinic, 6 months in current position)

Sub-theme 1.2: Attitude of caregiver

"When women come to the clinic, they are always in hurry to go back home to cook or going back to the garden, so most times they are in haste to go home"

(Male, Urban clinic, 5 years in current position)

"What we do sometimes is to hold their cards and start weighing, and then when most of the women are around, we start giving the health education"

(Male, Rural clinic, 8 months in current position)

Sub-theme 1.3: Organizational factors

"…most of them [caregivers] come around the same time and the health care worker might not have time to carefully explain in detail to the women … most times the environment is even very noisy" ***(Male, Rural clinic, 3years in current position)***

"…. some of the women will be standing and distracted by the cry of their children and may not even have the patience to listen to what we are saying to them" Adding that: "Interpersonal health education is usually better for us with the crowd (High numbers of caregivers)"

(Female, Rural clinic, 3 years in current position)

Sub-theme 1.4: Low manpower

"… when we started the implementation of the speaking books, I was the only Public Health Officer here without any form of assistance or back up."

For peer review only - http://bmjopen.bmj.com/site/about/guidelines.xhtml

(Male, Urban clinic, 5 years in current position)

"Sometimes I will be the only one immunizing, weighing and screening so sometimes it's difficult to use the speaking books"

(Male, Urban clinic, 5 years in current position)

Theme 2: *SB* Impact

Sub-theme 2.1: Improved Knowledge

"Most times after listening to the books, we ask them questions on immunisation and they are able to give positive feedback"

(Male, Urban clinic, 18 months in current position)

"… I cannot assure you on their [all caregivers attending the immunisation clinic] increase in knowledge compared to women who have personal copies [recruited participants] with them at home."

(Male, Rural clinic, 8months in current position)

Sub-theme 2.2: Efficiency

"…for me it saves time because I just hand over the book to them [Caregiver] and then they listen to everything …. while I concentrate on the work and it makes work faster."

(Female, Rural clinic, 3 years in current position)

"… the issue is we have just one copy in the facility which …. is not enough…. they all [Caregiver] have to keep waiting for each other to listen to the books… if we have at least 5 copies it would be better than everyone sharing one copy."

(Male, Urban clinic, 18 months in current position)

Sub-theme 2.3: Informative

"In the past they give so many complains about adverse effects following vaccination…… even blame public health officers that administer the vaccines, they have a better understanding of the side effects after receiving the vaccines and appreciate the information gotten from the book"

(Female, Rural clinic, 3 years in current position)

For peer review only - http://bmjopen.bmj.com/site/about/guidelines.xhtml

"It has addressed all the details and relevant information that will make mothers want to bring their children for immunization" ***(Female, Rural clinic, 3 years in current position)***

Sub-theme 2.4: Improved Clinic Attendance

"Mothers are beginning to bring their babies to the clinic unlike before when they will just come to the clinic anytime they like" ***(Female, Urban clinic, 10 years in current position)***

"In the past we used to have low coverages but with the introduction of the speaking books are now seeing more mothers coming"

(Male, Urban clinic, 5 years in current position)

Theme 3: Outcome of use of the *SB*s

Sub-theme 3.1: Enhanced Communication

"The book has made it easier for us to explain information on vaccines to the mothers…"

(Female, Urban clinic, 10 years in current position)

"… most of the women don't understand English but with the use of the local languages they understand the information easily……. it has been easier for us to pass across the information because English is not our language, so it is easier for them to understand and easier for us to explain to them"

(Male, Urban clinic, 3 years in current position)

Sub-theme 3.2: Health Promotion Tool

"… we are trying, by recording the audio on our phones and sharing with other health staff because not all health officers have access to the information in this book…. we learnt a lot from it."

(Male, Urban clinic, 3 years in current position)

"…… because the book is not loud for all the mothers to hear, I recorded it on my phone, used a Bluetooth device to connect to a speaker for all the mothers to hear"

(Male, Urban clinic, 2 years in current position)

Sub-theme 3.3: Shared responsibility and community ownership

For peer review only

"They share during lunch or when they sit together, they play the book"

(Male, Rural clinic, 8months in current position)

"During the immunisation clinic days most of the women who have copies of the speaking books come along with the books and they normally take over the health talks with the use of their speaking books, sometimes I don't even play my own"

(Male, Urban clinic, 5 years in current position)

"… in the communities, mothers and caregivers look up to those who have benefitted from the study they are like mentors to other mothers and caregivers in the community where they live"

(Male, Rural clinic, 3years in current position)

Theme 4: Further Suggestions

Sub-theme 4.1: SB utilization

"I think is better they are given the books at their homes …. in the clinic most times they are in hurry to go home …. at home they will take out time to listen to the book and gain more information compared to listening to the books in the clinic"

(Male, Urban clinic, 5 years in current position)

"…. I believe the women will make better use of the speaking books if they are used in the facilities than in their homes ... at the health facility the health workers will be around to guide them, this will ensure a better use of the book…."

(Male, Urban clinic, 18 months in current position)

Sub-theme 4.2: Changes to the SB

"The only problem the speaking book has is that it cannot be used with a large audience because the volume is very low"

(Female, Rural clinic, 3years in current position)

"….. it will be good to have a book that is very loud so we don't have to talk, we will just place it at the front of the Clinic, press the buttons and the book will start playing for the women to listen to it"

(Male, Rural clinic, 6 months in current position)

For peer review only - http://bmjopen.bmj.com/site/about/guidelines.xhtml

Sub-theme 4.3: Additions to the SB

“. other languages need to be added…. because caregivers who do not understand any of the languages used in the book did not benefit from the information in the book….”

(Male, Urban clinic, 4 years in current position)

“.... we can also consider adding information on nutrition and importance of antenatal care by pregnant women” ***(Female, Urban clinic, 10 years in current position)***

DISCUSSION

In this study, we assessed the impact of a bespoke, multi-media educational tool, the vaccine *Speaking Book*®, on vaccine-related knowledge of caregivers as well as on communication between HCW and caregivers in The Gambia. We distributed this tool to 113 caregivers visiting PHC for routine immunization services and evaluated changes in vaccine knowledge over a 3-month period.

We saw significant improvement in the knowledge scores with the median scores nearly doubling at 1-month and nearly tripling at 3-month follow-up visits, compared to the baseline scores, independent of region of the country (urban or rural), age of caregiver, household income or highest level of education. The *SB* was given to the caregivers to take home, thus giving them several opportunities to listen to and understand the information in the book, which might have contributed to the ongoing improvement over the 3-month period.

Study participants discussed and shared content of the *SB* extensively within their family, community, and social groups, using a variety of platforms such as peer group meetings, community and religious gatherings. This is likely to lead to a significant and possibly measurable multiplier effect. This demonstrates the usefulness of the *SB* beyond individuals' knowledge gain and in both the health facility and community setting. Our findings of “added value” are similar to observations by other investigators who used a similar tool.[17,19]

Our results support the importance of providing targeted, vaccine-related education to caregivers and communities as an effective and practical strategy to improve vaccine-related knowledge in low literacy settings such as The Gambia. The *SB* can be a useful educational tool to achieve this.[20,21]

For peer review only - http://bmjopen.bmj.com/site/about/guidelines.xhtml

Our data is comparable to findings from a previous study conducted by our team in The Gambia[13] where the use of the *SB* equally resulted in significant improvement in knowledge gained among study participants. However, the previous speaking book was directed at providing information about a specific clinical trial of pneumococcal vaccines, not the EPI program and vaccine-related knowledge in general. It was also conducted in a community with previous exposure to vaccine research, a setting we deliberately avoided in the project presented here as it is likely to lead to significant bias. Illustrations in the previous *SB* had also not been adapted to the local Gambia context. During the stakeholder engagement for our study, we received feedback that led to specific alterations with regards to dress code for example, and to derive a *SB* that reflects local communities was an important message received.

While the *SB* was able to significantly improve the vaccine-related knowledge, a knowledge gap remained: although we reported significant improvements in the mean knowledge scores from a very low baseline, the highest score obtained was 19.0, still 20 points below the maximum possible score of 39. It is possible that the detailed knowledge needed to obtain these remaining points is more difficult to retain compared to some other answers and we wondered if there was an educational bias. The subgroup of caregivers with tertiary education did not achieve significantly higher scores, however, this group had had a higher baseline score. We are unable to draw firmer conclusions about the impact of higher education, since there were only two participants in this group. It is entirely possible that the knowledge score developed was overambitious.

A majority of the caregivers reported that HCW were their usual source of information about vaccines. Hence further training and retraining the HCW to deliver the additional information to the caregivers could significantly contribute to reducing the remaining knowledge gap[22]. In a study similar to ours, the *SB* was used regularly for health promotion in health posts, home visits and local gatherings and HCW referred to the *SB* as a “job aid”.[15]

Most caregivers had listened to the *SB* multiple times and suggested that it would be important for mothers to receive a copy of the *SB* at their first clinic visit, especially while pregnant or when their babies are newly born. Trust in the messages provided by the book was almost unanimous and the illustrations, which were adapted to the local context, were approved by all participants. The initial stakeholder consultation in the design phase of the book was therefore very important. We highly recommend to involve the local communities in the design of such tools, as their identification with the visual materials is likely to increase its acceptancy.[13,16]

For peer review only - http://bmjopen.bmj.com/site/about/guidelines.xhtml

Results obtained from the HCW interviews demonstrated that the *SB* had empowered them to answer concerns and questions of caregivers, emphasising the importance of the local language translations which presented vaccine information in a simple, easy to understand manner. HCW reported increase in productivity and improved clinic attendance and considered the *SB* as an important health promotion tool for both HCW and caregivers in the Gambia. It was evident that the *SB* had a positive impact on their day-to-day practice, and its content was well received, which facilitated communication on vaccines.

Vaccine confidence remains high in countries like The Gambia, but in the overall context of increasing vaccine hesitancy worldwide, tools such as the *SB* or similar approaches tailored to local context might add significant value to engage individuals and communities in communication about the personal and societal value of vaccination. They could be an important counter-measure to vaccine hesitancy.[23–25] It is interesting to note that none of the participants reported obtaining their vaccine-related information from the media, unlike in many high-income countries.

There are some clear limitations to our study: although we encouraged the study participants to share the *SB* with their family and friends, we did not capture any effects on the overall vaccine knowledge at the community level, and we did not systematically measure the impact on subsequent uptake of immunization services. In order to achieve these goals a controlled clinical trial would have been required, including a control group which did not receive *SBs*. This was beyond our means in this project. Further research is now required to quantify any potential effect on vaccine uptake at the health center level and measure cost-effectiveness. In discussions with the Gambian EPI program, both are expressed areas of interest. The *SB* used in this study was only recorded in two of the locally spoken languages (*Wolof* and *Mandinka*) in The Gambia and therefore, excluded caregivers who did not speak or understand either of these languages. We were asked to produce books in additional local languages in the future as well as design books for additional topics, such as pregnancy and nutrition.

CONCLUSION

In conclusion, the vaccine *Speaking Book*® is a health education tool that resulted in significant increase in vaccine-related knowledge of caregivers, irrespective of their age, location,

For peer review only - http://bmjopen.bmj.com/site/about/guidelines.xhtml

household income or educational level. It also served as a valuable tool for HCW in their interactions with caregivers and might lead to increased uptake of immunization services.

PATIENT AND PUBLIC INVOLVEMENT STATEMENT

This study was entirely outward-facing and the pilot phase in which the content of the book was shaped was informed by our focus groups with members of the public. There are no formal patient and public involvement groups in The Gambia.

EXTRA DATA SHARING

Extra qualitative data is available on request by contacting the corresponding author.

CONTRIBUTORS

BK and SK conceived the study design; ON, OW and AC collected and led analysis of the data; BK and ON led write-up; BK, SK, OW, PJ and MK provided critical feedback and helped shape the research, analysis and manuscript. All authors have approved the final version.

ACKNOWLEDGEMENTS

We thank the field assistants for detailed data collection and transcription from the local languages to English for data analysis. We thank Alexander Jarde for statistical support in the analysis of the study data. We also thank the heads of the participating health facilities and members of staff for granting us access to the EPI clinics and their support throughout the project. We thank The Gambian ministry of health for their contribution to content of the speaking book.

COMPETING INTERESTS

None

FUNDING

This study was funded by a project grant from Bull City Learning.

BK is supported by funding from the UKRI (MC_UP_A900/1122). The Vaccines & Immunity research led by BK is jointly funded by the UK Medical Research Council (MRC) and the UK Department for International Development (DFID) under the MRC/DFID Concordat agreement and is also part of the EDCTP2 programme supported by the European Union.

For peer review only - http://bmjopen.bmj.com/site/about/guidelines.xhtml

REFERENCES

1. Lee Ventola C. Immunization in the United States: Recommendations, barriers, and measures to improve compliance: Part 1: Childhood vaccinations. P T. 2016 Jul;41(7):426–36.

2. Progress and Challenges with Achieving Universal Immunization Coverage 2018 WHO/UNICEF Estimates of National Immunization Coverage. 2019.

3. Esposito S, Principi N, Cornaglia G. Barriers to the vaccination of children and adolescents and possible solutions. Clin Microbiol Infect. 2014 May;20:25–31.

4. Sato R, Fintan B. Fear, knowledge, and vaccination behaviors among women in Northern Nigeria. Hum Vaccin Immunother [Internet]. 2020 Feb 21 [cited 2020 Mar 10];1–11. Available from: https://www.tandfonline.com/doi/full/10.1080/21645515.2020.1723365

5. Ianni A, Tedeschi R, Marchetti A, Basso D, Virgili R, Piredda M, et al. [The role of nurses in health education about vaccines: analysis of style and communication models of institutional vaccination campaigns]. Ig Sanita Pubbl [Internet]. [cited 2020 Mar 10];75(5):355–69. Available from: http://www.ncbi.nlm.nih.gov/pubmed/31971520

6. Gil Cuesta J, Whitehouse K, Kaba S, Nanan-N'Zeth K, Haba B, Bachy C, et al. 'When you welcome well, you vaccinate well': a qualitative study on improving vaccination coverage in urban settings in Conakry, Republic of Guinea. Int Health.

7. Smith LE, Webster RK, Weinman J, Amlôt R, Yiend J, Rubin GJ. Psychological factors associated with uptake of the childhood influenza vaccine and perception of post-vaccination side-effects: A cross-sectional survey in England. Vaccine. 2017 Apr;35(15):1936–45.

8. Owais A, Hanif B, Siddiqui AR, Agha A, Zaidi AK. Does improving maternal knowledge of vaccines impact infant immunization rates? A community-based randomized-controlled trial in Karachi, Pakistan. BMC Public Health. 2011 Dec;11(1):239.

9. Guzman-Holst A, DeAntonio R, Prado-Cohrs D, Juliao P. Barriers to vaccination in Latin America: A systematic literature review. Vol. 38, Vaccine. Elsevier Ltd; 2020. p. 470–81.

10. Damnjanović K, Graeber J, Ilić S, Lam WY, Lep Ž, Morales S, et al. Parental decision-making on childhood vaccination. Front Psychol. 2018 Jun 13;9(JUN).

11. Harrington PM, Woodman C, Shannon WF. Low immunisation uptake: Is the process the problem? J Epidemiol Community Health. 2000 May;54(5):394–400.

12. Forbes TA, McMinn A, Crawford N, Leask J, Danchin M. Vaccination uptake by vaccine-hesitant parents attending a specialist immunization clinic in Australia. Hum Vaccines Immunother. 2015 Jan;11(12):2895–903.

13. Castelnuovo B, Newell K, Manabe YC, Robertson G. Multi-Media Educational Tool Increases Knowledge of Clinical Trials in Uganda. J Clin Res Bioeth. 2014 Jan;5:165.

14. Tefera Y, Wagner A, Mekonen E, Carlson B, Boulton M. Predictors and Barriers to Full Vaccination among Children in Ethiopia. Vaccines [Internet]. 2018 Apr 10 [cited

For peer review only - http://bmjopen.bmj.com/site/about/guidelines.xhtml

2019 Dec 8];6(2):22. Available from: http://www.mdpi.com/2076-393X/6/2/22

15. Endale E, Tesfaye S. Rapid Qualitative Assessment of Maternal and Newborn Health Care (MNHC) Speaking Book in Two Districts in the Amhara Region, Ethiopia. 2012.

16. Afolabi MO, McGrath N, D'Alessandro U, Kampmann B, Imoukhuede EB, Ravinetto RM, et al. A multimedia consent tool for research participants in the Gambia: A randomized controlled trial. Bull World Health Organ. 2015;93(5):320-328A.

17. Dhai A, Etheredge H, Cleaton-Jones P. A pilot study evaluating an intervention designed to raise awareness of clinical trials among potential participants in the developing world. J Med Ethics. 2010 Apr;36(4):238–42.

18. Braun V, Clarke V. Using thematic analysis in psychology. Qual Res Psychol. 2006;3(2):77–101.

19. Bedeker A, Anderson D, Lose T, Mgwatyu Y, Luus R, Blignaut R, et al. Understanding biobanking: An assessment of the public engagement speaking book intervention Biobanking and Me. South African J Bioeth Law. 2019 Dec 17;12(2):87.

20. Oku A, Oyo-Ita A, Glenton C, Fretheim A, Ames H, Muloliwa A, et al. Perceptions and experiences of childhood vaccination communication strategies among caregivers and health workers in Nigeria: A qualitative study. PLoS One. 2017 Nov 1;12(11).

21. Masters NB, Tefera YA, Wagner AL, Boulton ML. Vaccine hesitancy among caregivers and association with childhood vaccination timeliness in Addis Ababa, Ethiopia. Hum Vaccines Immunother. 2018 Oct 3;14(10):2340–7.

22. Arogundade L, Akinwumi T, Molemodile S, Nwaononiwu E, Ezika J, Yau I, et al. Lessons from a training needs assessment to strengthen the capacity of routine immunization service providers in Nigeria. BMC Health Serv Res [Internet]. 2019 Sep 14 [cited 2020 Mar 10];19(1):664. Available from: https://bmchealthservres.biomedcentral.com/articles/10.1186/s12913-019-4514-2

23. Dubé E, Gagnon D, MacDonald NE, Eskola J, Liang X, Chaudhuri M, et al. Strategies intended to address vaccine hesitancy: Review of published reviews. Vaccine. 2015 Aug 14;33(34):4191–203.

24. World survey reveals people trust experts but want to know more about science | Wellcome [Internet]. [cited 2020 Feb 5]. Available from: https://wellcome.ac.uk/news/world-survey-reveals-people-trust-experts-want-know-more-about-science

25. WHO | Improving vaccination demand and addressing hesitancy. WHO. 2019;

For peer review only

For peer review only - http://bmjopen.bmj.com/site/about/guidelines.xhtml

FIGURE LEGENDS

Figure 1
Excerpts from the Speaking Book

Figure 2
Flow chart of participant recruitment

Figure 3
Themes and sub-themes from in-depth interviews with healthcare workers

For peer review only

For peer review only - http://bmjopen.bmj.com/site/about/guidelines.xhtml

Vaccines Save Children's Lives

(How Vaccines Protect Your Baby)

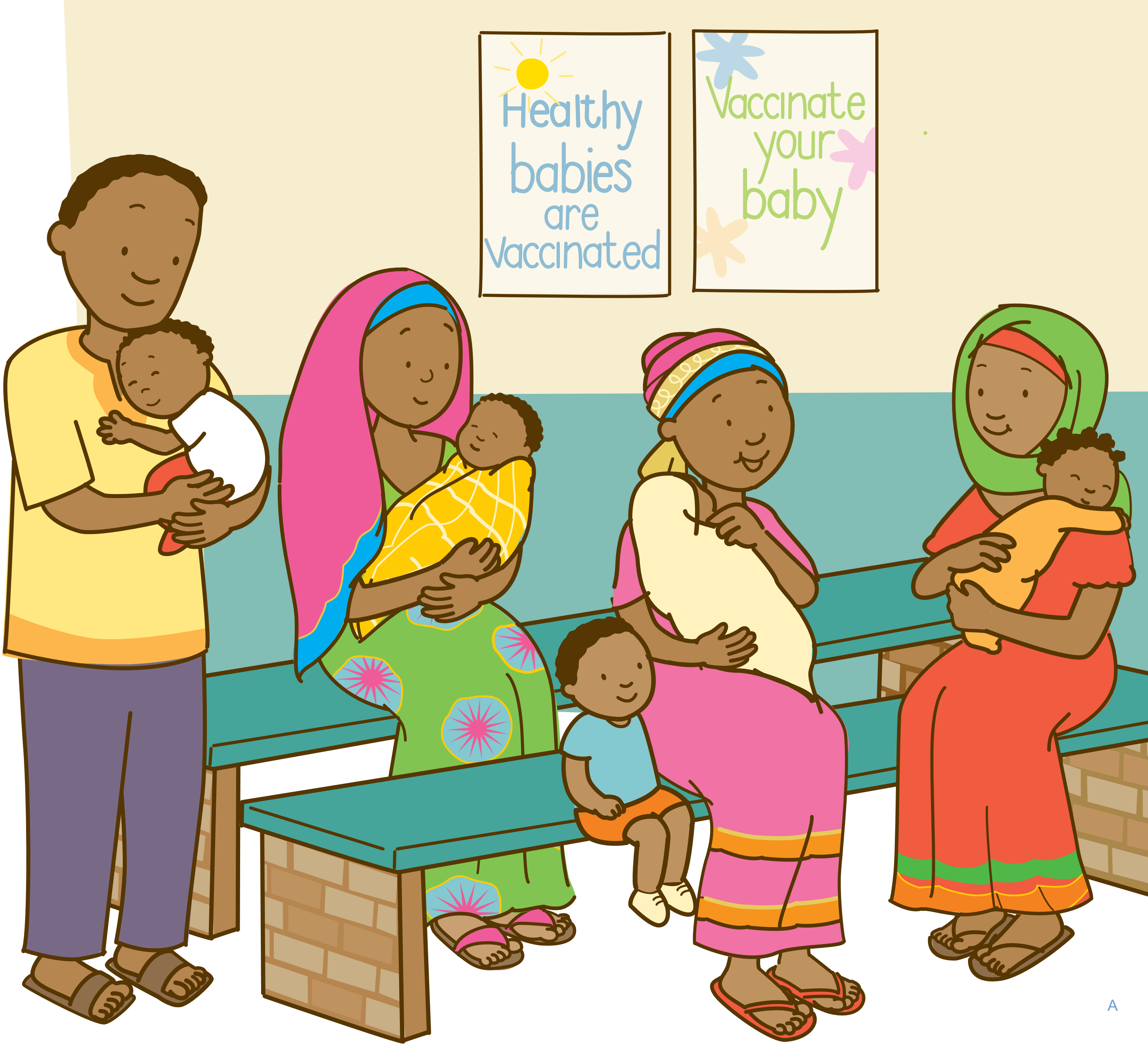

This book was developed by the MRC Unit The Gambia at the London School of Hygiene and Tropical Medicine in collaboration with The Gambia Expanded Programme on Immunization

TEXT: ENGLISH AUDIO: WOLOF AND MANDINKA

VACCINES SAVE CHILDREN'S LIVES (HOW VACCINES PROTECT YOUR BABY)

ENGLISH, WOLOF AND MANDINKA

Vaccines Save Children's Lives

(How Vaccines Protect Your Baby)

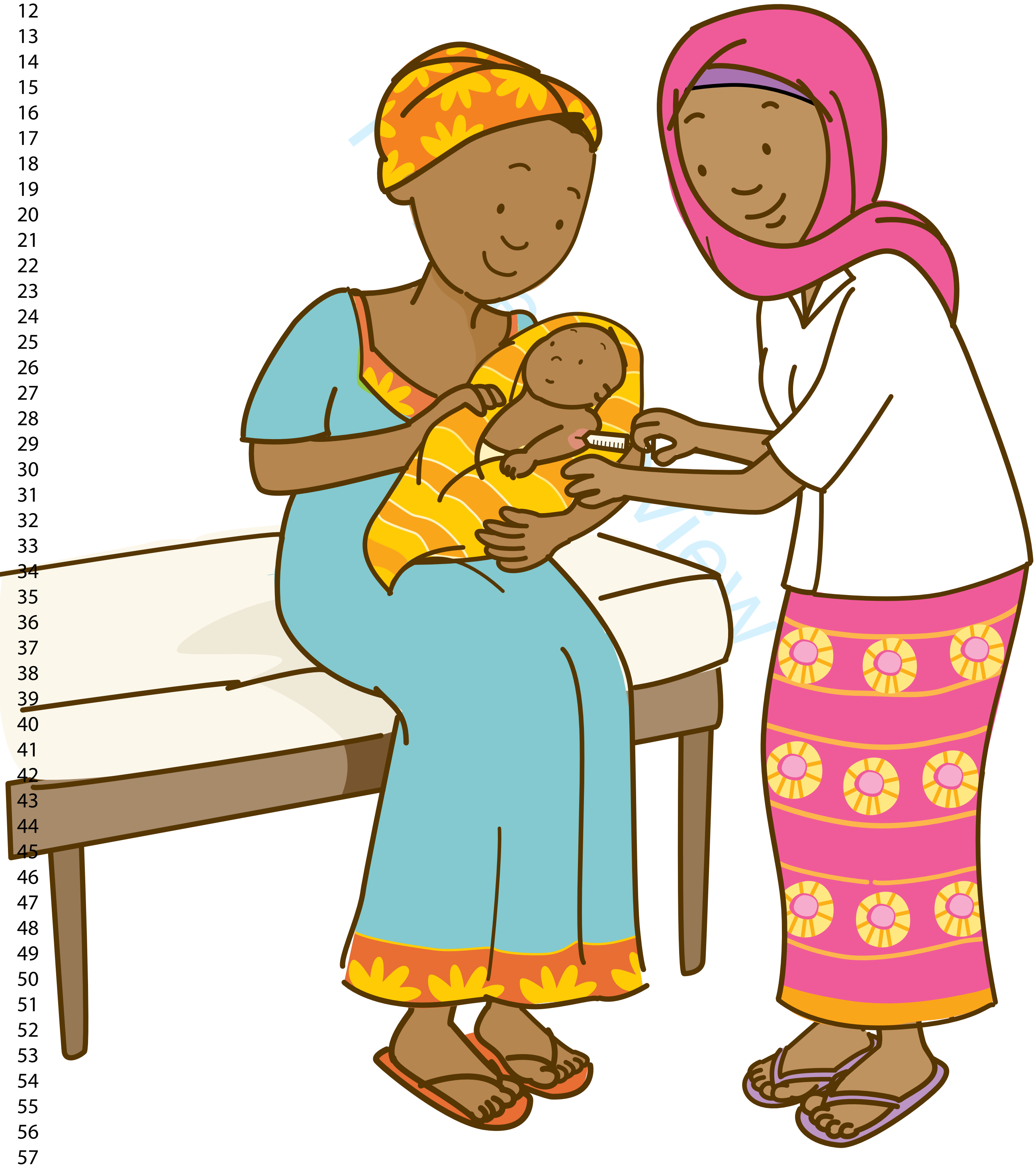

Press the buttons to hear about how
Vaccines Save Children's Lives

For peer review only - http://bmjopen.bmj.com/site/about/guidelines.xhtml

Does my child need to receive all vaccines recommended by the government?

Your baby needs to receive all vaccines given by the government. This is to make sure they're protected and can't get sick from infections prevented by the vaccines. The vaccines are free in government health facilities. Always take your Infant Welfare Card for the health workers to check. They'll tell you which vaccines your baby has received. They'll also tell you when you need to bring your baby back.

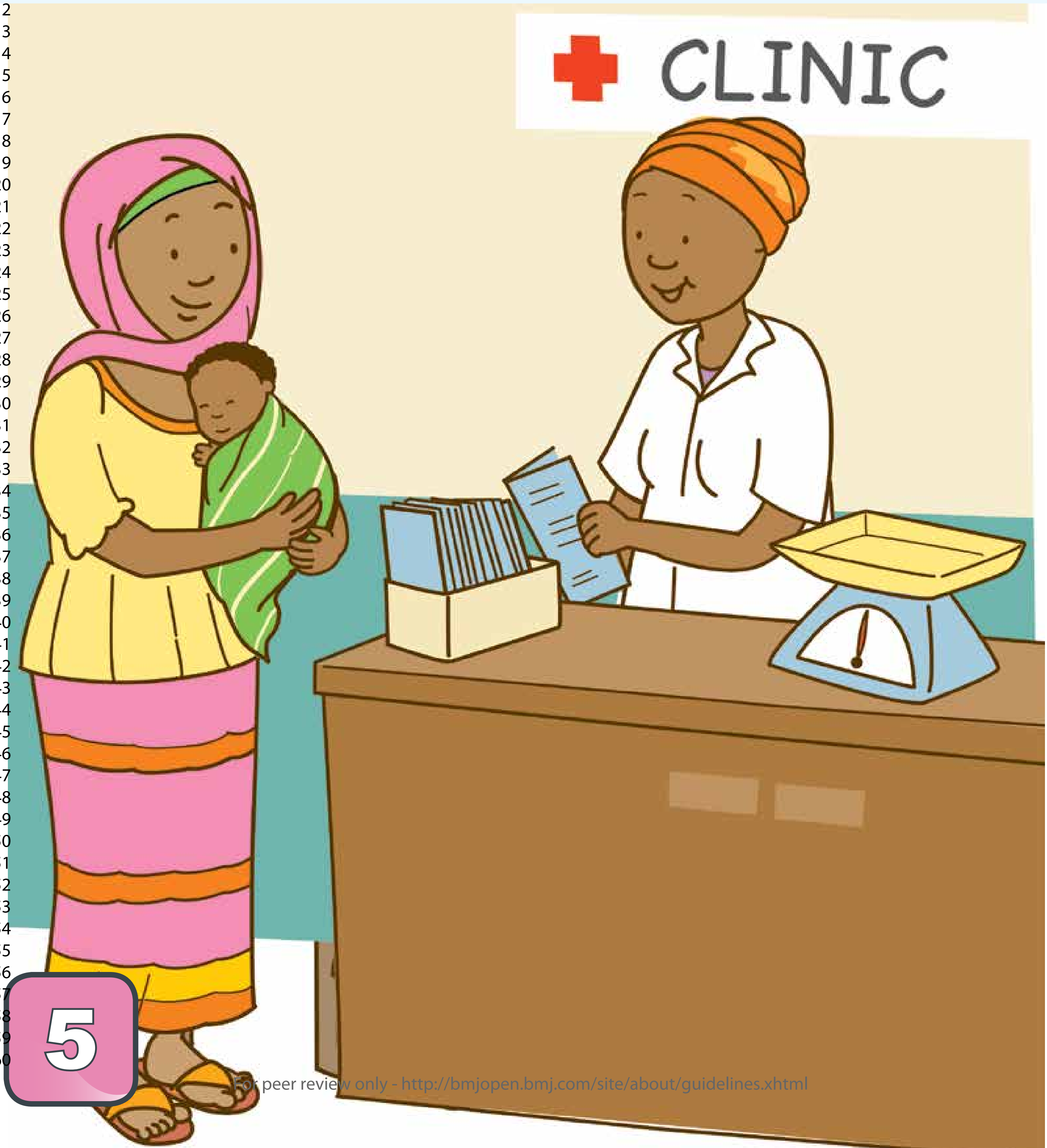

For peer review only - http://bmjopen.bmj.com/site/about/guidelines.xhtml

Is it important that my child receives all vaccines and on time?

It's very important that your baby receives their vaccines on time - at birth, 2 months, 3 months, 4 months, 9 months and 18 months. Vaccines protect babies early in life when they're most at risk of infection and are exposed to many illnesses and diseases. When your baby has not received all the required vaccines or the vaccines are late, they aren't protected.

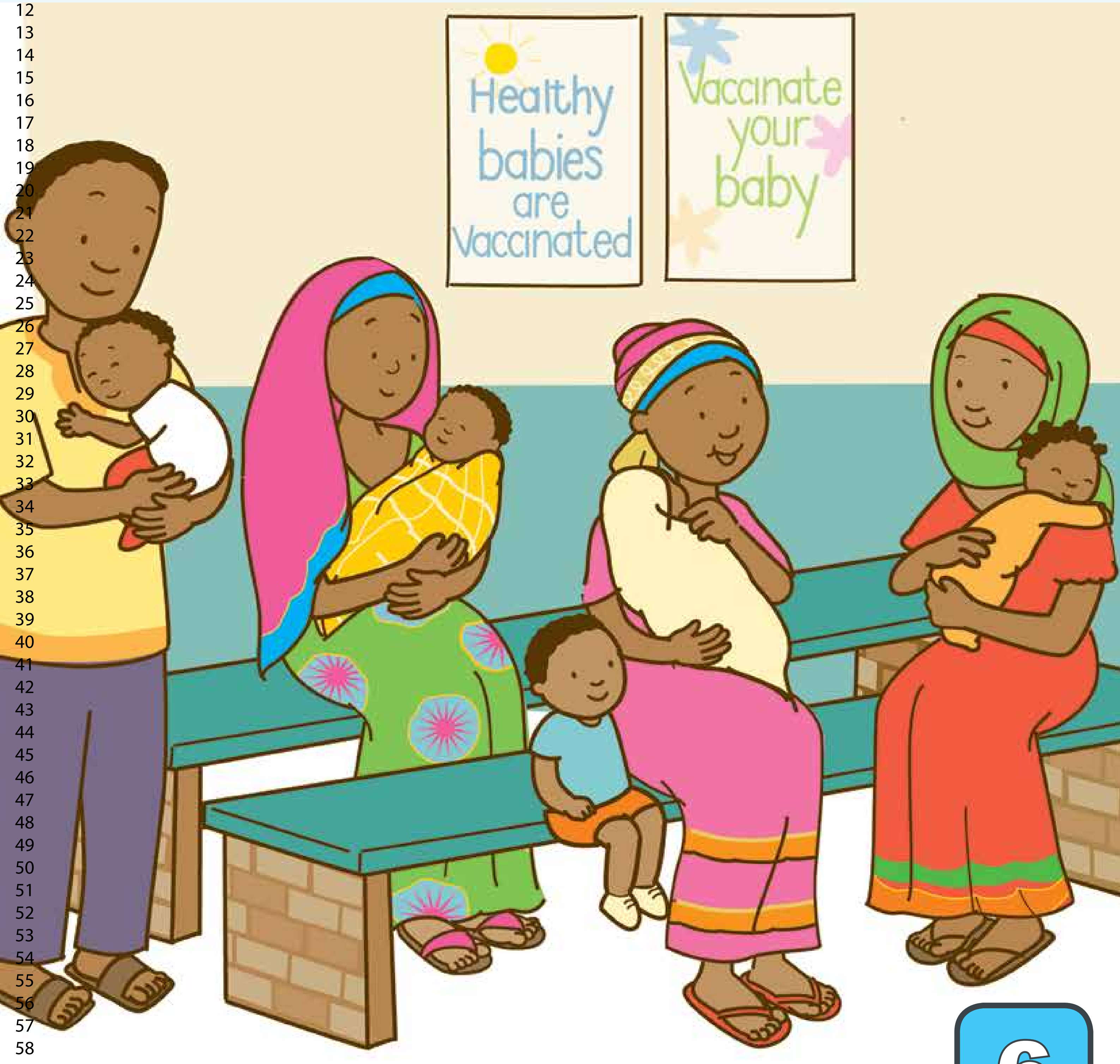

6

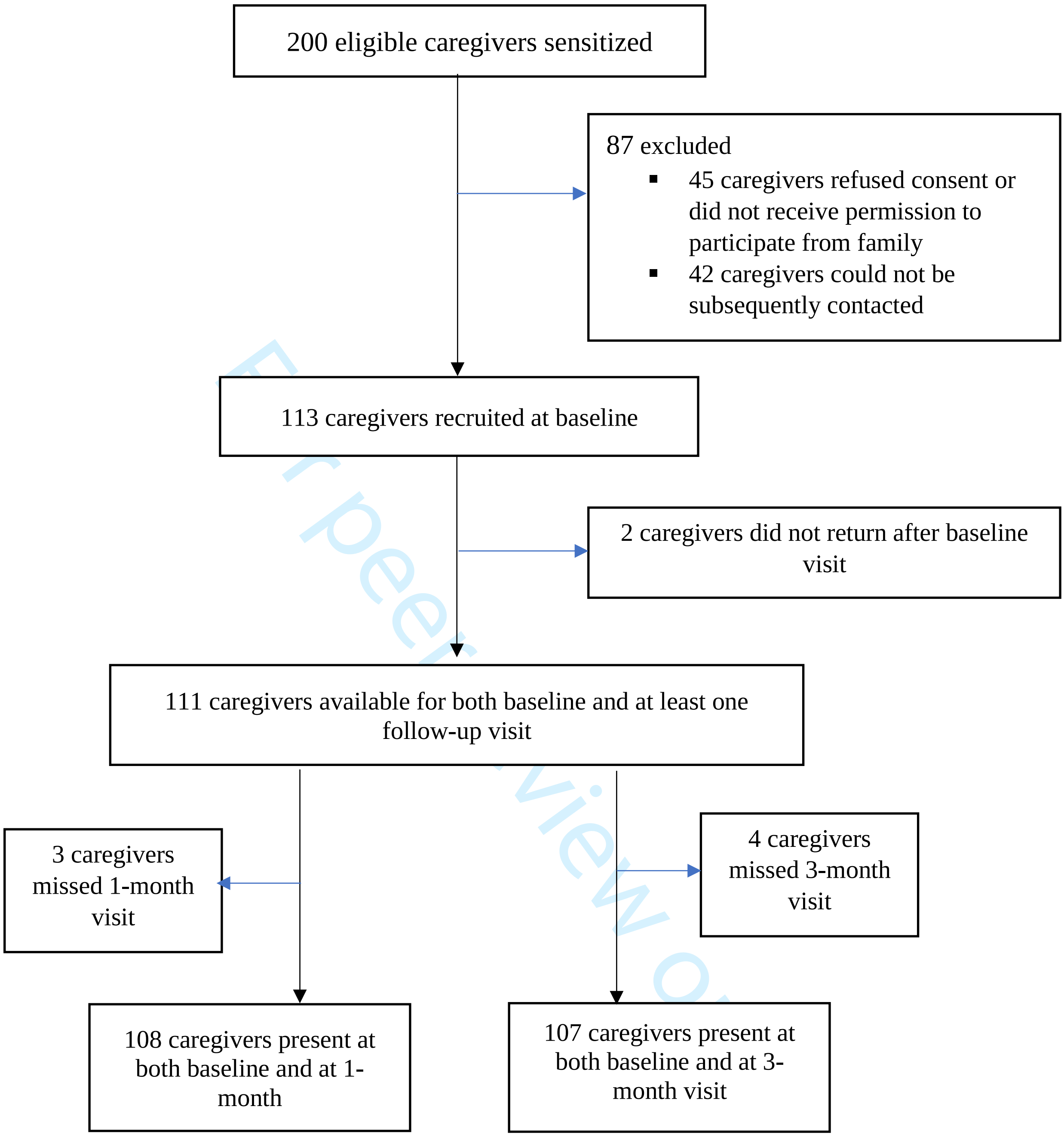

For peer review only - http://bmjopen.bmj.com/site/about/guidelines.xhtml

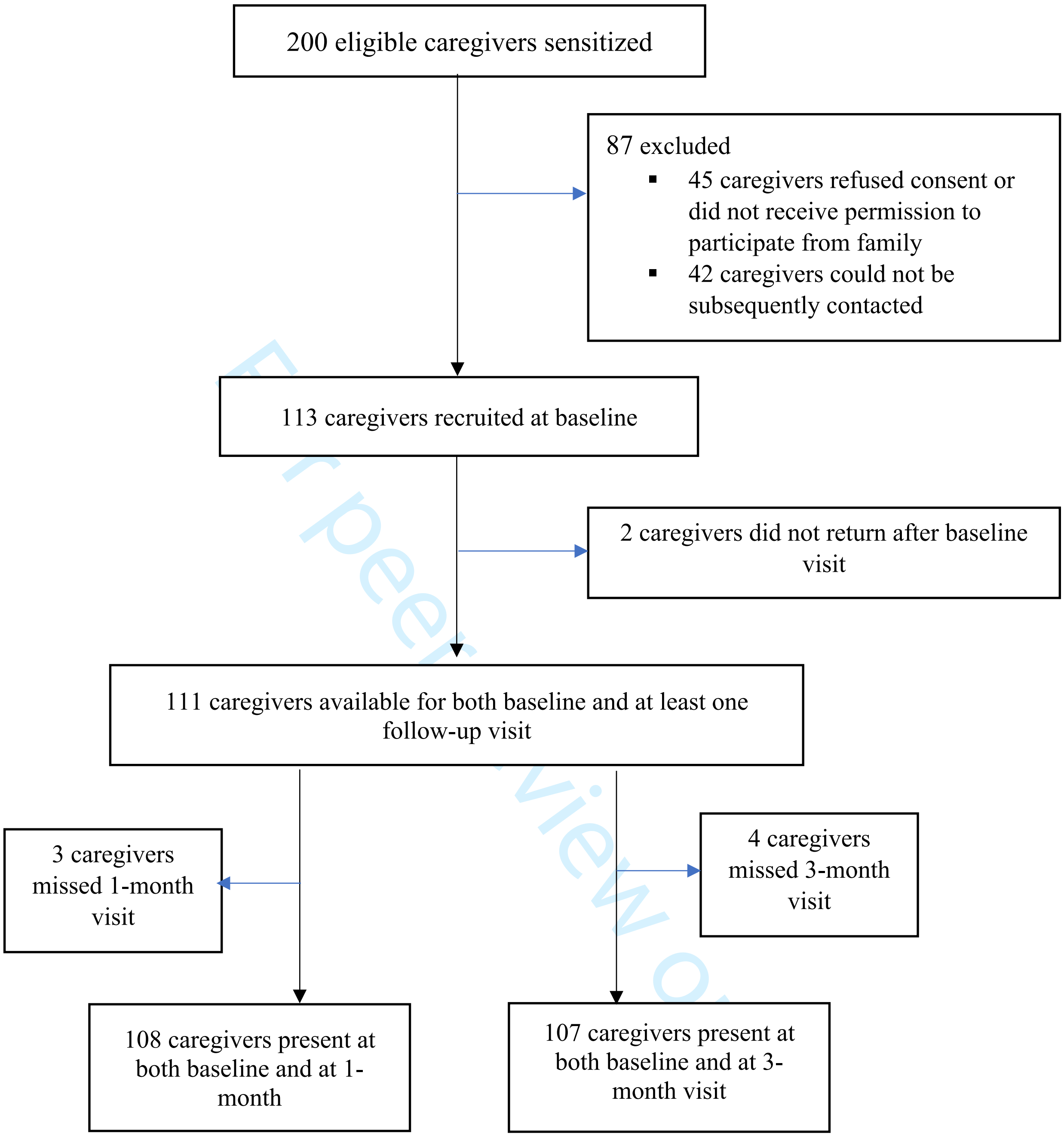

For peer review only - http://bmjopen.bmj.com/site/about/guidelines.xhtml

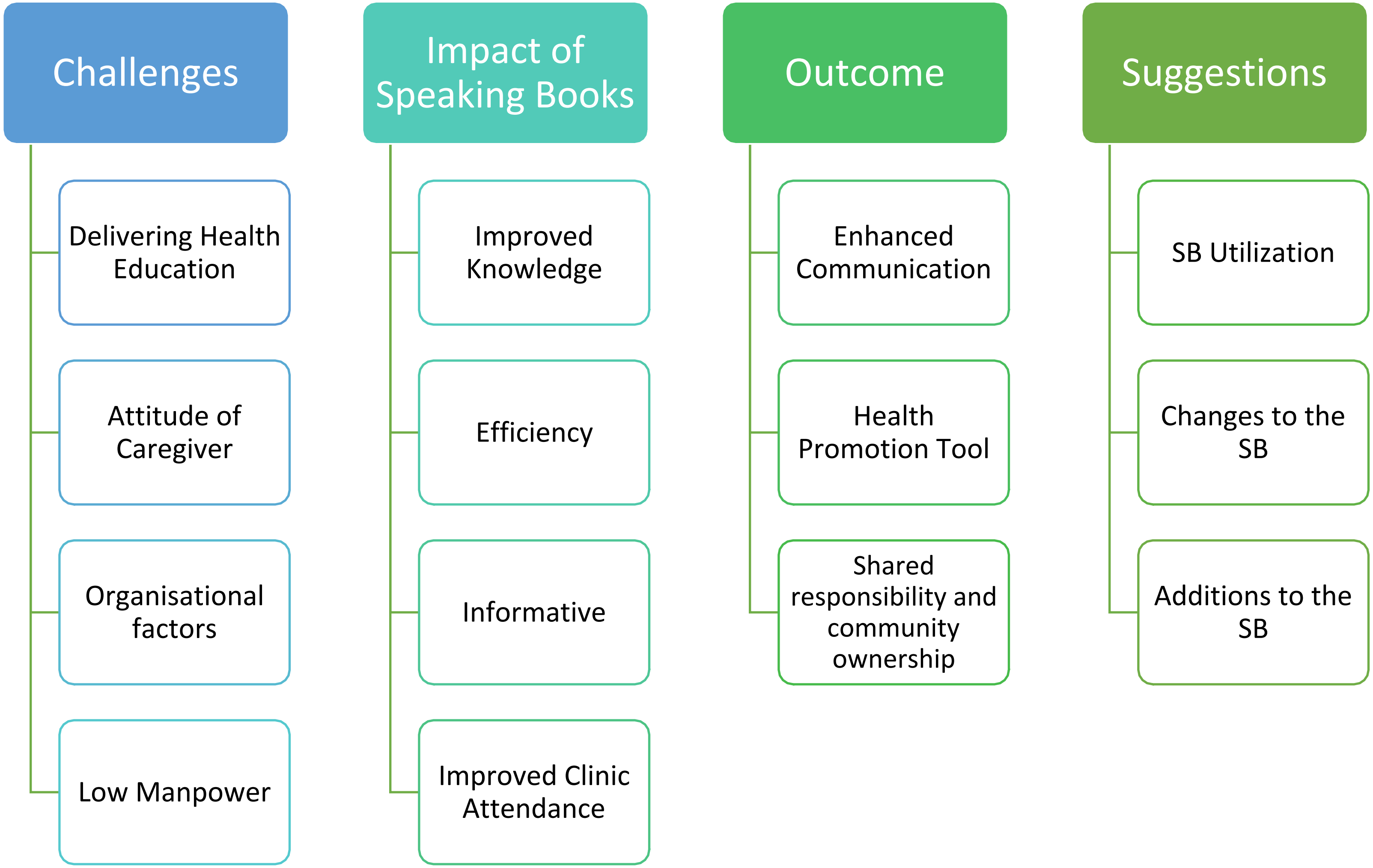
Challenges
Delivering Health Education
Attitude of Caregiver
Organisational factors
Low Manpower
Impact of Speaking Books
Improved Knowledge
Efficiency
Informative
Improved Clinic Attendance
Outcome
Enhanced Communication
Health Promotion Tool
Shared responsibility and community ownership
Suggestions
SB Utilization
Changes to the SB
Additions to the SB

BMJ Open

A SEQUENTIAL MIXED-METHODS STUDY ON THE USE OF *A SPEAKING BOOK®* TO ENHANCE VACCINE KNOWLEDGE AMONG CAREGIVERS IN THE GAMBIA

Journal:	*BMJ Open*
Manuscript ID	bmjopen-2020-040507.R1
Article Type:	Original research
Date Submitted by the Author:	02-Nov-2020

Complete List of Authors:	Nkereuwem, Oluwatosin; Medical Research Council Unit The Gambia at the London School of Hygiene and Tropical Medicine, Vaccines and Immunity Theme<br>Kochhar, Sonali; Global Healthcare Consulting, New Delhi, India., Global Health; University of Washington Department of Global Health, Global Health<br>Wariri, Oghenebrume; Medical Research Council Unit The Gambia at the London School of Hygiene and Tropical Medicine, Vaccines and Immunity Theme<br>Johm, Penda; Medical Research Council Unit The Gambia at the London School of Hygiene and Tropical Medicine, Vaccines and Immunity Theme<br>Ceesay, Amie; Medical Research Council Unit The Gambia at the London School of Hygiene and Tropical Medicine, Vaccines and Immunity Theme<br>Kinteh, Mamanding; Expanded Programme on Immunization, Ministry of Health and Social Welfare, The Gambia<br>Kampmann, Beate ; Medical Research Council Unit The Gambia at the London School of Hygiene and Tropical Medicine, Vaccines and Immunity ; London School of Hygiene and Tropical Medicine Faculty of Infectious and Tropical Diseases, The Vaccine Centre

**Primary Subject Heading**:	Epidemiology
Secondary Subject Heading:	Public health, Qualitative research, Global health, Communication
Keywords:	Public health < INFECTIOUS DISEASES, PRIMARY CARE, QUALITATIVE RESEARCH, PREVENTIVE MEDICINE

SCHOLARONE™
Manuscripts

I, the Submitting Author has the right to grant and does grant on behalf of all authors of the Work (as defined in the below author licence), an exclusive licence and/or a non-exclusive licence for contributions from authors who are: i) UK Crown employees; ii) where BMJ has agreed a CC-BY licence shall apply, and/or iii) in accordance with the terms applicable for US Federal Government officers or employees acting as part of their official duties; on a worldwide, perpetual, irrevocable, royalty-free basis to BMJ Publishing Group Ltd ("BMJ") its licensees and where the relevant Journal is co-owned by BMJ to the co-owners of the Journal, to publish the Work in this journal and any other BMJ products and to exploit all rights, as set out in our licence.

The Submitting Author accepts and understands that any supply made under these terms is made by BMJ to the Submitting Author unless you are acting as an employee on behalf of your employer or a postgraduate student of an affiliated institution which is paying any applicable article publishing charge ("APC") for Open Access articles. Where the Submitting Author wishes to make the Work available on an Open Access basis (and intends to pay the relevant APC), the terms of reuse of such Open Access shall be governed by a Creative Commons licence – details of these licences and which Creative Commons licence will apply to this Work are set out in our licence referred to above.

Other than as permitted in any relevant BMJ Author's Self Archiving Policies, I confirm this Work has not been accepted for publication elsewhere, is not being considered for publication elsewhere and does not duplicate material already published. I confirm all authors consent to publication of this Work and authorise the granting of this licence.

For peer review only

A SEQUENTIAL MIXED-METHODS STUDY ON THE USE OF A *SPEAKING BOOK*® TO ENHANCE VACCINE KNOWLEDGE AMONG CAREGIVERS IN THE GAMBIA

Oluwatosin Nkereuwem[1], Sonali Kochhar[2,3], Oghenebrume Wariri[1], Penda Johm[1,5], Amie Ceesay[1], Mamanding Kinteh[4], Beate Kampmann[1,5]

[1] Vaccines & Immunity Theme, MRC Unit the Gambia at The London School of Hygiene and Tropical Medicine, The Gambia

[2] Global Healthcare Consulting, New Delhi, India

[3] Department of Global Health, University of Washington, Seattle, USA

[4] Expanded Programme on Immunization, Ministry of Health and Social Welfare, The Gambia

[5] The Vaccine Centre, Faculty of Infectious Diseases, London School of Hygiene and Tropical Medicine, Keppel Street, London, UK

Correspondence to:

Prof. Beate Kampmann, Vaccines and Immunity Theme, Medical Research Council Unit The Gambia at London School of Hygiene and Tropical Medicine, Fajara, P.O. Box 273, Banjul, The Gambia.

Email: bkampmann@mrc.gm

For peer review only

Keywords

Vaccine knowledge; Caregiver knowledge; Communication tools; Multi-media educational tool; Expanded Programme on Immunization (EPI)

ABSTRACT

Objectives

To measure the usefulness of a *Speaking Book*® as an educational tool for enhancing knowledge, understanding and recall of key vaccine-related information among caregivers in The Gambia, as well as its acceptability and relevance as a health promotion tool for caregivers and healthcare workers.

Design and setting

We developed a multi-media educational tool, the vaccine *Speaking Book*®, which contained pre-recorded information about vaccines provided in The Gambia's Expanded Programme on Immunization (EPI). We then conducted a sequential mixed-methods study on the use of this tool among caregivers and healthcare workers in The Gambia.

Participants

200 caregivers attending primary health care centres in The Gambia for routine immunization services for their infants, and 15 healthcare workers employed to provide immunization services at these clinics.

Outcome measures

We calculated the median knowledge scores on vaccine-related information obtained at baseline, 1-month 3-month follow-up visits. Wilcoxon's matched-pairs signed-rank test was used to compare the difference in the median knowledge scores between baseline and 1-month, and between baseline and 3-month follow-up visits.

Results

Of the 113 caregivers who participated, 104 (92%) completed all three study visits, 108 (95.6%) completed the baseline and 1-month follow-up visits, and 107 (94.7%) completed the baseline and 3-month follow-up visits. The median knowledge score increased from 6.0 (IQR 5.0-7.0) at baseline to 11.0 (IQR 8.0-14.0) at 1-month visit ($P < 0.001$), and 15.0 (IQR 10.0 – 20.0) at 3-month visit ($P < 0.001$). Qualitative results showed high acceptability and enthusiasm for the *Speaking Book*® among both caregivers and healthcare workers. The *Speaking Book*® was widely shared in the community and this facilitated communication with healthcare workers at the primary healthcare centres.

Conclusions

Context and subject specific *Speaking Books*® are a useful communication and educational tool to increase caregiver vaccine knowledge in low-and-middle-income countries.

For peer review only - http://bmjopen.bmj.com/site/about/guidelines.xhtml

Strengths and limitations of this study

- The vaccines *Speaking Book*® (SB), a richly illustrated, context-specific, audio-visual, educational tool was designed to complement vaccine information given to caregivers by health care workers in immunization clinics in The Gambia.
- A sequential mixed methods approach was used to assess the impact of the (*SB*) as a health promotion tool for use by caregivers and healthcare workers.
- There is a possibility of self-selection bias due to the absence of a control group.
- Much further research is needed to quantify any potential effect on vaccine uptake at the health center level and measure cost-effectiveness.

For peer review only

INTRODUCTION

Immunization is one of the most effective public health interventions for the control and prevention of infectious diseases.[1] Unfortunately, global immunization coverage rates remain sub-optimal with approximately 21.8 million infants never completing the recommended immunization schedule.[2, 3]

Several factors may act as barriers to childhood immunization especially in low-and-middle income countries (LMIC) where caregivers often have low literacy levels.[4-7] In addition to the wider concerns of vaccine confidence that have been expressed across the globe, more specific issues include caregivers lack of knowledge about the recommended immunization schedules and alternatives once their infants miss scheduled immunizations, concerns about vaccine safety and adverse events, infants being unwell at the time of the appointment, reluctance to receive multiple vaccinations at the same time, lack of trust in the medical community, being unable to remember the information provided and the date for the next immunisation visit, hesitancy in asking questions which might be perceived as trivial by healthcare workers (HCW), and HCW being too busy or lacking the knowledge or patience to answer the caregivers questions and addressing their concerns.[4, 8-10]

The caregivers' knowledge and overall vaccine literacy influence their decision to access immunization services for their infants.[8, 11] This decision in turn impacts on the uptake of vaccination services, and consequently, on the morbidity and mortality attributable to vaccine-preventable diseases (VPD).[8, 9] Data from high-income countries (HIC) have shown that improving health awareness and knowledge of caregivers about disease prevention can improve health outcomes, especially among less literate populations.[12] This would be applicable especially in LMIC in sub-Saharan Africa where caregivers have been shown to have low knowledge about immunization and vaccine-related issues.[13]

The Gambian expanded programme on immunization (EPI) programme is considered as highly successful compared to other countries in sub-Saharan Africa. The programme has consistently maintained coverage of the third dose of Diphtheria-Tetanus-Pertussis (DTP3) above 95% and DTP1 to DTP3 dropout rates below 10% since 2005. However, despite these overall high coverages documented over the years, there still exist wide intra-country equity gaps along the lines of caregivers' education and wealth quintile.[14]

Recently, audio-visual tools like the *Speaking Book*® (*SB*) have been developed and used to target HCW and care-seekers in human immunodeficiency virus (HIV) care and mental health services, with studies demonstrating their usefulness in the improvement of knowledge.[12, 15, 16] These studies have also shown that *SB* complements communication between HCW and health care seekers. In a previous study conducted in The Gambia, a clinical trials-specific *SB* was shown to increase the knowledge of study participants, leading to a better informed decision about participation.[12] Therefore, using context-specific, pictorial depictions followed by recorded audio messages targeted at caregivers is

For peer review only - http://bmjopen.bmj.com/site/about/guidelines.xhtml

considered an effective way to deliver messages about vaccines in general, especially in a low literacy setting such as The Gambia.[17]

In this study, we developed the vaccine *SB*, a new *SB* covering routinely recommended maternal and childhood vaccines. We assessed its use as an educational tool for enhancing knowledge, understanding and recall of key vaccine-related information among caregivers in The Gambia, as well as its acceptability and relevance as a health promotion tool for caregivers and HCW.

METHODS

Study design and setting

We conducted a sequential exploratory mixed-methods study which enrolled caregivers and their infants attending immunization clinics in 15 purposively selected Primary Health Care facilities (PHCs) across four regions of The Gambia. The PHCs comprised of seven rural and eight urban centres that had not previously participated in vaccine-related research studies or clinical trials. Based on existing records, the Gambia EPI selected immunization facilities with poorer performance compared to expected national outcomes to participate in the study. The project was conducted in close collaboration with the communications department of the Gambia EPI.

Immunization services are provided free-of-charge at all government facilities in The Gambia, and caregivers are encouraged to access the services closest to their home. Immunization-related information should be routinely delivered during immunization clinic days by public health officers (PHO). For the purpose of this study, we defined a caregiver as an adult aged 18 years and above attending the immunisation clinic with an infant, and responsible for that infant's day-to-day care. This included but was not limited to biological parents.

Ethical approval was obtained from The Gambia Government/MRC Joint Ethics Committee (SCC 1598).

Study tool

The prototype of the richly illustrated, audio-visual educational tool called a *Speaking Book* (*SB*) was developed by a United States-based company (https://speakingbooks.com/), with whom we collaborated. The *SB* concept can be adapted for specific purposes, and in our case, we adapted the text, recording and illustrations to vaccines routinely delivered by the Gambia EPI program.

During an iterative pilot phase, we developed a bespoke *SB* version and conducted four separate focus group discussions (FGD) with Gambia EPI programme managers, HCW, and caregivers attending EPI clinics to ensure that the final version of the *SB* reflected the local Gambian context. The final version of the *SB* was an A4-size hard cover book consisting of 16 pages of colourful, culturally sensitive illustrations with short texts written in English and recorded narrations in the two most widely spoken local languages, *Wolof* and *Mandinka.* Each *SB* has a plastic panel with removable

For peer review only - http://bmjopen.bmj.com/site/about/guidelines.xhtml

battery, which hosts a series of push buttons, each corresponding to a specific page in the *SB*. When activated, the push buttons trigger a soundtrack of the text on the relevant page. The soundtrack was narrated by two respected local actors, with the appropriate voice and tonal quality. The language could be selected via a switch button. Figure 1 shows some photographs of the *SB*. (See supplementary material 1)

Data collection methods

Quantitative methods

Following a wide literature search, we designed a structured questionnaire for the quantitative data collection. To assess the construct and content of the data collection tool, we pre-tested it with 25 caregivers and five HCW who provided feedback which was used to refine the wording of the questions and response options. The final version of the questionnaire consisted of five sections capturing the following: i) socio-demographics of the caregiver (19 questions); ii) immunization history and experiences of the caregiver (five questions); iii)socio-demographics of their child(ren) (10 questions); iv) a series of 8 multiple-choice questions with 39 correct choices assessing vaccines and immunization knowledge of the caregiver; and v) a series of 18 open and close-ended questions assessing the experiences of the caregiver on the use of the *SB*. A sample questionnaire is included in supplementary materials 2 and 3.

Qualitative methods

A qualitative approach was used to explore the perception of HCW on the use of the *SB* as a health promotion tool when sharing vaccine information with caregivers during immunisation clinics. We carried out in-depth interviews (IDI) with HCW using 11 general, open-ended and non-leading questions. These included questions which explored the challenges they faced when sharing vaccine information with caregivers, impact of the *SB* tool on their work, outcomes since the utilization of the *SB*, and suggestions on the future use of the tool. The interview guide is in the supplementary material 4.

Participant selection and data collection

All study participants were sensitized on the study procedures and objectives. Participants were eligible for inclusion if they were caregivers with infants below six months of age attending one of the selected PHC centres in The Gambia, had been living at their current address for at least six months, were able to communicate in either *Wolof* or *Mandinka,* and were able to provide written consent. Purposive sampling strategy was used to select caregivers who met the inclusion criteria for enrolment into the study.

During an initial sensitization visit, the research staff educated the caregivers on the aim of the study and gave the caregivers an information sheet and consent form to take home and return on another date. Each caregiver who returned a completed consent form was then invited to participate in a

For peer review only - http://bmjopen.bmj.com/site/about/guidelines.xhtml

baseline visit during which the questionnaire was interviewer-administered to obtain socio-demographic information and to assess their baseline knowledge about childhood and maternal vaccines. Only one attempt was allowed for response to the questions. To assess the knowledge of caregivers, we computed a knowledge score which was calculated by assigning a score of 1 for each correct answer and 0 for each incorrect answer, with a maximum and minimum knowledge score of 39 and 0 respectively. Only sections i) to iv) of the questionnaire were administered during this baseline visit.

Subsequently, the mechanics of the *SB* were explained, and each caregiver was given a copy of the *SB* for use at home. Each PHC head was also given a copy of the *SB* to be used during health education activities in the facilities. Caregivers could listen to the *SB* as many times as they wished during the entire study period and were also encouraged to invite other people to listen along with them. Following receipt of the *SB,* the participants were requested to return to the PHCs for follow-up visits at one month and three months after the baseline visit. During each of these follow-up visit, the participants' understanding, retention, utilisation of key information and experiences using the *SB* were assessed with the same questionnaire used at baseline. Section v) of the questionnaire was also administered during this visit.

At the end of the three months, we conducted in-depth interviews with one representative HCW from each of the participating immunization clinics. During this interview, we assessed their perception of the acceptability, potential efficacy and use of the *SB* as a health promotion tool for HCW delivering immunisation services in The Gambia. Qualitative data were audio-recorded and transcribed by the research team.

Data analysis

The median knowledge scores were calculated at the baseline, 1-month and 3-month follow-up visits. We compared the difference in the median knowledge scores between baseline and one month, and between baseline and three months for the entire cohort and by sub-groups using the Wilcoxon's matched-pairs signed-rank test for non-parametric paired data. The sub-groups used for analysis included region, age, household income and level of education of caregiver. The distribution of the scores are presented in tables showing median scores and inter-quartile ranges (IQR) as appropriate, with significance level set at $P < 0.05$. All quantitative data were entered into Research Data Capture (REDCap) for proper documentation and analyses were performed using Stata version 13 (StataCorp, USA).

For the qualitative analysis, we applied inductive thematic analysis in deriving our themes and sub-themes as described by Braun et al.[18] We used NVivo® (version 12) software to organise the qualitative data during the analysis.

For peer review only - http://bmjopen.bmj.com/site/about/guidelines.xhtml

Patient and public involvement

The public (caregivers and HCW) were involved in the design of the *SB* and the questionnaires used for data collection tool.

RESULTS

Between January and July 2019, we approached 200 eligible caregivers to participate in the study of which a total of 113 were enrolled after exclusion of 45 caregivers due to refusal of consent or absence of permission to participate from family, and 42 caregivers who could not be subsequently contacted on the phone numbers and addresses provided (Figure 2). Of the 113 caregivers, 104 (92%) completed all 3 study visits, 108 (95.6%) completed the baseline and 1-month follow up visits, and 107 (94.7%) completed the baseline and 3-month follow up visits. Reasons for missed follow-up visits were ill-health of the mother, ill-health of infant and change in location.

All consenting caregivers were biological mothers of the infants presenting at the health facility. The mean age of the caregivers was 26.7 years ± 5.95. Most of the caregivers were married (n= 106, 93.8%), Muslim (n= 111, 98.2%), and unemployed (n=91, 80.5%). Over two-thirds of the caregivers reported that they lived within one hour of the immunization clinic, and most women (n=109, 96.5%) had received at least one dose of tetanus toxoid (TT) during their last pregnancy. Although none of the caregivers identified the media as their source of information regarding immunization, 90 (79.6%) households had a functional radio, and about half of them had a television in their homes. Over two-thirds of the caregivers acknowledged that HCW at the immunization clinics were their major source of information on immunization. Table 1 summarises the socio-demographic characteristics of the caregivers.

Table 1: Sociodemographic characteristics of Caregivers enrolled

**Variable**	**N = 113**	**%**
**Age (years)**		
<20	23	20.4
21-30	64	56.6
>30	26	23.0
**Religion**		
Muslim	111	98.2
Christian	2	1.8
**Region**		
Urban	52	46.0
Rural	61	54.0
Marital status		
Unmarried	7	6.2
Married	106	93.8
**Employment status**		

For peer review only - http://bmjopen.bmj.com/site/about/guidelines.xhtml

Employed	22	19.5
Unemployed	91	80.5
**Highest level of education**		
No Education	42	37.2
Primary	11	9.7
Secondary	21	18.6
Higher Secondary	21	18.6
Tertiary	2	1.8
Arabic/French	16	14.2
**Monthly household income**		
Below $50	19	16.8
$50-$100	38	33.6
$100-$200	14	12.4
Above $200	3	2.7
Don't Know	39	34.5
**Presence of working radio in the home**		
Yes	90	79.6
No	23	20.4
**Presence of working television in the home**		
Yes	56	49.6
No	57	50.4
**Reported distance to health facility**		
Less than 30 minutes	41	36.3
30 minutes to one hour	37	32.7
Over 1 hour	13	11.5
Not sure	22	19.5
**Mother's receipt of tetanus toxoid**		
Yes	109	96.5
No	4	3.5
**Usual source of information regarding immunization**		
Health workers	77	68.1
Infant welfare card	6	5.3
Family member or friend	4	3.5
Media	0	0.0
Not sure	26	23.0

Impact of *SB* on knowledge of caregivers

There was a significant increase in the median knowledge score from 6.0 (IQR 5.0-7.0) at baseline to 11.0 (IQR 8.0-14.0) at 1-month visit (P = <0.001), and 15.0 (IQR 10.0-20.0) at 3-month visit (P = <0.001). The highest median knowledge scores at 1-month and 3-month visits were seen among the caregivers above 30 years of age and families with monthly house-hold income above $100 (13.0 and 17.0 respectively). When analysed by subgroups (urban or rural, age of caregivers, household income or highest level of education), there was significant improvement in knowledge scores across all groups, as summarised in Table 2

For peer review only - http://bmjopen.bmj.com/site/about/guidelines.xhtml
For peer review only

Table 2: Median (IQR) of the knowledge scores at baseline, 1-month and 3-month visits*

Variable	N=108	Median baseline knowledge score[a]	Median knowledge score at 1-month[a]	Median difference[a] (1-month – baseline score)	*P*-value[b]	N=107	Median baseline knowledge score[c]	Median knowledge score at 3-months[c]	Median difference[c] (3-month – baseline score)	*P*-value[b]
**All caregivers**	108	6.0 (5.0-7.0)	11.0 (8.0-14.0)	5.0 (2.0-8.0)	<0.0001	107	6.0 (5.0-7.0)	15.0 (10.0-20.0)	8.0 (5.0-14.0)	<0.0001
**Region**										
Rural	57	6.0 (5.0-7.0)	11.0 (8.0-15.0)	4.0 (2.0-9.0)	<0.0001	58	6.0 (5.0-7.0)	15.5 (10.0-21.0)	10.0 (5.0-15.0)	<0.0001
Urban	51	6.0 (4.5-7.0)	11.0 (7.0-14.0)	5.0 (2.0-7.0)	<0.0001	49	6.0 (4.5-7.0)	13.0 (11.0-19.0)	8.0 (5.0-12.0)	<0.0001
**Age (years)**										
<20	23	6.0 (5.0-7.0)	9.0 (8.0-14.0)	4.0 (2.0-7.0)	0.0001	23	6.0 (5.0-7.0)	12.0 (8.0-19.0)	6.0 (2.0-14.0)	<0.0001
21-30	60	6.0 (5.0-7.0)	11.0 (7.0-14.0)	4.0 (2.0-8.0)	<0.0001	61	6.0 (5.0-7.0)	15.0 (11.0-21.0)	8.0 (5.0-14.0)	<0.0001
>30	25	6.0 (4.0-7.0)	13.0 (10.0-16.0)	6.0 (4.0-10.0)	<0.0001	23	6.0 (4.0-7.0)	16.0 (12.0-17.0)	9.0 (6.0-13.0)	<0.0001
**Household income**										
<$50	19	6.0 (5.0-7.0)	11.0 (10.0-14.0)	5.0 (4.0-8.0)	0.0002	18	6.0 (5.0-7.0)	15.5 (13.0-23.0)	9.5 (6.0-17.0)	0.0002
$50-100	38	6.0 (5.0-7.0)	11.0 (8.0-14.0)	5.0 (3.0-7.0)	<0.0001	37	6.0 (5.0-7.0)	14.0 (11.0-20.0)	8.0 (5.0-13.0)	<0.0001
>$100	15	6.0 (5.0-7.0)	12.0 (8.5-15.5)	7.0 (2.5-9.5)	0.0009	13	6.0 (5.0-7.0)	17.0 (14.0-20.0)	10.0 (7.0-13.0)	0.0004
Not sure	36	6.0 (5.0-6.0)	9.0 (7.0-15.0)	3.5 (1.5-9.5)	<0.0001	39	6.0 (5.0-6.0)	12.0 (8.0-20.0)	7.0 (3.0-15.0)	<0.0001
**Level of education**										
None	41	6.0 (5.0-7.0)	11.0 (8.0-15.0)	5.0 (2.0-10.0)	<0.0001	41	6.0 (5.0-7.0)	15.0 (11.0-21.0)	9.0 (5.0-15.0)	<0.0001
Primary	11	5.0 (4.0-6.0)	10.5 (8.0-15.0)	5.5 (4.0-9.0)	0.0057	11	5.0 (4.0-6.0)	16.5 (12.0-22.0)	12.0 (8.0-17.0)	0.005
Secondary	19	6.0 (5.0-7.0)	11.0 (9.0-12.0)	5.0 (3.0-7.0)	0.0001	19	6.0 (5.0-7.0)	13.0 (10.0-16.5)	7.5 (5.0-10.0)	0.0001
Higher sec./tertiary	22	6.0 (5.0-7.0)	10.5 (8.0-13.0)	4.5 (2.0-8.0)	0.0002	21	6.0 (5.0-7.0)	16.5 (10.0-23.0)	11.0 (5.0-16.0)	0.0001
Arabic/French	15	6.5 (6.0-7.0)	10.0 (7.0-15.0)	3.0 (1.5-8.5)	0.0048	15	6.0 (5.0-7.0)	13.0 (8.0-17.5)	6.0 (2.5-11.5)	0.0009

[a] *Median baseline knowledge score and median difference calculated using the same participants who were present at both baseline visit and 1-month follow-up visit*
[b] *Wilcoxon signed rank test P-values*
[c] *Median baseline knowledge score and median difference calculated using the same participants who were present at both baseline visit and 3-month follow-up visit*
* *Since median (IQR) of the differences are reported, these might not match the difference of the medians at each time point.*

For peer review only - http://bmjopen.bmj.com/site/about/guidelines.xhtml

Opinions of the caregivers

In general, the *SB* was very well received by the caregivers, and their opinions are summarised in Table 3. The caregivers found the *SB* increasingly easy to use 103/108 (95.4%) at 1 month, versus 107/107 (100%) at 3 months respectively), and most of them stated that they understood all the information in the book 100/108 (92.6%) at 1 month vs 98/107; 91.6% at 3 months. Many participants also shared the *SB* in their communities and expressed trust in the information provided. The survey results also showed that using the book was easy, with only 10 (9.3%) and 7 (6.5%) caregivers reporting some problems at 1-month and 3-month visits respectively.

Table 3: Opinions of caregivers about the *Speaking Book®*

Questions		1-month visit n=108	(%)	3-month visit n=107	(%)
Did you find the book easy to use?	Yes	103	95.4	107	100
	No	5	4.6	0	0
Did you like the pictures in the book	Yes	108	100	107	100
	No	0		0	0
Could you hear the person talking to you clearly?	Yes	108	100	107	100
	No	0		0	0
Did you understand all the information that you were told in the book	Yes	100	92.6	98	91.6
	No	8	7.4	9	8.4
Did you find the information in the book useful	Yes	107	99.1	106	99.1
	No	1	0.9	1	0.9
Did you trust the messages in the book?	Yes	108	100	106	99.1
	No	0	0	1	0.9
Did you learn new information from the book?	Yes	102	94.4	105	98.1
	No	6	5.6	2	1.9
How many times did you go through the whole book?	once	4	3.7	5	4.7
	2-5 times	40	37.0	33	30.8
	6-9 times	10	9.3	3	2.8
	≥10 times	54	50.0	66	61.7
Can you clearly explain the information in the book to your family and friends?	Yes	107	99.1	102	95.3
	No	1	1.0	5	4.7
Do you think that the information in the book gives you all the information needed to decide to immunize your child?	Yes	108	100	106	99.1
	No	0	0	1	0.9
Were there any problems you had when you were using the book?	Yes	10	9.3	7	6.5
	No	98	90.7	100	93.5
Do you think the book should include any other information or have any changes?	Yes	30	27.8	20	18.7
	No	78	72.2	87	81.3
Did you show the book to anyone in your Family?	Yes	107	99.1	106	99.1
	No	1	1.0	1	0.9
Did you show the book to anyone in your Community?	Yes	87	80.6	96	89.7
	No	17	15.7	11	10.3
	No Response	4	3.7	0	0.0
Did you show the book to anyone in your Mosque/Church?	Yes	4	3.7	2	1.9
	No	104	96.3	104	97.2
	No Response	0	0	1	0.9
Did you show the book to anyone at work?	Yes	21	19.4	16	15.0

For peer review only

	No	86	79.6	91	85.0
	No Response	1	0.9	0	0
Did you show the book to anyone at the clinic or hospital?	Yes	10	9.3	14	13.1
	No	95	88.0	90	84.1
	No Response	3	2.8	3	2.8
Do you think members of your family, mosque/church, and community will understand the information in the book if they were given this book to listen to?	Yes	106	98.1	100	93.5
	No	0	0.0	1	0.9
	I don't know	2	1.9	6	5.6
At what time do you think that the books should be given to a parent coming to get their child immunized?	At the time of first visit to the clinic only	82	75.9	102	95.3
	At first few visits to the clinic	11	10.2	5	4.7
	While speaking to the HCW	1	0.9	0	0
	Before speaking to the HCW	2	1.9	0	0
	After speaking to the HCW	10	9.3	0	0
	No Response	2	1.9	0	0
Do you know where the on/off switch is in the book?	Yes	108	100	107	100
	No	0	0.0	0	0
Would you be able to change the battery?	Yes	95	88.0	106	99.1
	No	11	10.2	1	0.9
	No Response	2	1.9	0	0
If you need to change the battery, do you have a screwdriver with which to do so?	Yes	83	76.9	97	90.7
	No	25	23.1	10	9.3

Themes from qualitative questions posed to Caregivers in questionnaire

In addition to the questions posed to caregivers on their experiences using the *SB* from the open-ended part of the questionnaire, a majority of caregivers (n=98, 90.7% and n=100, 93.5%) reported no problems while using the speaking books at 1- and 3-months post visit respectively. Reoccurring themes with supporting quotes are presented below:

- Information appropriateness

"I trust the messages in the book because the nurses tell me the same information as stated in the book." [32 years old, French language certificate, urban dweller]

- Clear presentation

"*... because the book is written in English... and also clearly translated in two local languages.*" [31 years old, Tertiary Education, urban dweller]

"*...the pictures make me believe the messages.*" [30 years old, No Education, rural dweller]

- Evidence of vaccine effectiveness

"*... I trust the messages because some diseases we used to see.... [giving examples] have now drastically reduce.*" [37 years old, Secondary (Grade 7–9), rural dweller]

For peer review only - http://bmjopen.bmj.com/site/about/guidelines.xhtml

For peer review only

Of the small number of caregivers who reported problems while using the speaking books at 1-month (n=10, 9.3%) and 3-month (n=7, 6.5%) follow-up visits respectively, the reoccurring themes with supporting quotes are presented below:

- Understanding information

"*... I found it difficult understanding the book at first, but after some time I began to understand better.*" [32 years old, Secondary (Grade 7–9), urban dweller]

"*... because I am not educated, I found the book difficult to learn despite the audio… I began to understand with the help of some students who read and explained to me.*" [25 years old, Secondary (Grade 7–9), urban dweller]

- Sense of responsibility

"... Initially I was afraid the book would go bad in my care but with time I was not afraid anymore." [27 years old, No education, rural dweller]

- Challenges with the batteries

"... The battery died and then I had to change it." [25 years old, Tertiary (University education), urban dweller]

Themes and sub-themes from in-depth interviews with HCW

In-depth interviews were conducted with 14/15 (93.3%) HCW at the immunization clinics. The mean age of the HCW interviewed was 33.4 years, and they were predominantly female (64.3%). One of the HCW could not be interviewed as they were absent from work due to ill-health. Themes and sub-themes are summarized in Figure 3. See also supplementary material 5.

Theme 1: General Challenges at the Clinics, independent of the SBs

Sub-theme 1.1: Delivering Health Education

From our discussions, we found out that HCW were expected to deliver 'health talks' in the clinics before immunization sessions. These health talks would typically include information about the vaccines their infants would receive that day, address the general expectations of caregivers, and remind the caregivers when to return for their next appointment. In some cases, HCW would add points that are important for the health of the babies like regular weighing, nutrition, among others.

- "*... we normally conduct pre-clinic health talks… We discuss vaccines, and sometimes we choose other topics… about their health and that of their babies.*" (Male, Rural clinic, 18 months in current position)

Discussions with HCW suggested that these vaccine clinic health talks are not standardised, and the content and quality vary between locations and situations. Immunization activities in facilities were concentrated in the morning hours of the day. While HCW planned pre-clinic health talks at the beginning of every immunization clinic, several of them ended up only briefly summarizing the plan

For peer review only - http://bmjopen.bmj.com/site/about/guidelines.xhtml

before vaccinating, while some resorted to giving caregivers briefs while administering vaccines at the immunization table. Others did not bother to give the health talk at all due to time constraints. This was confirmed by one of the HCW who said:

- *"When we have a large crowd, we also engage them in interpersonal communication while administering the vaccines, we tell them about the vaccines been administered and the possible side effects of the vaccine."* (Male, Rural clinic, 6 months in current position)

Sub-theme 1.2: Attitude of caregiver

Several HCW expressed dissatisfaction regarding the attitude of caregivers. According to them, some caregivers did not show up on their visit appointments, others came late, while others were impatient and always in a hurry to go home. HCW perceived caregivers to value domestic chores and activities more than getting the best out of the immunization clinic visit. This was confirmed by a HCW who said:

- *"When women come to the clinic, they are always in hurry to go back home to cook or going back to the garden, so most times they are in haste to go home."* (Male, Urban clinic, 5 years in current position)

In ensuring caregivers sat and listened to the vaccine health education, HCW developed various means of ensuring they stayed to receive the information. According to one of the HCW:

"What we do sometimes is to hold their cards and start weighing, and then when most of the women are around, we start giving the health education." (Male, Rural clinic, 8 months in current position)

Sub-theme 1.3: Organizational factors

Our discussions with HCW revealed that although clinic schedules were planned in advance, there were a limited number of immunization clinics in a month, especially in rural settings. According to a few HCW, overcrowding and congestion during immunization clinics was common as many of the caregivers visited at the same time on the limited number of days allotted to immunization services.

- *"...most of them [caregivers] come around the same time and the health care worker might not have time to carefully explain in detail to the women ... most times the environment is even very noisy."* (Male, Rural clinic, 3 years in current position)

The clinic setting was sometimes found to be very noisy and chaotic. According to one of the HCW,

- *".... some of the women will be standing and distracted by the cry of their children and may not even have the patience to listen to what we are saying to them." Adding that: "Interpersonal health education is usually better for us with the crowd (large numbers of caregivers)."* (Female, Rural clinic, 3 years in current position)

Our discussions revealed that these challenges cut across facilities in both the urban and rural centres. The HCW further emphasised the importance of the *SB* in filling the gaps of improving the knowledge of caregiver.

For peer review only - http://bmjopen.bmj.com/site/about/guidelines.xhtml

Sub-theme 1.4: Low manpower

Findings revealed that some facilities did not have adequate HCW/Public Health Officers in the clinic to deliver immunization services. Staff shortages were stressful for both HCW and Caregiver, resulting in long waiting time, poor service delivery and dissatisfaction. This was confirmed by one of the HCW who said:

- *"... when we started the implementation of the speaking books, I was the only Public Health Officer here without any form of assistance or back up."* (Male, Urban clinic, 5 years in current position)

Another HCW said:

- *"Sometimes I will be the only one immunizing, weighing and screening so sometimes it's difficult to use the speaking books."* (Male, Urban clinic, 5 years in current position)

Staff shortages are stressful for both HCW and caregivers, resulting in long waiting time, poor service delivery and dissatisfaction.

Theme 2: *SB* Impact

Sub-theme 2.1: Improved Knowledge

HCW revealed that they observed increased knowledge among caregivers as a result of the utilization of the *SB*. They added that the *SB* addresses vaccines issues adequately.

- *"Most times after listening to the books, we ask them questions on immunisation and they are able to give positive feedback."* (Male, Urban clinic, 18 months in current position)
- *"... I cannot assure you on their [all caregivers attending the immunisation clinic] increase in knowledge compared to women who have personal copies [recruited participants] with them at home.*" (Male, Rural clinic, 8 months in current position)

Sub-theme 2.2: Efficiency

Over the course of the discussion, HCW expressed ways the *SB* has increased productivity with their work. Some of the HCW emphasized the importance of the local languages used in the book. The *SB* was judged to be a self-explanatory tool which most of the caregiver could use with little or no assistance.

- *"... for me it saves time because I just hand over the book to them (caregivers) and then they listen to everything... while I concentrate on the work and it makes work faster."* (Female, Rural clinic, 3 years in current position)

Some of the HCW expressed their desire have more copies of the *SB* in the facilities to further increase productivity with their work.

- *"... the issue is we have just one copy in the facility which... is not enough... they (caregivers) all have to keep waiting for each other to listen to the books... if we have at least five copies it would be better than everyone sharing one copy."* (Male, Urban clinic, 18 months in current position)

For peer review only - http://bmjopen.bmj.com/site/about/guidelines.xhtml

Sub-theme 2.3: Informative

Some of the HCW described the *SB* as an informative tool which served the needs of caregivers and HCW. The content was able to address most of the concerns of caregiver and presented vaccine information in a simple way that could be easily understood.

- *"In the past they give so many complains about adverse effects following vaccination……. even blame public health officers that administer the vaccines, they have a better understanding of the side effects after receiving the vaccines and appreciate the information gotten from the book.*" (Female, Rural clinic, 3 years in current position)
- *"It has addressed all the details and relevant information that will make mothers want to bring their children for immunization."* (Female, Rural clinic, 3 years in current position)

Sub-theme 2.4: Improved Clinic Attendance

HCW repeatedly highlighted enhanced clinic attendance by caregiver since the introduction of the *SB*, especially among those with personal copies of the *SB*. Caregivers appeared more motivated and dedicated to the immunization schedule. Some HCW suggested that the *SB* had resulted in increased uptake of vaccination, helping them to meet their coverage targets.

- *"Mothers are beginning to bring their babies to the clinic unlike before when they will just come to the clinic anytime they like.*" (Female, Urban clinic, 10 years in current position)
- *"In the past we used to have low coverages but with the introduction of the speaking books are now seeing more mothers coming.*" (Male, Urban clinic, 5 years in current position)

Theme 3: Outcome of use of the *SB*s

Sub-theme 3.1: Enhanced Communication

Discussions focusing on how the *SB* had improved communication between HCW and caregiver revealed that the two *SB* local languages had aided the sharing of vaccine information. Some HCW expressed their struggle in explaining vaccine preventable diseases in way that could be understood by mothers prior to the introduction of the *SB*.

- *"The book has made it easier for us to explain information on vaccines to the mothers…"* (Female, Urban clinic, 10 years in current position)
- *"… most of the women don't understand English but with the use of the local languages they understand the information easily… It has been easier for us to pass across the information because English is not our language, so it is easier for them to understand and easier for us to explain to them."* (Male, Urban clinic, 3 years in current position)

Sub-theme 3.2: Health Promotion Tool

As the discussions continued, some of the HCW highlighted the importance of the *SB* to them and their colleagues in other facilities. They considered the *SB* an important tool for HCW in general. They discussed the importance of sharing the information in the *SB* with HCW in other facilities, including through recoding the audio on a phone and sharing with colleagues on social media. One HCW said:

For peer review only - http://bmjopen.bmj.com/site/about/guidelines.xhtml

- *"... we are trying, by recording the audio on our phones and sharing with other health staff because not all health officers have access to the information in this book… We learnt a lot from it."* (Male, Urban clinic, 3 years in current position)

Another HCW expressing his own unique way of making use of the *SB* said:

- *"Because the book is not loud for all the mothers to hear, I recorded it on my phone, used a Bluetooth device to connect to a speaker for all the mothers to hear."* (Male, Urban clinic, 2 years in current position)

Sub-theme 3.3: Shared responsibility and community ownership

According to the HCW, some of the women shared information from the *SB* in the community and some women had been eager to take the responsibility of health talks during immunization clinics with their peers. HCW considered this as a way of spreading vaccine-related information and facilitating their own work.

- *"They share during lunch or when they sit together, they play the book."* (Male, Rural clinic, 8 months in current position)
- *"During the immunisation clinic days most of the women who have copies of the speaking books come along with the books and they normally take over the health talks with the use of their speaking books, sometimes I don't even play my own."* (Male, Urban clinic, 5 years in current position)

Further discussion revealed that caregivers made use of the *SB* in family and community gathering.

- "*… in the communities, mothers and caregivers look up to those who have benefitted from the study they are like mentors to other mothers and caregivers in the community where they live."* (Male, Rural clinic, 3 years in current position)

Theme 4: Further Suggestions

Sub-theme 4.1: SB utilization

The opinion of HCW regarding their preferences on the best setting for the utilization of *SB* varied. Some HCW did not make use of the *SB* during very busy clinics but instead preferred to use it when the clinic was quieter. Some suggested the *SB* to be more useful in homes with caregiver as they are able to go through the book at their own convenient time with the added advantage of sharing the *SB* with people in the communities who may not come to the clinic. They believed this would foster transfer of knowledge to the community members.

- *"I think is better they are given the books at their homes… in the clinic most times they are in hurry to go home… at home they will take out time to listen to the book and gain more information compared to listening to the books in the clinic."* (Male, Urban clinic, 5 years in current position)
- *"…. I believe the women will make better use of the speaking books if they are used in the facilities than in their homes ... at the health facility the health workers will be around to guide them, this will ensure a better use of the book…"* (Male, Urban clinic, 18 months in current position)

For peer review only - http://bmjopen.bmj.com/site/about/guidelines.xhtml

Sub-theme 4.2: Changes to the SB

The low sound volume of the *SB* remained a consistent challenge expressed by the HCW during the discussions; some even suggested modifying the *SB* so it could be connected to external devices that could amplify the volume.

- *"The only problem the speaking book has is that it cannot be used with a large audience because the volume is very low."* (Female, Rural clinic, 3 years in current position)
- *"….. it will be good to have a book that is very loud so we don't have to talk, we will just place it at the front of the Clinic, press the buttons and the book will start playing for the women to listen to it."* (Male, Rural clinic, 6 months in current position)

Sub-theme 4.3: Additions to the SB

Constraints with optimal utilization of the *SB* were linked to the use of only two languages in the audio translation of the book. In addition to the challenges faced in recruiting caregivers for this study, HCW further suggested the need to incorporate additional languages to make use of the book with more caregivers of different language backgrounds.

- *"… other languages need to be added…. because caregivers who do not understand any of the languages used in the book did not benefit from the information in the book…"* (Male, Urban clinic, 4 years in current position)

Some HCW suggested other health topics which could be added to the vaccines *SB*, such as nutrition and maternal health.

- *"…. we can also consider adding information on nutrition and importance of antenatal care by pregnant women." (Female, Urban clinic, 10 years in current position)*

DISCUSSION

In this study, we assessed the impact of a bespoke, multi-media educational tool, the vaccine *Speaking Book®*, on vaccine-related knowledge of caregivers as well as on communication between HCW and caregivers in The Gambia. We distributed this tool to 113 caregivers visiting PHC for routine immunization services and evaluated changes in vaccine knowledge over a three-month period.

We saw significant improvement in the knowledge scores with the median scores nearly doubling at 1-month and nearly tripling at 3-month follow-up visits, compared to the baseline scores, independent of region of the country (urban or rural), age of caregiver, household income or highest level of education. The *SB* was given to the caregivers to take home, thus giving them several opportunities to listen to and understand the information in the book, which might have contributed to the ongoing improvement over the 3-month period.

Study participants discussed and shared content of the *SB* extensively within their family, community, and social groups, using a variety of platforms such as peer group meetings, community and religious gatherings. This is likely to lead to a significant and possibly measurable multiplier effect. Our results

For peer review only - http://bmjopen.bmj.com/site/about/guidelines.xhtml

support the usefulness of the *SB* beyond individuals' knowledge gain and in both the health facility and community setting. Our findings of "added value" are similar to observations by other investigators who used a similar tool.[17, 19] One study in South Africa reflected the use of a *SB* tool as significant in increasing knowledge on Clinal trials among recruited study participants.[17] Another study reflected the effectiveness of the tool in increasing knowledge of biobanking and genetics among a group of non-academic university staff in South Africa. This qualitative experimental study reported a significant increase overall knowledge score, similar to our findings, this increase in knowledge score did not occur for all questions.[17, 19]

Our results support the importance of providing targeted, vaccine-related education to caregivers and communities as an effective and practical strategy to improve vaccine-related knowledge in low literacy settings such as The Gambia. [20, 21]

Our data is comparable to findings from a previous study conducted by our team in The Gambia[22] where the use of the *SB* equally resulted in significant improvement in knowledge gained among study participants. However, the previous speaking book was directed at providing information about a specific clinical trial of pneumococcal vaccines, not the EPI program and vaccine-related knowledge in general. It was also conducted in a community with previous exposure to vaccine research, a setting we deliberately avoided in the project presented here as it is likely to lead to significant bias. Illustrations in the previous *SB* had also not been adapted to the local Gambia context. During the stakeholder engagement for our study, we received feedback that led to specific alterations with regards to dress code for example, and to derive a *SB* that reflects local communities was an important message received.

Studies have reported high baseline knowledge score of study participants as a limitation in generalizing improvement in knowledge as a result of the use of a *SB*. This, they believe, may be a possibility of 'guesswork' as a result of the simplicity of the concept used in explaining the subject matter.[17, 19] However, findings from our study showed consistency in increased vaccine knowledge at baseline compared to subsequent visits.

While the *SB* was able to significantly improve the vaccine-related knowledge, a knowledge gap remained: although we reported significant improvements in the mean knowledge scores from a very low baseline, the highest score obtained was 19.0, still 20 points below the maximum possible score of 39. It is possible that the detailed knowledge needed to obtain these remaining points is more difficult to retain compared to some other answers and we wondered if there was an educational bias. The subgroup of caregivers with tertiary education did not achieve significantly higher scores, maybe because they already had a higher baseline score. We are unable to draw firmer conclusions about the impact of higher education, since there were only two participants in this group. It is entirely possible that the knowledge score developed was overambitious.

For peer review only - http://bmjopen.bmj.com/site/about/guidelines.xhtml

A majority of the caregivers reported that HCW were their usual source of information about vaccines. Hence further training and retraining the HCW to deliver the additional information to the caregivers could significantly contribute to reducing the remaining knowledge gap.[23] In a study similar to ours, the *SB* was used regularly for health promotion in health posts, home visits and local gatherings and HCW referred to the *SB* as a "job aid".[15] They carried out observations, structured interviews and focus group discussions with HCW within a selected region of Ethiopia, this informed their reports on the contribution, appropriateness, and challenges of the *SB*.[15]

Most caregivers had listened to the *SB* multiple times and suggested that it would be important for mothers to receive a copy of the *SB* at their first clinic visit, especially while pregnant or when their babies are newly born. Studies have reported distribution of the *SB* to study participants who have either had to opportunity to spend days with the tool, use the tool at workstations or take the tool home as their personal property.[15, 22] Trust in the messages provided by the book was almost unanimous and the illustrations, which were adapted to the local context, were approved by all participants. The initial stakeholder consultation in the design phase of the book was therefore very important. We highly recommend to involve the local communities in the design of such tools, as their identification with the visual materials is likely to increase its acceptancy.[12, 16]

Results obtained from the HCW interviews demonstrated that the *SB* had empowered them to answer concerns and questions of caregivers, emphasising the importance of the local language translations which presented vaccine information in a simple, easy to understand manner. HCW reported increase in productivity and improved clinic attendance and considered the *SB* as an important health promotion tool for both HCW and caregivers in the Gambia. It was evident that the *SB* had a positive impact on their day-to-day practice, and its content was well received, which facilitated communication on vaccines.

Vaccine confidence remains high in countries like The Gambia, but in the overall context of increasing vaccine hesitancy worldwide, tools such as the *SB* or similar approaches tailored to local context might add significant value to engage individuals and communities in communication about the personal and societal value of vaccination. They could be an important counter-measure to vaccine hesitancy.[24-26] It is interesting to note that none of the participants reported obtaining their vaccine-related information from the media, unlike in many high-income countries.

We acknowledge the lack of a control group in our study, but this was beyond our means in this project. We agree that the inclusion of a control group not receiving the *SB* would have been desirable but funding restrictions did not allow us to implement this design as we needed to make sure we had a large enough sample of caregivers who actually received the *SB* and were also representative of the country. This included urban and rural areas and a different set up of health clinics. Further

For peer review only - http://bmjopen.bmj.com/site/about/guidelines.xhtml

evaluations could be carried out in a properly designed and powered trial in the future and we believe that our study provides useful data for the conduct of such a study.

Overall vaccine literacy is more complex than “just” being knowledgeable about schedules and target diseases of the vaccines provided in the EPI schedule, which was the focus of most of our knowledge test. It encompasses understanding, appraisal and application of vaccine-related information services, additional components not captured in this project.

Further research will now be required to quantify any potential effect on vaccine uptake at the health center level and measure cost-effectiveness. In discussions with the Gambian EPI program, both are expressed areas of interest. The *SB* used in this study was only recorded in two of the locally spoken languages (*Wolof* and *Mandinka*) in The Gambia and therefore, excluded caregivers who did not speak or understand either of these languages. We were asked to produce books in additional local languages in the future as well as design books for additional topics, such as pregnancy and nutrition.

CONCLUSION

In conclusion, the vaccine *Speaking Book*® is a health education tool that resulted in significant increase in vaccine-related knowledge of caregivers, irrespective of their age, location, household income or educational level. It also served as a valuable tool for HCW in their interactions with caregivers and might lead to increased uptake of immunization services.

Contributors

BK and SK conceptualised and designed the study. ON, OW and AC were involved in data collection. and analysed the data. ON wrote the first draft; BK, SK, OW, PJ and MK provided critical feedback and helped shape the research, analysis and manuscript. All the authors revised the work for important intellectual content and contributed to the final draft and finally approved it to be published.

Acknowledgements

We thank the field assistants for detailed data collection and transcription from the local languages to English for data analysis. We thank Alexander Jarde for statistical support in the analysis of the study data. We also thank the heads of the participating health facilities and members of staff for granting us access to the EPI clinics and their support throughout the project.

Competing interests

None.

Funding

This study was funded by a project grant from Bull City Learning. BK is supported by funding from the UKRI (MC_UP_A900/1122). The Vaccines & Immunity research led by BK is jointly funded by the UK Medical Research Council (MRC) and the UK Department for International Development

For peer review only - http://bmjopen.bmj.com/site/about/guidelines.xhtml

(DFID) under the MRC/DFID Concordat agreement and is also part of the EDCTP2 programme supported by the European Union.

For peer review only

For peer review only - http://bmjopen.bmj.com/site/about/guidelines.xhtml

REFERENCES

1. Ventola CL. Immunization in the United States: Recommendations, Barriers, and Measures to Improve Compliance: Part 1: Childhood Vaccinations. P t. 2016 Jul;41(7):426-36
2. Negussie A, Kassahun W, Assegid S, Hagan AK. Factors associated with incomplete childhood immunization in Arbegona district, southern Ethiopia: a case--control study. BMC Public Health. 2016 Jan 12;16:27
3. Nnaji CA, Owoyemi AJ, Amaechi UA, Wiyeh AB, Ndwandwe DE, Wiysonge CS. Taking stock of global immunisation coverage progress: the gains, the losses and the journey ahead. Int Health. 2020 Jan 11
4. Esposito S, Principi N, Cornaglia G. Barriers to the vaccination of children and adolescents and possible solutions. Clin Microbiol Infect. 2014 May;20 Suppl 5:25-31
5. Sato R, Fintan B. Fear, knowledge, and vaccination behaviors among women in Northern Nigeria. Hum Vaccin Immunother. 2020 Feb 21:1-11
6. Ianni A, Tedeschi R, Marchetti A, Basso D, Virgili R, Piredda M, et al. [The role of nurses in health education about vaccines: analysis of style and communication models of institutional vaccination campaigns]. Ig Sanita Pubbl. 2019 Sep-Oct;75(5):355-69
7. Gil Cuesta J, Whitehouse K, Kaba S, Nanan-N'Zeth K, Haba B, Bachy C, et al. 'When you welcome well, you vaccinate well': a qualitative study on improving vaccination coverage in urban settings in Conakry, Republic of Guinea. Int Health. 2020 Jan 13
8. Smith LE, Webster RK, Weinman J, Amlôt R, Yiend J, Rubin GJ. Psychological factors associated with uptake of the childhood influenza vaccine and perception of post-vaccination side-effects: A cross-sectional survey in England. Vaccine. 2017 Apr 4;35(15):1936-45
9. Owais A, Hanif B, Siddiqui AR, Agha A, Zaidi AK. Does improving maternal knowledge of vaccines impact infant immunization rates? A community-based randomized-controlled trial in Karachi, Pakistan. BMC Public Health. 2011 Apr 17;11:239
10. Guzman-Holst A, DeAntonio R, Prado-Cohrs D, Juliao P. Barriers to vaccination in Latin America: A systematic literature review. Vaccine. 2020 Jan 16;38(3):470-81
11. Damnjanović K, Graeber J, Ilić S, Lam WY, Lep Ž, Morales S, et al. Parental Decision-Making on Childhood Vaccination. Front Psychol. 2018;9:735
12. Castelnuovo B, Newell K, Manabe YC, Robertson G. Multi-Media Educational Tool Increases Knowledge of Clinical Trials in Uganda. J Clin Res Bioeth. 2014 Jan 2;5:165
13. Tefera YA, Wagner AL, Mekonen EB, Carlson BF, Boulton ML. Predictors and Barriers to Full Vaccination among Children in Ethiopia. Vaccines (Basel). 2018 Apr 10;6(2)
14. Wariri O, Edem B, Nkereuwem E, Nkereuwem OO, Umeh G, Clark E, et al. Tracking coverage, dropout and multidimensional equity gaps in immunisation systems in West Africa, 2000-2017. BMJ Glob Health. 2019;4(5):e001713

For peer review only - http://bmjopen.bmj.com/site/about/guidelines.xhtml

15. Endale E, Tesfaye S. Rapid Qualitative Assessment of Maternal and Newborn Health Care (MNHC) Speaking Book in Two Districts in the Amhara Region, Ethiopia. UNICEF 2012.
16. Afolabi MO, McGrath N, D'Alessandro U, Kampmann B, Imoukhuede EB, Ravinetto RM, et al. A multimedia consent tool for research participants in the Gambia: a randomized controlled trial. Bull World Health Organ. 2015 May 1;93(5):320-8a
17. Dhai A, Etheredge H, Cleaton-Jones P. A pilot study evaluating an intervention designed to raise awareness of clinical trials among potential participants in the developing world. J Med Ethics. 2010 Apr;36(4):238-42
18. Braun V, Clarke V. Using thematic analysis in psychology. Qual Res Psychol. 2006;3(2):77-101
19. Bedeker A, Anderson D, Lose T, Mgwatyu Y, Luus R, Blignaut R, et al. Understanding biobanking: An assessment of the public engagement speaking book intervention Biobanking and Me. South African J Bioeth Law. 2019;12(2):87
20. Masters NB, Tefera YA, Wagner AL, Boulton ML. Vaccine hesitancy among caregivers and association with childhood vaccination timeliness in Addis Ababa, Ethiopia. Hum Vaccin Immunother. 2018;14(10):2340-7
21. Oku A, Oyo-Ita A, Glenton C, Fretheim A, Ames H, Muloliwa A, et al. Perceptions and experiences of childhood vaccination communication strategies among caregivers and health workers in Nigeria: A qualitative study. PLoS One. 2017;12(11):e0186733
22. Mboizi RB, Afolabi MO, Okoye M, Kampmann B, Roca A, Idoko OT. Recall and decay of consent information among parents of infants participating in a randomized controlled clinical trial using an audio-visual tool in The Gambia. Hum Vaccin Immunother. 2017 Sep 2;13(9):2185-91
23. Arogundade L, Akinwumi T, Molemodile S, Nwaononiwu E, Ezika J, Yau I, et al. Lessons from a training needs assessment to strengthen the capacity of routine immunization service providers in Nigeria. BMC Health Serv Res. 2019 Sep 14;19(1):664
24. Dubé E, Gagnon D, MacDonald NE. Strategies intended to address vaccine hesitancy: Review of published reviews. Vaccine. 2015 Aug 14;33(34):4191-203
25. Wellcome. World survey reveals people trust experts but want to know more about science [cited 2020 Feb 5]. Available from: https://wellcome.ac.uk/news/world-survey-reveals-people-trust-experts-want-know-more-about-science.
26. World Health Organization. Improving vaccination demand and addressing hesitancy [cited 2020 Feb 5]. Available from: https://www.who.int/immunization/programmes_systems/vaccine_hesitancy/en/.

For peer review only

For peer review only - http://bmjopen.bmj.com/site/about/guidelines.xhtml

FIGURE LEGENDS

Figure 1: Photo of the *Speaking Book*®

Figure 2: Flow chart of participant recruitment process

Figure 3: Themes and sub-themes from in-depth interviews with healthcare workers

For peer review only

For peer review only - http://bmjopen.bmj.com/site/about/guidelines.xhtml

Figure 1A

Vaccines Save Children's Lives

(How Vaccines Protect Your Baby)

VACCINES SAVE CHILDREN'S LIVES (HOW VACCINES PROTECT YOUR BABY)

ENGLISH, WOLOF AND MANDINKA

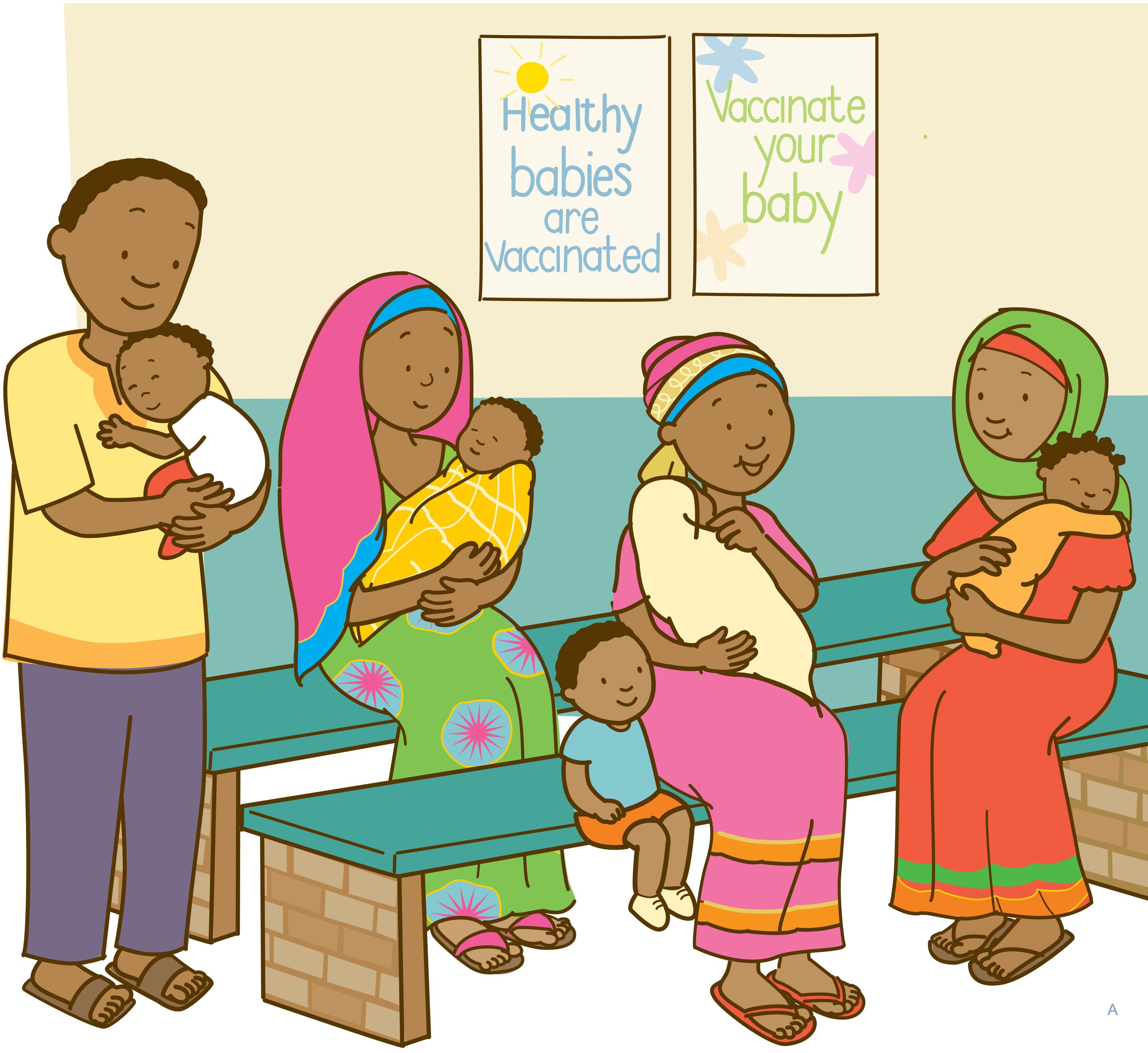

This book was developed by the MRC Unit The Gambia at the London School of Hygiene and Tropical Medicine in collaboration with The Gambia Expanded Programme on Immunization

For peer review only - http://bmjopen.bmj.com/site/about/guidelines.xhtml

TEXT: ENGLISH AUDIO: WOLOF AND MANDINKA

Figure 1B

Vaccines Save Children's Lives

(How Vaccines Protect Your Baby)

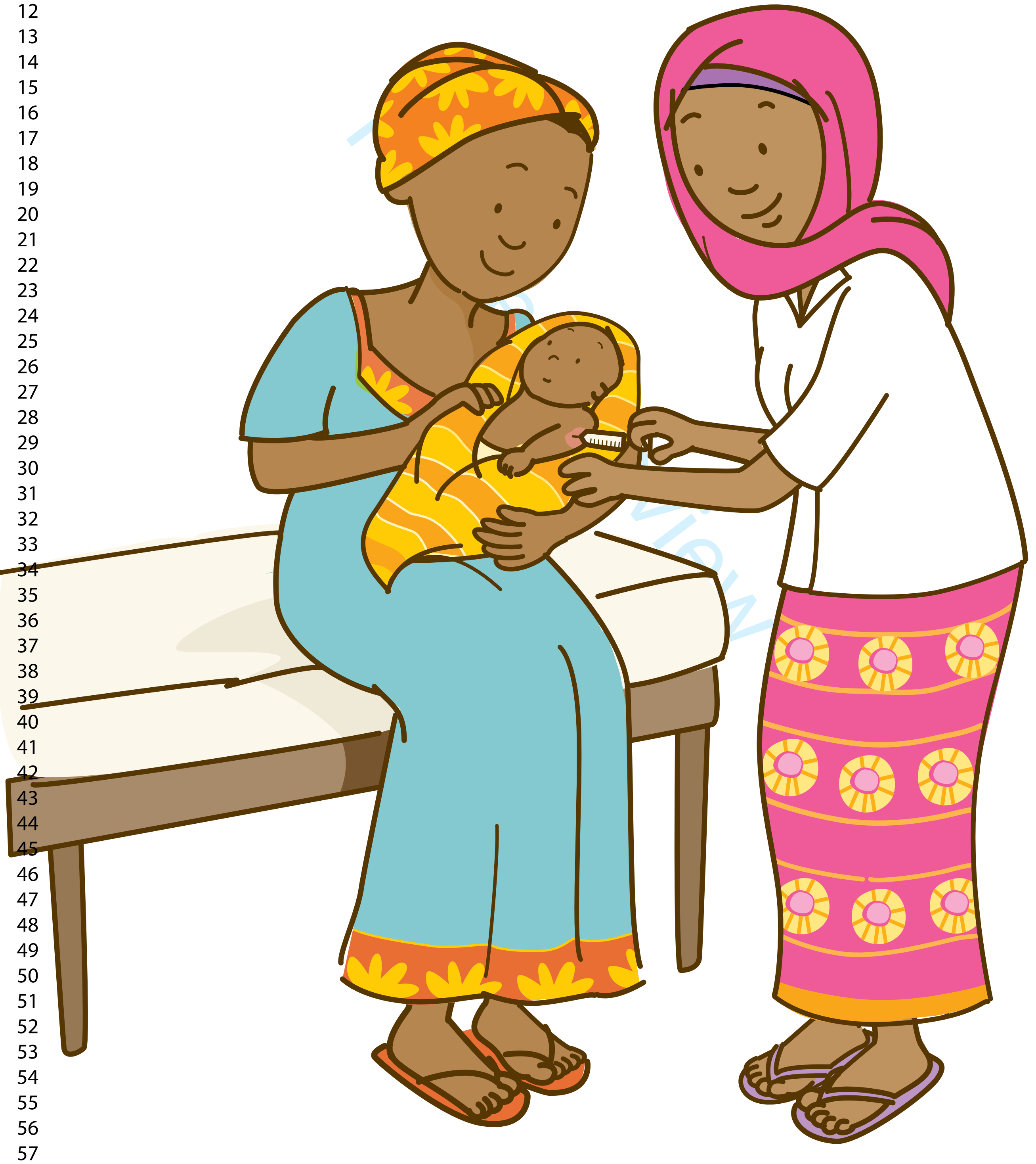

Press the buttons to hear about how
Vaccines Save Children's Lives

For peer review only - http://bmjopen.bmj.com/site/about/guidelines.xhtml

Figure 1C

Does my child need to receive all vaccines recommended by the government?

Your baby needs to receive all vaccines given by the government. This is to make sure they're protected and can't get sick from infections prevented by the vaccines. The vaccines are free in government health facilities. Always take your Infant Welfare Card for the health workers to check. They'll tell you which vaccines your baby has received. They'll also tell you when you need to bring your baby back.

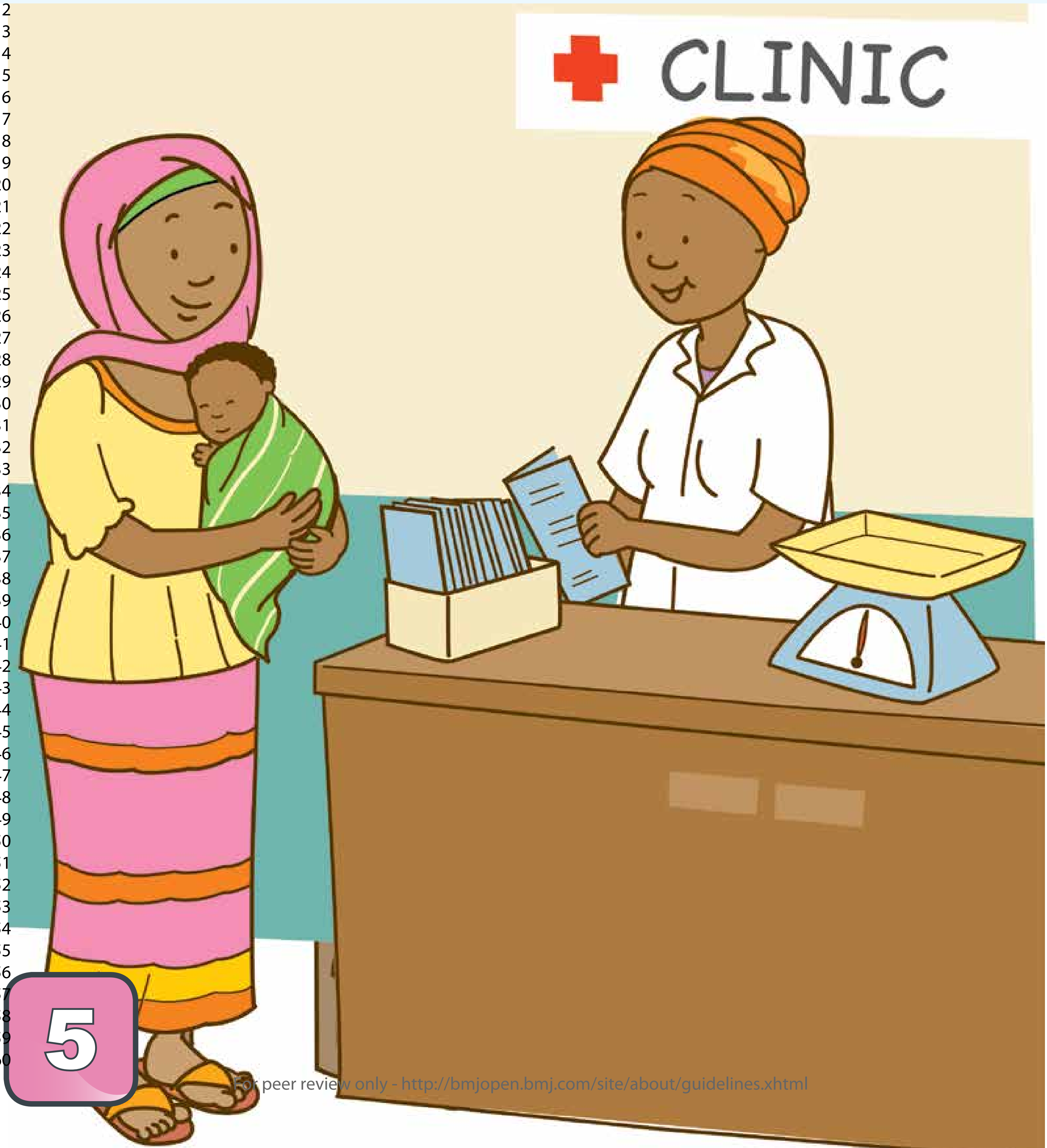

Figure 1D

Is it important that my child receives all vaccines and on time?

It's very important that your baby receives their vaccines on time - at birth, 2 months, 3 months, 4 months, 9 months and 18 months. Vaccines protect babies early in life when they're most at risk of infection and are exposed to many illnesses and diseases. When your baby has not received all the required vaccines or the vaccines are late, they aren't protected.

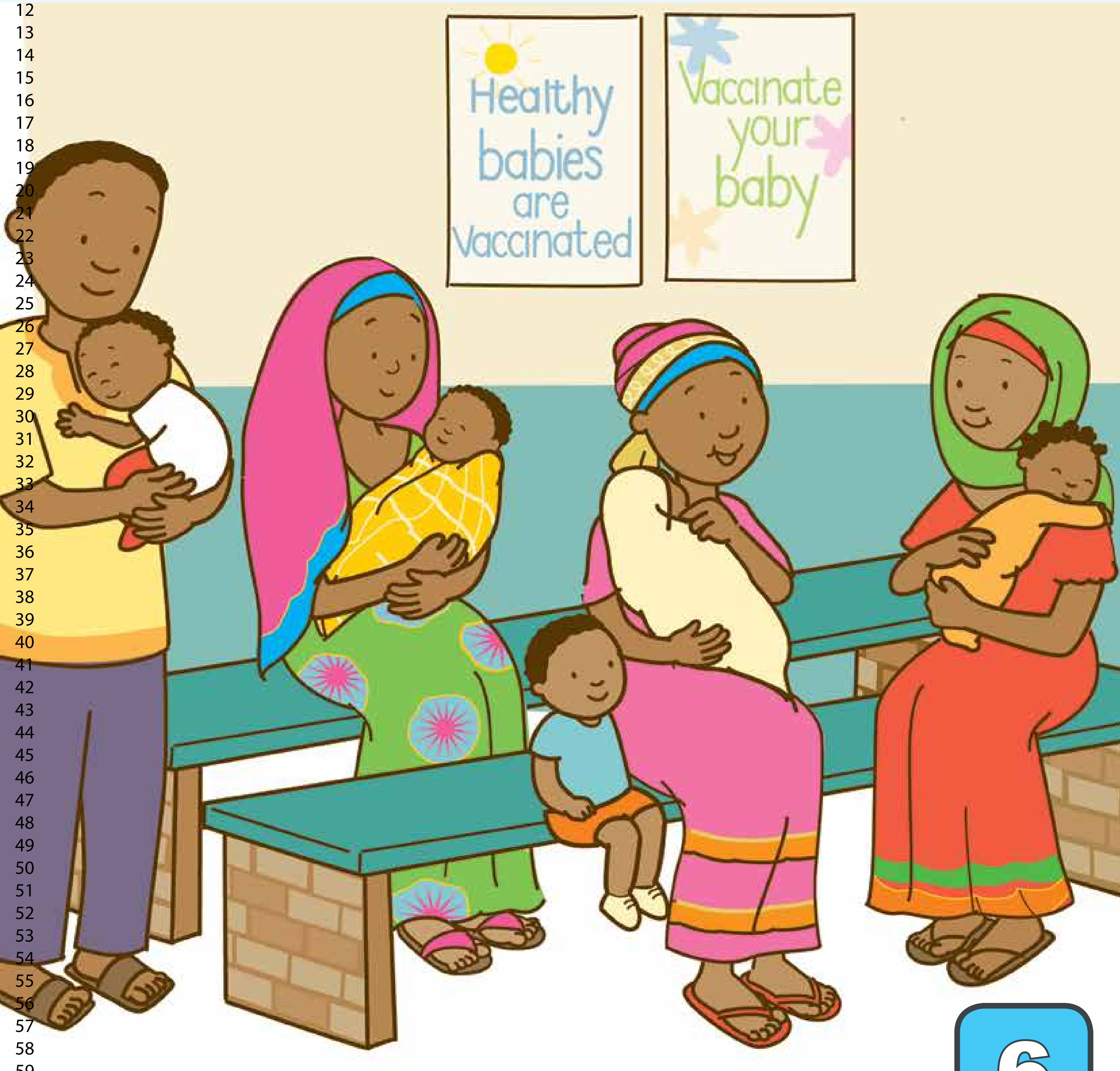

6

Figure 2

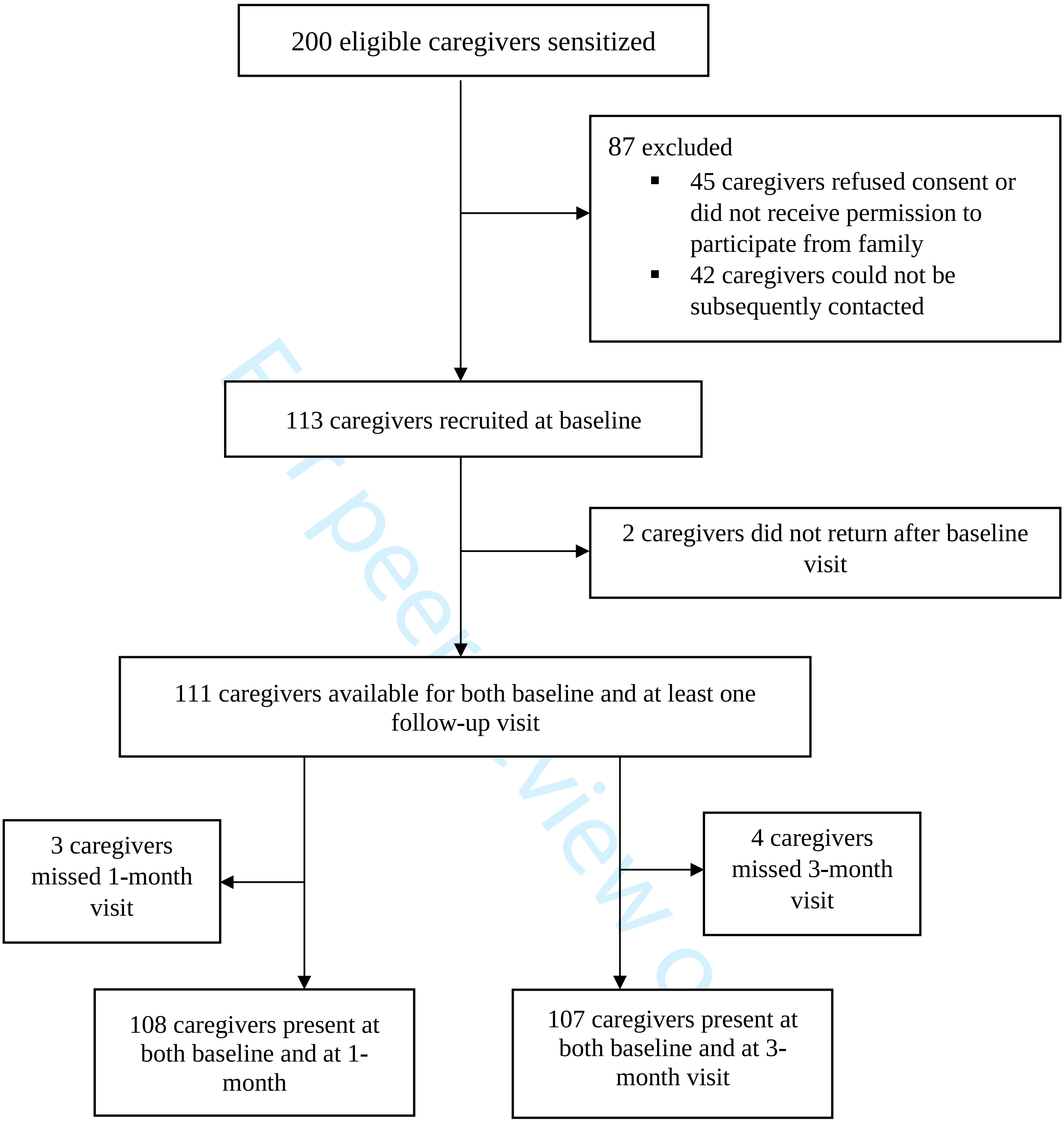
200 eligible caregivers sensitized
87 excluded
45 caregivers refused consent or did not receive permission to participate from family
42 caregivers could not be subsequently contacted
113 caregivers recruited at baseline
2 caregivers did not return after baseline visit
111 caregivers available for both baseline and at least one follow-up visit
3 caregivers missed 1-month visit
4 caregivers missed 3-month visit
108 caregivers present at both baseline and at 1-month
107 caregivers present at both baseline and at 3-month visit

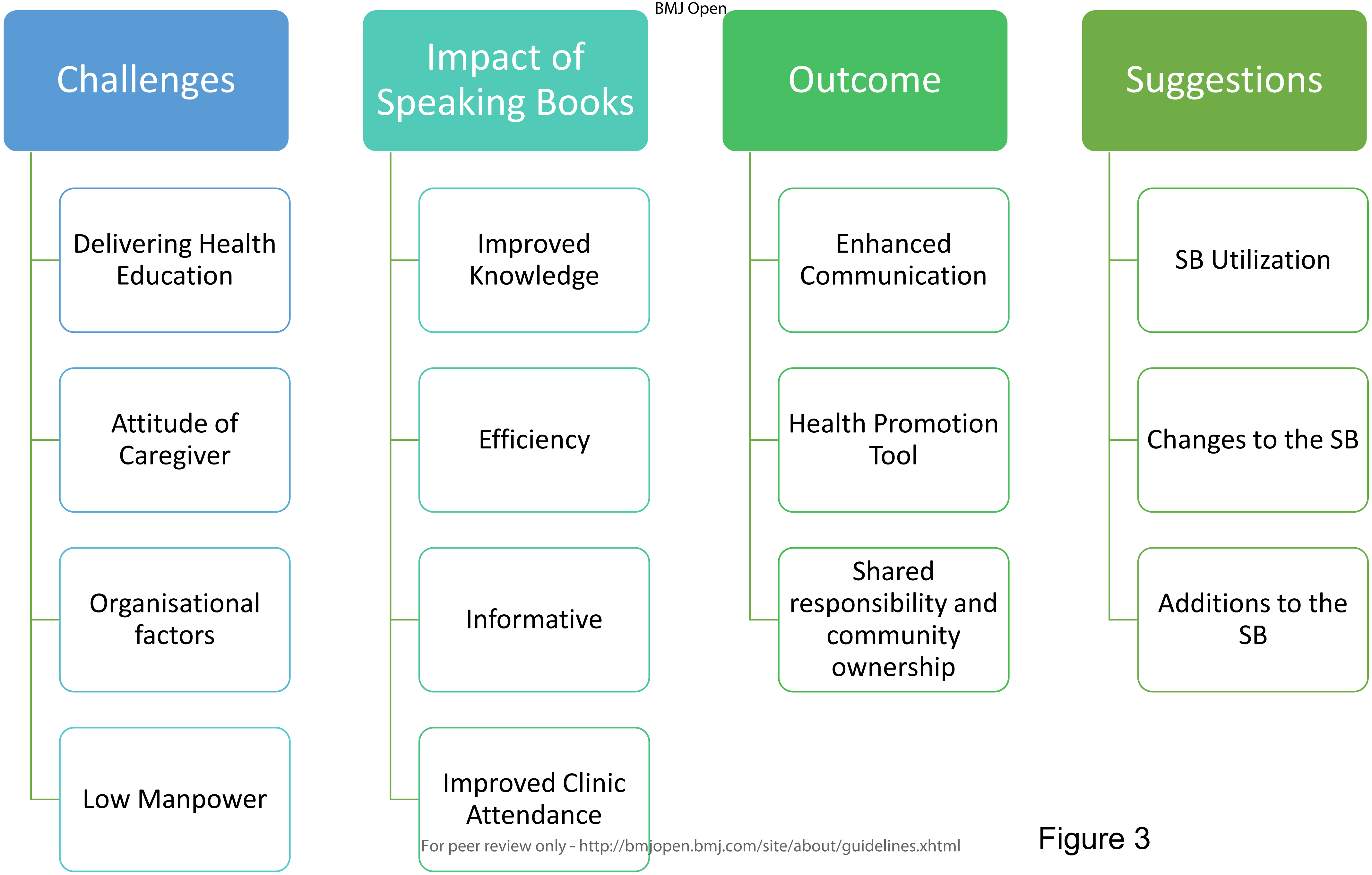
Challenges
Delivering Health Education
Attitude of Caregiver
Organisational factors
Low Manpower
Impact of Speaking Books
Improved Knowledge
Efficiency
Informative
Improved Clinic Attendance
Outcome
Enhanced Communication
Health Promotion Tool
Shared responsibility and community ownership
Suggestions
SB Utilization
Changes to the SB
Additions to the SB

Figure 3

Supplementary material 1_Photos of the Speaking Book

Photos of the speaking book

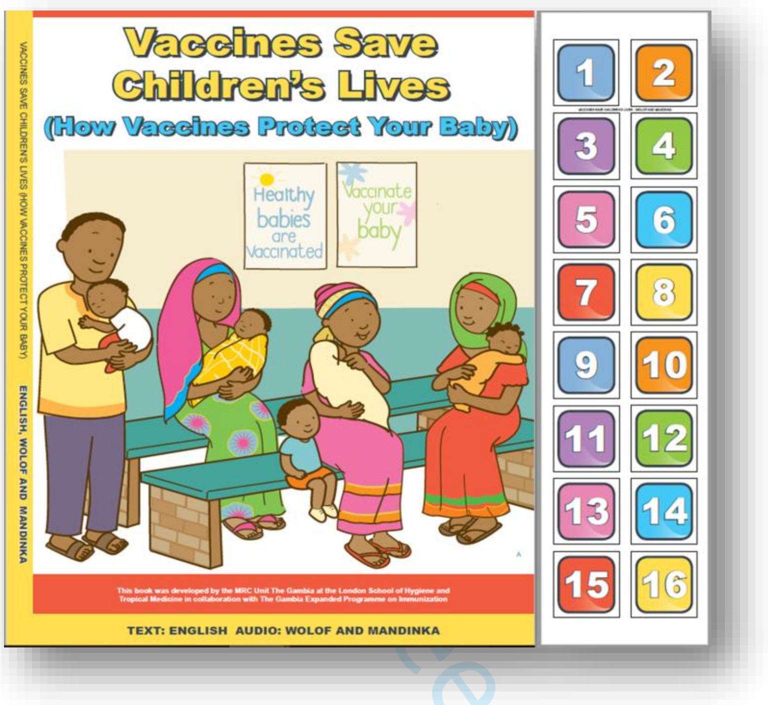

Front view of the SB

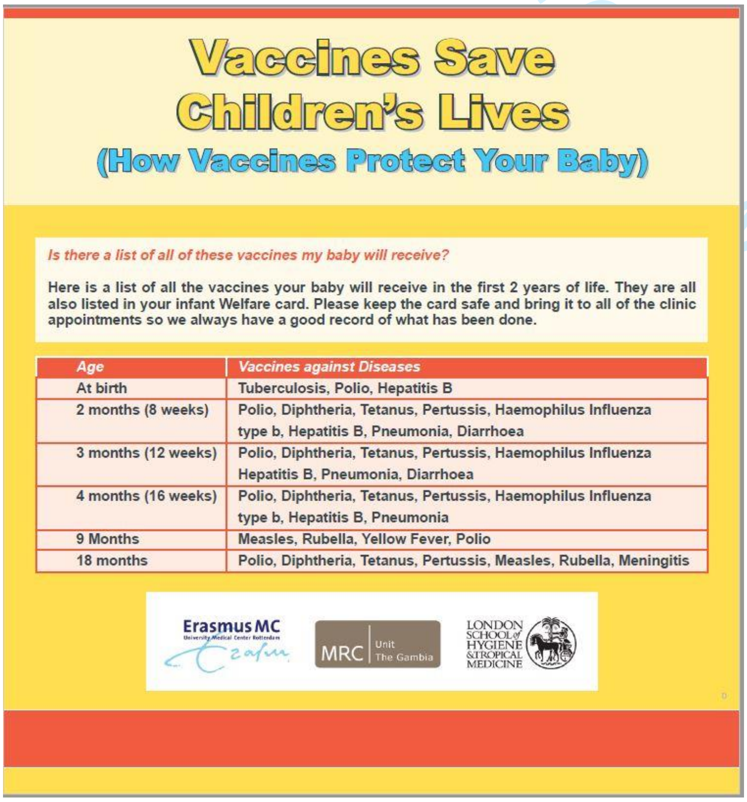

Vaccines Save Children's Lives

(How Vaccines Protect Your Baby)

Is there a list of all of these vaccines my baby will receive?

Here is a list of all the vaccines your baby will receive in the first 2 years of life. They are all also listed in your infant Welfare card. Please keep the card safe and bring it to all of the clinic appointments so we always have a good record of what has been done.

Age	Vaccines against Diseases
At birth	Tuberculosis, Polio, Hepatitis B
2 months (8 weeks)	Polio, Diphtheria, Tetanus, Pertussis, Haemophilus Influenza type b, Hepatitis B, Pneumonia, Diarrhoea
3 months (12 weeks)	Polio, Diphtheria, Tetanus, Pertussis, Haemophilus Influenza Hepatitis B, Pneumonia, Diarrhoea
4 months (16 weeks)	Polio, Diphtheria, Tetanus, Pertussis, Haemophilus Influenza type b, Hepatitis B, Pneumonia
9 Months	Measles, Rubella, Yellow Fever, Polio
18 months	Polio, Diphtheria, Tetanus, Pertussis, Measles, Rubella, Meningitis

Back view of the SB

For peer review only

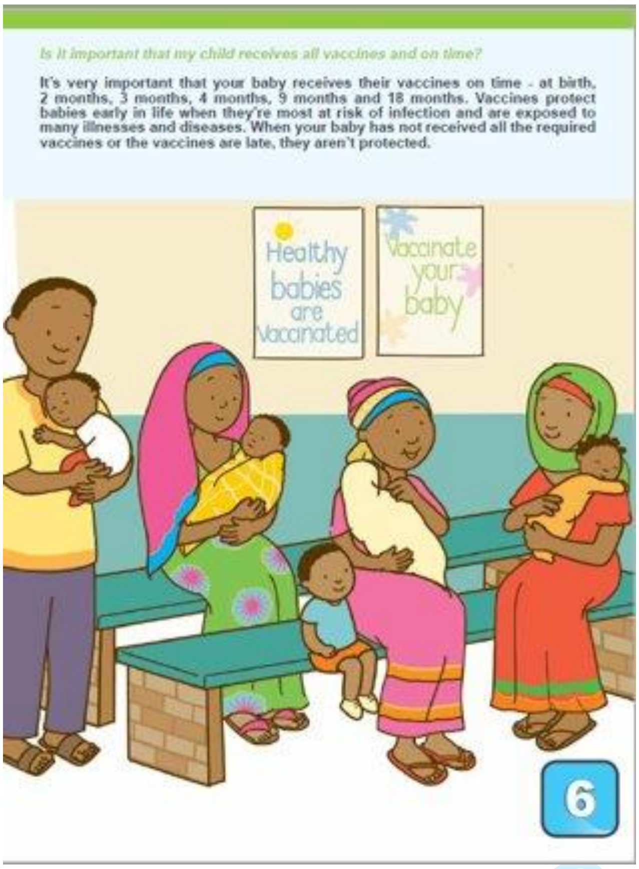

Page 6 of the SB

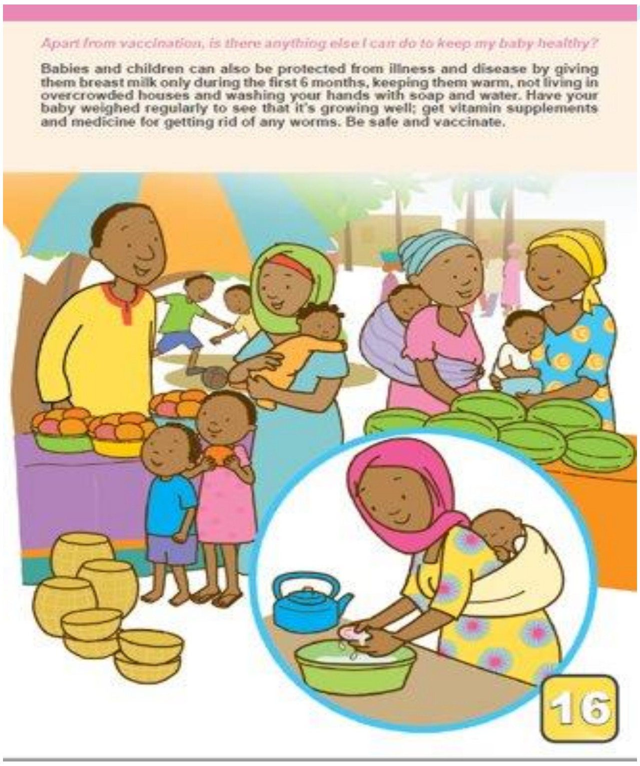

Page 16 of the SB

MRC Unit The Gambia at the
London School of Hygiene
and Tropical Medicine

Study Title: The Vaccines Speaking Book Project

SCC:	1598	Version:	2.1

Sponsor & Funder: MRC & Bull City Learning

Primary Care Givers for the Implementation Study: Section A

Record ID: ___________________________________

Facility name: ___________________________________

Field worker initials: ___________________________________

Participants ID: ___________________________________

Date of entry: ___________________________________

Primary Care Givers (PCGs)

1. Full Name: ___________________________________
2. Contact details (Please collect all available numbers):

   ____

   ____
3. Detailed Address (You can draw behind):

   ____

   ____
4. Age (years): ___________________________________
5. Sex:
   - ☐ 1. Female
   - ☐ 2. Male
6. Religion:
   - ☐ Muslim
   - ☐ Christian
   - ☐ Others

6a. If others, specify ___________________________________

7. Marital status:
   - ☐ Single
   - ☐ Married
   - ☐ Widowed
   - ☐ Others

7a. If others, specify ___________________________________

8. Highest level of Education (Primary care giver):
   - ☐ No education
   - ☐ Primary (Grade 1-6)

For peer review only - http://bmjopen.bmj.com/site/about/guidelines.xhtml

SCC:	1598	Version v 1.2

☐ Secondary (Grade 7–9)
☐ Higher Secondary (Grade 10–12)
☐ Tertiary (University education)
☐ Others

8a. If others, specify ______________________________

9. Education level of other parent (If applicable): ______________________________

10. Employment status:
☐ Employed
☐ Self Employed
☐ Unemployed

11. Employment status of other parent (If applicable):
☐ Employed
☐ Self Employed
☐ Unemployed

12. Occupation: ______________________________

13. Occupation of other parent if applicable: ______________________________

14. Monthly household income:
☐ I don't know
☐ Below D2500
☐ D2, 500-D5, 000
☐ D5, 000- D10, 000
☐ D10, 000- D20, 000
☐ Above D20, 000

15. Presence of a working radio in the house:
☐ Yes
☐ No

16. Presence of a working television in the house:
☐ Yes
☐ No

17. Family size (number of people eating from one pot): ______________________________

18. Mothers age at marriage (years): ______________________________

19. Age at first child (years): ______________________________

IMMUNISATION

20. Mothers receipt of Tetanus Toxoid during pregnancy:
☐ Yes
☐ No

21. How many times: ______________________________

22. Distance from home to Health Facility (Minutes/hours): ______________________________

23. Source of information regarding Immunisation:
☐ Healthcare Worker
☐ Information written on the vaccination cards
☐ Family member or friends
☐ Radio/TV
☐ Poster
☐ Others

23a. If others, specify: ______________________________

For peer review only - http://bmjopen.bmj.com/site/about/guidelines.xhtml

SCC:	1598	Version v 1.2

24. What do you consider to be the challenge or problem you face attending immunization clinic
____
____

CHILD

25. Age: ____________________
26. Date of Birth: (DD/MON/YEAR) ____________________
27. Sex:
☐ Female
☐ Male
28. Birth order: ____________________
29. Date of starting vaccination: ____________________
30. Evidence of completeness of immunizations if available:
☐ Yes
☐ No
☐ Information not available
31. Place of Delivery of this child:
☐ Home
☐ Health Facility
☐ Others

31a. If others, specify: ____________________

32. Mother's utilization of ANC during the pregnancy:
☐ Yes
☐ No
33. How many visits? ____________________

For peer review only

SCC:	1598	Version v 1.2

Primary Care Givers for the Implementation Study: Section B

Record ID: ______________________________________

Facility name: ______________________________________

Field worker initials: ______________________________________

Participants ID: ______________________________________

Date of entry: ______________________________________

1. How do vaccines work?
   - ☐ Vaccines help a baby or a pregnant woman's body fight illness and diseases
   - ☐ Vaccines work by making security guards in the body called 'anti-bodies'
   - ☐ I don't know
   - ☐ Others

1a. If others specify: ______________________________________

2. Why are vaccines given to pregnant women?
   - ☐ Vaccines ensure mother and child are protected against infectious diseases
   - ☐ If the pregnant woman is vaccinated, the baby is also protected for a little while after birth
   - ☐ I don't know
   - ☐ Others

2a. If others, specify: ______________________________________

3. What do you do if your child is not well on the day the vaccines need to be given?
   - ☐ I will take my child to the hospital for the doctor or nurse to decide if my child can be immunised
   - ☐ I will wait at home until my child is well
   - ☐ I don't know
   - ☐ Others

3a. If others, specify: ______________________________________

4. Do you think it is proper for your child to be given more than one vaccine at a visit?
   - ☐ Yes, different vaccines prevent different infections/diseases
   - ☐ No, administering more than one vaccine at a time prevents the vaccine from working effectively
   - ☐ I don't know
   - ☐ Others

4a. If others, specify: ______________________________________

5. Why do you think your child is sometimes given the same vaccines more than once?
   - ☐ Repeat doses are needed to make the antibodies (body soldiers) stronger
   - ☐ Repeat doses help the baby's body learn to be strong against infections for a long time
   - ☐ I don't know
   - ☐ Others

For peer review only
For peer review only - http://bmjopen.bmj.com/site/about/guidelines.xhtml

SCC:	1598	Version v 1.2

5a. If others, specify: ____
____
____

6. When do I need to consider the adverse event of a vaccine serious?
- ☐ If my baby has high fever for more than a day
- ☐ If my baby is sleeping a lot
- ☐ I don't know
- ☐ Others

6a. If others, specify: ____
____
____

7. What vaccines does your baby receive at each visit? Tick all applicable

7a. At birth
- ☐ Tuberculosis
- ☐ Polio
- ☐ Hepatitis B
- ☐ Diphtheria
- ☐ Whooping Cough
- ☐ Meningitis
- ☐ Pneumonia
- ☐ Diarrhoea
- ☐ Measles
- ☐ Yellow Fever
- ☐ I don't know

7b. At 2 Months
- ☐ Tuberculosis
- ☐ Polio
- ☐ Hepatitis B
- ☐ Diphtheria
- ☐ Whooping Cough
- ☐ Meningitis
- ☐ Pneumonia
- ☐ Diarrhoea
- ☐ Measles
- ☐ Yellow Fever
- ☐ I don't know

7c. At 3 Months
- ☐ Tuberculosis
- ☐ Polio
- ☐ Hepatitis B
- ☐ Diphtheria
- ☐ Whooping Cough
- ☐ Meningitis
- ☐ Pneumonia
- ☐ Diarrhoea
- ☐ Measles
- ☐ Yellow Fever
- ☐ I don't know

7d. At 4 Months
- ☐ Tuberculosis
- ☐ Polio

For peer review only

SCC:	1598	Version v 1.2

☐ Hepatitis B
☐ Diphtheria
☐ Whooping Cough
☐ Meningitis
☐ Pneumonia
☐ Diarrhoea
☐ Measles
☐ Yellow Fever
☐ I don't know

7e. At 9 Months

☐ Tuberculosis
☐ Polio
☐ Hepatitis B
☐ Diphtheria
☐ Whooping Cough
☐ Meningitis
☐ Pneumonia
☐ Diarrhoea
☐ Measles
☐ Yellow Fever
☐ I don't know

7f. At 18 Months

☐ Tuberculosis
☐ Polio
☐ Hepatitis B
☐ Diphtheria
☐ Whooping Cough
☐ Meningitis
☐ Pneumonia
☐ Diarrhoea
☐ Measles
☐ Yellow Fever
☐ I don't know

8. What other ways can you keep your baby healthy apart from vaccination?

☐ Exclusive breast feeding for 6 months
☐ Keeping baby warm
☐ Regular hand washing with soap and water
☐ Regular weighing of the baby at the clinic
☐ Others

8a. If others, specify: ____
____
____

For peer review only

For peer review only - http://bmjopen.bmj.com/site/about/guidelines.xhtml

Study Title: The Vaccines Speaking Book Project

SCC:	1598	Version:	2.1

Sponsor & Funder: MRC & Bull City Learning

Primary Care Givers for the Implementation Study: Section B

Record ID: ______________________________

Facility name: ______________________________

Field worker initials: ______________________________

Participants ID: ______________________________

Date of entry: ______________________________

1. How do vaccines work?
   - ☐ Vaccines help a baby or a pregnant woman's body fight illness and diseases
   - ☐ Vaccines work by making security guards in the body called 'anti-bodies'
   - ☐ I don't know
   - ☐ Others

1a. If others specify: ______________________________

2. Why are vaccines given to pregnant women?
   - ☐ Vaccines ensure mother and child are protected against infectious diseases
   - ☐ If the pregnant woman is vaccinated, the baby is also protected for a little while after birth
   - ☐ I don't know
   - ☐ Others

2a. If others, specify: ______________________________

3. What do you do if your child is not well on the day the vaccines need to be given?
   - ☐ I will take my child to the hospital for the doctor or nurse to decide if my child can be immunised
   - ☐ I will wait at home until my child is well
   - ☐ I don't know
   - ☐ Others

3a. If others, specify: ______________________________

4. Do you think it is proper for your child to be given more than one vaccine at a visit?
   - ☐ Yes, different vaccines prevent different infections/diseases

For peer review only

SCC:	1598	Version v 1.2

☐ No, administering more than one vaccine at a time prevents the vaccine from working effectively
☐ I don't know
☐ Others

4a. If others, specify: ____________________

____

____

5. Why do you think your child is sometimes given the same vaccines more than once?

☐ Repeat doses are needed to make the antibodies (body soldiers) stronger
☐ Repeat doses help the baby's body learn to be strong against infections for a long time
☐ I don't know
☐ Others

5a. If others, specify: ____________________

____

____

6. When do I need to consider the adverse event of a vaccine serious?

☐ If my baby has high fever for more than a day
☐ If my baby is sleeping a lot
☐ I don't know
☐ Others

6a. If others, specify: ____________________

____

____

7. What vaccines does your baby receive at each visit? Tick all applicable

7a. At birth

☐ Tuberculosis
☐ Polio
☐ Hepatitis B
☐ Diphtheria
☐ Whooping Cough
☐ Meningitis
☐ Pneumonia
☐ Diarrhoea
☐ Measles
☐ Yellow Fever
☐ I don't know

7b. At 2 Months

☐ Tuberculosis
☐ Polio
☐ Hepatitis B
☐ Diphtheria
☐ Whooping Cough
☐ Meningitis
☐ Pneumonia
☐ Diarrhoea
☐ Measles
☐ Yellow Fever
☐ I don't know

7c. At 3 Months

☐ Tuberculosis
☐ Polio
☐ Hepatitis B

For peer review only - http://bmjopen.bmj.com/site/about/guidelines.xhtml

SCC:	1598	Version v 1.2

☐ Diphtheria
☐ Whooping Cough
☐ Meningitis
☐ Pneumonia
☐ Diarrhoea
☐ Measles
☐ Yellow Fever
☐ I don't know

7d. At 4 Months

☐ Tuberculosis
☐ Polio
☐ Hepatitis B
☐ Diphtheria
☐ Whooping Cough
☐ Meningitis
☐ Pneumonia
☐ Diarrhoea
☐ Measles
☐ Yellow Fever
☐ I don't know

7e. At 9 Months

☐ Tuberculosis
☐ Polio
☐ Hepatitis B
☐ Diphtheria
☐ Whooping Cough
☐ Meningitis
☐ Pneumonia
☐ Diarrhoea
☐ Measles
☐ Yellow Fever
☐ I don't know

7f. At 18 Months

☐ Tuberculosis
☐ Polio
☐ Hepatitis B
☐ Diphtheria
☐ Whooping Cough
☐ Meningitis
☐ Pneumonia
☐ Diarrhoea
☐ Measles
☐ Yellow Fever
☐ I don't know

8. What other ways can you keep your baby healthy apart from vaccination?

☐ Exclusive breast feeding for 6 months
☐ Keeping baby warm
☐ Regular hand washing with soap and water
☐ Regular weighing of the baby at the clinic
☐ Others

For peer review only
For peer review only - http://bmjopen.bmj.com/site/about/guidelines.xhtml

SCC:	1598	Version v 1.2

8a. If others, specify: ____
____
____

For peer review only - http://bmjopen.bmj.com/site/about/guidelines.xhtml

SCC:	1598	Version v 1.2

Primary Care Givers for the Implementation Study: Section C

Record ID: ______________________________

Facility name: ______________________________

Field worker initials: ______________________________

Participants ID: ______________________________

Date of entry: ______________________________

1. Did you find the book easy to use? Yes ☐ No ☐

1a. Can you please give reasons for your response above?

____
____
____
____

2. Did you like the pictures in the book? Yes ☐ No ☐

2a. Can you please give reasons for your response above?

____
____
____
____

3. Could you hear the person talking to you clearly? Yes ☐ No ☐

3a. Can you please give reasons for your response above?

____
____
____
____

4. Did you understand all the information that you were told in the book? Yes ☐ No ☐

4b. Can you please give reasons for your response above?

____
____
____
____

5. Did you find any information in the book useful? Yes ☐ No ☐

For peer review only - http://bmjopen.bmj.com/site/about/guidelines.xhtml

SCC:	1598	Version v 1.2

5a. Can you please give reasons for your response above?

____
____
____
____

5b. If yes, what was the information you found most useful?

____
____
____
____

6. Did you trust the messages in the book? Yes ☐ No ☐

6a. Can you please give reasons for your response above?

____
____
____
____

7. Did you learn new information from the book? Yes ☐ No ☐

7a. Can you please give reasons for your response above?

____
____
____
____

8. How many times did you go through the whole book? 1 ☐ 2-5 ☐ 6-9 ☐ 10 or more times ☐

9. Can you clearly explain the information in the book to your family and friends? Yes ☐ No ☐

9a. Can you please give reasons for your response above?

____
____
____
____

10. Do you think that the information in the book gives you all the information needed to make a decision to immunize your child? Yes ☐ No ☐

For peer review only
For peer review only - http://bmjopen.bmj.com/site/about/guidelines.xhtml

SCC:	1598	Version v 1.2

10b. Can you please give reasons for your response above?

____
____
____
____

11. Were there any problems you had when you were using the book? Yes ☐ No ☐

11b. If Yes, what problem(s) did you experience?

____
____
____
____

12. Do you think the book should include any other information or have any changes? Yes ☐ No ☐

12b. If Yes, what changes would you suggest?

____
____
____
____

13. Did you show the book to anyone in your Family? Yes ☐ No ☐

13a. If yes, how many people ______________

13b. Did you show the book to anyone in your Community? Yes ☐ No ☐

13c. If yes, how many people ______________

13d. Did you show the book to anyone in your Mosque/Church? Yes ☐ No ☐

13e. If yes, how many people ______________

13f. Did you show the book to anyone at work? Yes ☐ No ☐

13g. If yes, how many people ______________

13h. Did you show the book to anyone in your clinic or mosque? Yes ☐ No ☐

13i. If yes, how many people ______________

13j. Can you please specify any other place you shared the book? ______________

14. Do you think members of your family, mosque/church, and community will understand the information in the book if they were given this book to listen to? Yes ☐ No ☐ I don't know ☐

For peer review only - http://bmjopen.bmj.com/site/about/guidelines.xhtml

SCC:	1598	Version v 1.2

14a. Can you please give reasons for your response above?

____
____
____
____

15. At what time do you think that the books should be given to a parent coming to get their child immunized?

- At the time of first visit to the clinic only ☐
- At first few visits to the clinic ☐
- While speaking to the Health care worker ☐
- Before speaking to the Healthcare Worker ☐
- After speaking to the Healthcare worker ☐
- Copy of the book is useful to stay with the parent at home ☐

16. Do you know where the on/off switch is in the book? Yes ☐ No ☐

17. Would you be able to change the battery? Yes ☐ No ☐

18. If you need to change the battery, do you have a screwdriver with which to do so? Yes ☐ No ☐

For peer review only - http://bmjopen.bmj.com/site/about/guidelines.xhtml

For peer review only

Questionnaire for Health Care Workers (HCPs)/ Heads of the Health Centres where the SB invention was implemented

Record ID: ____________________

Facility name: ____________________

Field worker initials: ____________________

Participants ID: ____________________

Date of entry: ____________________

1. Age: ____________
2. Gender: Female ☐ Male ☐
3. Time of working in the current position (years): ____________
4. Do HCWs have enough time to make sure that Primary Care Givers (PCG) completely understand all the information explained to them about immunization? Yes ☐ No ☐

4a. If No, why not?

____
____
____
____

5. Did the book help you in addressing the Primary Care Givers (PCGs) questions and concerns? Yes ☐ No ☐

5a. Can you please give reasons for your response above?

____
____
____
____

6. Did the book make more efficient use of your time and energy? Yes ☐ No ☐

6a. Can you please give reasons for your response above?

____
____
____
____

Version	2.1	Date	8th January 2019

For peer review only - http://bmjopen.bmj.com/site/about/guidelines.xhtml

SCC:	1598	Version v 2.1

7. Did the book increase knowledge of the PCG?
   - Yes ☐
   - No ☐
   - I don't know ☐

7a. Can you please give a reason for your answer above?

____
____
____
____

8. Did the book increase communication between the PCG and you?
   - Yes ☐
   - No ☐

8a. Can you please give a reason for your answer above?

____
____
____
____

9. Was there any other impact of the speaking book on the Primary Care Givers?
   - Yes ☐
   - No ☐

If yes, what was the impact?

____
____
____

9a. Did the Primary Care Givers (PCGs) ask more questions?
   - Yes ☐
   - No ☐

9b. Did you observe timelier Immunisation done of their child by the PCG? `
   - Yes ☐
   - No ☐
   - I don't know ☐

9c. Other observations? (Please mention)

____
____
____

10. When do you think the SB should be given to the PCGs?
   - At the time of first visit to the clinic only ☐
   - At first few visits to the clinic ☐
   - While speaking to the Health care worker ☐
   - Before speaking to the Healthcare Worker ☐
   - After speaking to the Healthcare worker ☐
   - Copy of the book is useful to stay with the parent at home ☐

11. Do you think the book should include any other information or have any changes?
   - Yes ☐
   - No ☐

11a. What information would you suggest to be added?

____
____
____
____

For peer review only - http://bmjopen.bmj.com/site/about/guidelines.xhtml

Supplementary material 5_Themes, sub-themes and quotes

Themes	Subthemes	Supporting quotes
General Challenges at the Clinics, independent of the SBs	Delivering Health Education	"… we normally conduct pre-clinic health talks… we discuss vaccines, and sometimes we choose other topics…. about their health and that of their babies" **(Male, Rural clinic, 18 months in current position)**<br><br>"When we have a large crowd, we also engage them in interpersonal communication while administering the vaccines, we tell them about the vaccines been administered and the possible side effects of the vaccine"**(Male, Rural clinic, 6 months in current position)**
	Attitude of caregiver	"When women come to the clinic, they are always in hurry to go back home to cook or going back to the garden, so most times they are in haste to go home" **(Male, Urban clinic, 5 years in current position)**<br><br>"What we do sometimes is to hold their cards and start weighing, and then when most of the women are around, we start giving the health education" **(Male, Rural clinic, 8 months in current position)**
	Organizational factors	"…most of them [caregivers] come around the same time and the health care worker might not have time to carefully explain in detail to the women … most times the environment is even very noisy" **(Male, Rural clinic, 3years in current position)**<br><br>"…. some of the women will be standing and distracted by the cry of their children and may not even have the patience to listen to what we are saying to them" Adding that: "Interpersonal health education is usually better for us with the crowd (High numbers of caregivers)" **(Female, Rural clinic, 3 years in current position)**
	Low manpower	"… when we started the implementation of the speaking books, I was the only Public Health Officer here without any form of assistance or back up." **(Male, Urban clinic, 5 years in current position)**<br><br>"Sometimes I will be the only one immunizing, weighing and screening so sometimes it's difficult to use the speaking books" **(Male, Urban clinic, 5 years in current position)**
SB Impact	Improved Knowledge	"Most times after listening to the books, we ask them questions on immunisation and they are able to give positive feedback" **(Male, Urban clinic, 18 months in current position)**<br><br>"… I cannot assure you on their [all caregivers attending the immunisation clinic] increase in knowledge compared to women who have personal copies [recruited participants] with them at home." **(Male, Rural clinic, 8months in current position)**
	Efficiency	"…for me it saves time because I just hand over the book to them [Caregiver] and then they listen to everything …. while I concentrate on the work and it makes work faster." **(Female, Rural clinic, 3 years in current position)**

		"… the issue is we have just one copy in the facility which …. is not enough…. they all [Caregiver] have to keep waiting for each other to listen to the books… if we have at least 5 copies it would be better than everyone sharing one copy." **(Male, Urban clinic, 18 months in current position)**
	Informative	"In the past they give so many complains about adverse effects following vaccination……. even blame public health officers that administer the vaccines, they have a better understanding of the side effects after receiving the vaccines and appreciate the information gotten from the book" **(Female, Rural clinic, 3 years in current position)**<br><br>"It has addressed all the details and relevant information that will make mothers want to bring their children for immunization" **(Female, Rural clinic, 3 years in current position)**
	Improved Clinic Attendance	"Mothers are beginning to bring their babies to the clinic unlike before when they will just come to the clinic anytime they like" **(Female, Urban clinic, 10 years in current position)**<br><br>"In the past we used to have low coverages but with the introduction of the speaking books are now seeing more mothers coming" **(Male, Urban clinic, 5 years in current position)**
Outcome of use of the SBs	Enhanced Communication	"The book has made it easier for us to explain information on vaccines to the mothers…" **(Female, Urban clinic, 10 years in current position)**<br><br>"… most of the women don't understand English but with the use of the local languages they understand the information easily……. it has been easier for us to pass across the information because English is not our language, so it is easier for them to understand and easier for us to explain to them" **(Male, Urban clinic, 3 years in current position)**
	Health Promotion Tool	"… we are trying, by recording the audio on our phones and sharing with other health staff because not all health officers have access to the information in this book…. we learnt a lot from it." **(Male, Urban clinic, 3 years in current position)**<br><br>"…… because the book is not loud for all the mothers to hear, I recorded it on my phone, used a Bluetooth device to connect to a speaker for all the mothers to hear" **(Male, Urban clinic, 2 years in current position)**
	Shared responsibility and community ownership	"They share during lunch or when they sit together, they play the book" **(Male, Rural clinic, 8months in current position)**<br><br>"During the immunisation clinic days most of the women who have copies of the speaking books come along with the books and they normally take over the health talks with the use of their speaking books, sometimes I don't even play my own"**(Male, Urban clinic, 5 years in current position)**
Further Suggestions	SB utilization	"I think is better they are given the books at their homes …. in the clinic most times they are in hurry to go home …. at home they will take out time to listen to the book and gain more information compared to listening to the books in the clinic" **(Male, Urban clinic, 5 years in current position)**

		"…. I believe the women will make better use of the speaking books if they are used in the facilities than in their homes ... at the health facility the health workers will be around to guide them, this will ensure a better use of the book…." **(Male, Urban clinic, 18 months in current position)**
	Changes to the SB	"The only problem the speaking book has is that it cannot be used with a large audience because the volume is very low" **(Female, Rural clinic, 3years in current position)**<br><br>"….. it will be good to have a book that is very loud so we don't have to talk, we will just place it at the front of the Clinic, press the buttons and the book will start playing for the women to listen to it" **(Male, Rural clinic, 6 months in current position)**
	Additions to the SB	"…other languages need to be added…. because caregivers who do not understand any of the languages used in the book did not benefit from the information in the book…." **(Male, Urban clinic, 4 years in current position)**<br><br>"…. we can also consider adding information on nutrition and importance of antenatal care by pregnant women" **(Female, Urban clinic, 10 years in current position**

For peer review only

BMJ Open

THE USE OF *A SPEAKING BOOK*® TO ENHANCE VACCINE KNOWLEDGE AMONG CAREGIVERS IN THE GAMBIA

Journal:	*BMJ Open*
Manuscript ID	bmjopen-2020-040507.R2
Article Type:	Original research
Date Submitted by the Author:	02-Jan-2021

Complete List of Authors:	Nkereuwem, Oluwatosin; Medical Research Council Unit The Gambia at the London School of Hygiene and Tropical Medicine, Vaccines and Immunity Theme<br>Kochhar, Sonali; Global Healthcare Consulting, New Delhi, India., Global Health; University of Washington Department of Global Health, Global Health<br>Wariri, Oghenebrume; Medical Research Council Unit The Gambia at the London School of Hygiene and Tropical Medicine, Vaccines and Immunity Theme<br>Johm, Penda; Medical Research Council Unit The Gambia at the London School of Hygiene and Tropical Medicine, Vaccines and Immunity Theme<br>Ceesay, Amie; Medical Research Council Unit The Gambia at the London School of Hygiene and Tropical Medicine, Vaccines & Immunity Theme<br>Kinteh, Mamanding; Expanded Programme on Immunization, Ministry of Health and Social Welfare, The Gambia<br>Kampmann, Beate ; Medical Research Council Unit The Gambia at the London School of Hygiene and Tropical Medicine, Vaccines and Immunity ; London School of Hygiene & Tropical Medicine, The Vaccine Centre

**Primary Subject Heading**:	Epidemiology
Secondary Subject Heading:	Public health, Qualitative research, Global health, Communication
Keywords:	Public health < INFECTIOUS DISEASES, PRIMARY CARE, QUALITATIVE RESEARCH, PREVENTIVE MEDICINE

SCHOLARONE™ Manuscripts

I, the Submitting Author has the right to grant and does grant on behalf of all authors of the Work (as defined in the below author licence), an exclusive licence and/or a non-exclusive licence for contributions from authors who are: i) UK Crown employees; ii) where BMJ has agreed a CC-BY licence shall apply, and/or iii) in accordance with the terms applicable for US Federal Government officers or employees acting as part of their official duties; on a worldwide, perpetual, irrevocable, royalty-free basis to BMJ Publishing Group Ltd ("BMJ") its licensees and where the relevant Journal is co-owned by BMJ to the co-owners of the Journal, to publish the Work in this journal and any other BMJ products and to exploit all rights, as set out in our licence.

The Submitting Author accepts and understands that any supply made under these terms is made by BMJ to the Submitting Author unless you are acting as an employee on behalf of your employer or a postgraduate student of an affiliated institution which is paying any applicable article publishing charge ("APC") for Open Access articles. Where the Submitting Author wishes to make the Work available on an Open Access basis (and intends to pay the relevant APC), the terms of reuse of such Open Access shall be governed by a Creative Commons licence – details of these licences and which Creative Commons licence will apply to this Work are set out in our licence referred to above.

Other than as permitted in any relevant BMJ Author's Self Archiving Policies, I confirm this Work has not been accepted for publication elsewhere, is not being considered for publication elsewhere and does not duplicate material already published. I confirm all authors consent to publication of this Work and authorise the granting of this licence.

For peer review only

THE USE OF A *SPEAKING BOOK*® TO ENHANCE VACCINE KNOWLEDGE AMONG CAREGIVERS IN THE GAMBIA

Oluwatosin Nkereuwem[1], Sonali Kochhar[2,3], Oghenebrume Wariri[1], Penda Johm[1,5], Amie Ceesay[1], Mamanding Kinteh[4], Beate Kampmann[1,5]

[1] Vaccines & Immunity Theme, MRC Unit the Gambia at The London School of Hygiene and Tropical Medicine, The Gambia

[2] Global Healthcare Consulting, New Delhi, India

[3] Department of Global Health, University of Washington, Seattle, USA

[4] Expanded Programme on Immunization, Ministry of Health and Social Welfare, The Gambia

[5] The Vaccine Centre, Faculty of Infectious Diseases, London School of Hygiene and Tropical Medicine, Keppel Street, London, UK

Correspondence to:

Prof. Beate Kampmann, Vaccines and Immunity Theme, Medical Research Council Unit The Gambia at London School of Hygiene and Tropical Medicine, Fajara, P.O. Box 273, Banjul, The Gambia.

Email: bkampmann@mrc.gm

Keywords

Vaccine knowledge; Caregiver knowledge; Communication tools; Multi-media educational tool; Expanded Programme on Immunization (EPI)

For peer review only - http://bmjopen.bmj.com/site/about/guidelines.xhtml

ABSTRACT

Objectives

To measure the usefulness of a *Speaking Book*® as an educational tool for enhancing knowledge, understanding and recall of key vaccine-related information among caregivers in The Gambia, as well as its acceptability and relevance as a health promotion tool for caregivers and healthcare workers.

Design and setting

We developed a multi-media educational tool, the vaccine *Speaking Book*®, which contained pre-recorded information about vaccines provided in The Gambia's Expanded Programme on Immunization (EPI). We then conducted a sequential mixed-methods study on the use of this tool among caregivers and healthcare workers in The Gambia.

Participants

200 caregivers attending primary health care centres in The Gambia for routine immunization services for their infants, and 15 healthcare workers employed to provide immunization services at these clinics.

Outcome measures

We calculated the median knowledge scores on vaccine-related information obtained at baseline, 1-month 3-month follow-up visits. Wilcoxon's matched-pairs signed-rank test was used to compare the difference in the median knowledge scores between baseline and 1-month, and between baseline and 3-month follow-up visits.

Results

Of the 113 caregivers who participated, 104 (92%) completed all three study visits, 108 (95.6%) completed the baseline and 1-month follow-up visits, and 107 (94.7%) completed the baseline and 3-month follow-up visits. The median knowledge score increased from 6.0 (IQR 5.0-7.0) at baseline to 11.0 (IQR 8.0-14.0) at 1-month visit ($P < 0.001$), and 15.0 (IQR 10.0 – 20.0) at 3-month visit ($P < 0.001$). Qualitative results showed high acceptability and enthusiasm for the *Speaking Book*® among both caregivers and healthcare workers. The *Speaking Book*® was widely shared in the community and this facilitated communication with healthcare workers at the primary healthcare centres.

Conclusions

Context and subject specific *Speaking Books*® are a useful communication and educational tool to increase caregiver vaccine knowledge in low-and-middle-income countries.

For peer review only - http://bmjopen.bmj.com/site/about/guidelines.xhtml

Strengths and limitations of this study

- The vaccines *Speaking Book*® (*SB*), a richly illustrated, context-specific, audio-visual, educational tool was specifically designed to complement vaccine information given to caregivers by health care workers in immunization clinics in The Gambia.
- The qualitative and quantitative approach employed in this study explored the impact of the (*SB*) as a health promotion tool, involving both caregivers and healthcare workers.
- A limitation of this study is a possibility of self-selection bias due to the absence of a control group.
- Although we encouraged the study participants to share the *SB* with their family and friends, we did not capture any effects on the overall vaccine knowledge at the community level.
- We did not systematically measure the impact on subsequent uptake of immunization services.

For peer review only

INTRODUCTION

Immunization is one of the most effective public health interventions for the control and prevention of infectious diseases.[1] Unfortunately, global immunization coverage rates remain sub-optimal with approximately 21.8 million infants never completing the recommended immunization schedule.[2, 3]

Several factors may act as barriers to childhood immunization especially in low-and-middle income countries (LMIC) where caregivers often have low literacy levels.[4-7] In addition to the wider concerns of vaccine confidence that have been expressed across the globe, more specific issues include caregivers lack of knowledge about the recommended immunization schedules and alternatives once their infants miss scheduled immunizations, concerns about vaccine safety and adverse events, infants being unwell at the time of the appointment, reluctance to receive multiple vaccinations at the same time, lack of trust in the medical community, being unable to remember the information provided and the date for the next immunisation visit, hesitancy in asking questions which might be perceived as trivial by healthcare workers (HCW), and HCW being too busy or lacking the knowledge or patience to answer the caregivers questions and addressing their concerns.[4, 8-10]

The caregivers' knowledge and overall vaccine knowledge influence their decision to access immunization services for their infants.[8, 11] This decision in turn impacts on the uptake of vaccination services, and consequently, on the morbidity and mortality attributable to vaccine-preventable diseases (VPD).[8, 9] Data from high-income countries (HIC) have shown that improving health awareness and knowledge of caregivers about disease prevention can improve health outcomes, especially among less literate populations.[12] This would be applicable especially in LMIC in sub-Saharan Africa where caregivers have been shown to have low knowledge about immunization and vaccine-related issues.[13]

The Gambian expanded programme on immunization (EPI) programme is considered as highly successful compared to other countries in sub-Saharan Africa. The programme has consistently maintained coverage of the third dose of Diphtheria-Tetanus-Pertussis (DTP3) above 95% and DTP1 to DTP3 dropout rates below 10% since 2005. However, despite these overall high coverages documented over the years, there still exist wide intra-country equity gaps along the lines of caregivers' education and wealth quintile.[14]

Recently, audio-visual tools like the *Speaking Book*® (*SB*) have been developed and used to target HCW and care-seekers in human immunodeficiency virus (HIV) care and mental health services, with studies demonstrating their usefulness in the improvement of knowledge.[12, 15, 16] These studies have also shown that *SB* complements communication between HCW and health care seekers. In a previous study conducted in The Gambia, a clinical trials-specific *SB* was shown to increase the knowledge of study participants, leading to a better informed decision about participation.[12] Therefore, using context-specific, pictorial depictions followed by recorded audio messages targeted at caregivers is

For peer review only - http://bmjopen.bmj.com/site/about/guidelines.xhtml

considered an effective way to deliver messages about vaccines in general, especially in a low literacy setting such as The Gambia.[17]

In this study, we developed the vaccine *SB*, a new *SB* covering routinely recommended maternal and childhood vaccines. We assessed its use as an educational tool for enhancing knowledge, understanding and recall of key vaccine-related information among caregivers in The Gambia, as well as its acceptability and relevance as a health promotion tool for caregivers and HCW.

METHODS

Study design and setting

We conducted a study which used quantitative and qualitative methods to enrol caregivers and their infants attending immunization clinics in 15 purposively selected Primary Health Care facilities (PHCs) across four regions of The Gambia. The PHCs comprised of seven rural and eight urban centres that had not previously participated in vaccine-related research studies or clinical trials. Based on existing records, the Gambia EPI selected immunization facilities with poorer performance compared to expected national outcomes to participate in the study. The project was conducted in close collaboration with the communications department of the Gambia EPI.

Immunization services are provided free-of-charge at all government facilities in The Gambia, and caregivers are encouraged to access the services closest to their home. Immunization-related information should be routinely delivered during immunization clinic days by public health officers (PHO). For the purpose of this study, we defined a caregiver as an adult aged 18 years and above attending the immunisation clinic with an infant, and responsible for that infant's day-to-day care. This included but was not limited to biological parents.

Ethical approval was obtained from The Gambia Government/MRC Joint Ethics Committee (SCC 1598).

Study tool

The prototype of the richly illustrated, audio-visual educational tool called a *Speaking Book* (*SB*) was developed by a United States-based company (https://speakingbooks.com/), with whom we collaborated. The *SB* concept can be adapted for specific purposes, and in our case, we adapted the text, recording and illustrations to vaccines routinely delivered by the Gambia EPI program.

During an iterative pilot phase, we developed a bespoke *SB* version and conducted four separate focus group discussions (FGD) with Gambia EPI programme managers, HCW, and caregivers attending EPI clinics to ensure that the final version of the *SB* reflected the local Gambian context. The final version of the *SB* was an A4-size hard cover book consisting of 16 pages of colourful, culturally sensitive illustrations with short texts written in English and recorded narrations in the two most widely spoken local languages, *Wolof* and *Mandinka.* Each *SB* has a plastic panel with removable

For peer review only - http://bmjopen.bmj.com/site/about/guidelines.xhtml

battery, which hosts a series of push buttons, each corresponding to a specific page in the *SB*. When activated, the push buttons trigger a soundtrack of the text on the relevant page. The soundtrack was narrated by two respected local actors, with the appropriate voice and tonal quality. The language could be selected via a switch button. Figure 1 shows some photographs of the *SB*. (See supplementary material 1)

Data collection methods

Quantitative methods

Following a wide literature search, we designed a structured questionnaire for the quantitative data collection. To assess the construct and content of the data collection tool, we pre-tested it with 25 caregivers and five HCW who provided feedback which was used to refine the wording of the questions and response options. The final version of the questionnaire consisted of five sections capturing the following: i) socio-demographics of the caregiver (19 questions); ii) immunization history and experiences of the caregiver (five questions); iii)socio-demographics of their child(ren) (10 questions); iv) a series of 8 multiple-choice questions with 39 correct choices assessing vaccines and immunization knowledge of the caregiver; and v) a series of 18 open and close-ended questions assessing the experiences of the caregiver on the use of the *SB*. A sample questionnaire is included in supplementary materials 2 and 3.

Qualitative methods

A qualitative approach was used to explore the perception of HCW on the use of the *SB* as a health promotion tool when sharing vaccine information with caregivers during immunisation clinics. We carried out in-depth interviews (IDI) with HCW using 11 general, open-ended and non-leading questions. These included questions which explored the challenges they faced when sharing vaccine information with caregivers, impact of the *SB* tool on their work, outcomes since the utilization of the *SB*, and suggestions on the future use of the tool. The interview guide is in the supplementary material 4.

Participant selection and data collection

All study participants were sensitized on the study procedures and objectives. Participants were eligible for inclusion if they were caregivers with infants below six months of age attending one of the selected PHC centres in The Gambia, had been living at their current address for at least six months, were able to communicate in either *Wolof* or *Mandinka,* and were able to provide written consent. Purposive sampling strategy was used to select caregivers who met the inclusion criteria for enrolment into the study.

During an initial sensitization visit, the research staff educated the caregivers on the aim of the study and gave the caregivers an information sheet and consent form to take home and return on another date. Each caregiver who returned a completed consent form was then invited to participate in a

For peer review only - http://bmjopen.bmj.com/site/about/guidelines.xhtml

baseline visit during which the questionnaire was interviewer-administered to obtain socio-demographic information and to assess their baseline knowledge about childhood and maternal vaccines. Only one attempt was allowed for response to the questions. To assess the knowledge of caregivers, we computed a knowledge score which was calculated by assigning a score of 1 for each correct answer and 0 for each incorrect answer, with a maximum and minimum knowledge score of 39 and 0 respectively. Only sections i) to iv) of the questionnaire were administered during this baseline visit.

Subsequently, the mechanics of the *SB* were explained, and each caregiver was given a copy of the *SB* for use at home. Each PHC head was also given a copy of the *SB* to be used during health education activities in the facilities. Caregivers could listen to the *SB* as many times as they wished during the entire study period and were also encouraged to invite other people to listen along with them. Following receipt of the *SB,* the participants were requested to return to the PHCs for follow-up visits at one month and three months after the baseline visit. During each of these follow-up visit, the participants' understanding, retention, utilisation of key information and experiences using the *SB* were assessed with the same questionnaire used at baseline. Section v) of the questionnaire was also administered during this visit.

At the end of the three months, we conducted in-depth interviews with one representative HCW from each of the participating immunization clinics. During this interview, we assessed their perception of the acceptability, potential efficacy and use of the *SB* as a health promotion tool for HCW delivering immunisation services in The Gambia. Qualitative data were audio-recorded and transcribed by the research team.

Data analysis

The median knowledge scores were calculated at the baseline, 1-month and 3-month follow-up visits. We compared the difference in the median knowledge scores between baseline and one month, and between baseline and three months for the entire cohort and by sub-groups using the Wilcoxon's matched-pairs signed-rank test for non-parametric paired data. The sub-groups used for analysis included region, age, household income and level of education of caregiver. The distribution of the scores are presented in tables showing median scores and inter-quartile ranges (IQR) as appropriate, with significance level set at $P < 0.05$. All quantitative data were entered into Research Data Capture (REDCap) for proper documentation and analyses were performed using Stata version 13 (StataCorp, USA).

For the qualitative analysis, we applied inductive thematic analysis in deriving our themes and sub-themes as described by Braun et al.[18] We used NVivo® (version 12) software to organise the qualitative data during the analysis.

For peer review only - http://bmjopen.bmj.com/site/about/guidelines.xhtml

Patient and public involvement

The public (caregivers and HCW) were involved in the design of the *SB* and the questionnaires used for data collection tool.

RESULTS

Between January and July 2019, we approached 200 eligible caregivers to participate in the study of which a total of 113 were enrolled after exclusion of 45 caregivers due to refusal of consent or absence of permission to participate from family, and 42 caregivers who could not be subsequently contacted on the phone numbers and addresses provided (Figure 2). Of the 113 caregivers, 104 (92%) completed all 3 study visits, 108 (95.6%) completed the baseline and 1-month follow up visits, and 107 (94.7%) completed the baseline and 3-month follow up visits. Reasons for missed follow-up visits were ill-health of the mother, ill-health of infant and change in location.

All consenting caregivers were biological mothers of the infants presenting at the health facility. The mean age of the caregivers was 26.7 years ± 5.95. Most of the caregivers were married (n= 106, 93.8%), Muslim (n= 111, 98.2%), and unemployed (n=91, 80.5%). Over two-thirds of the caregivers reported that they lived within one hour of the immunization clinic, and most women (n=109, 96.5%) had received at least one dose of tetanus toxoid (TT) during their last pregnancy. Although none of the caregivers identified the media as their source of information regarding immunization, 90 (79.6%) households had a functional radio, and about half of them had a television in their homes. Over two-thirds of the caregivers acknowledged that HCW at the immunization clinics were their major source of information on immunization. Table 1 summarises the socio-demographic characteristics of the caregivers.

Table 1: Sociodemographic characteristics of Caregivers enrolled

Variable	N = 113	%
**Age (years)**		
<20	23	20.4
21-30	64	56.6
>30	26	23.0
**Religion**		
Muslim	111	98.2
Christian	2	1.8
**Region**		
Urban	52	46.0
Rural	61	54.0
Marital status		
Unmarried	7	6.2
Married	106	93.8
**Employment status**		

For peer review only - http://bmjopen.bmj.com/site/about/guidelines.xhtml

Employed	22	19.5
Unemployed	91	80.5
**Highest level of education**		
No Education	42	37.2
Primary	11	9.7
Secondary	21	18.6
Higher Secondary	21	18.6
Tertiary	2	1.8
Arabic/French	16	14.2
**Monthly household income**		
Below $50	19	16.8
$50-$100	38	33.6
$100-$200	14	12.4
Above $200	3	2.7
Don't Know	39	34.5
**Presence of working radio in the home**		
Yes	90	79.6
No	23	20.4
**Presence of working television in the home**		
Yes	56	49.6
No	57	50.4
**Reported distance to health facility**		
Less than 30 minutes	41	36.3
30 minutes to one hour	37	32.7
Over 1 hour	13	11.5
Not sure	22	19.5
**Mother's receipt of tetanus toxoid**		
Yes	109	96.5
No	4	3.5
**Usual source of information regarding immunization**		
Health workers	77	68.1
Infant welfare card	6	5.3
Family member or friend	4	3.5
Media	0	0.0
Not sure	26	23.0

Impact of *SB* on knowledge of caregivers

There was a significant increase in the median knowledge score from 6.0 (IQR 5.0-7.0) at baseline to 11.0 (IQR 8.0-14.0) at 1-month visit (P = <0.001), and 15.0 (IQR 10.0-20.0) at 3-month visit (P = <0.001). The highest median knowledge scores at 1-month and 3-month visits were seen among the caregivers above 30 years of age and families with monthly house-hold income above $100 (13.0 and 17.0 respectively). When analysed by subgroups (urban or rural, age of caregivers, household income or highest level of education), there was significant improvement in knowledge scores across all groups, as summarised in Table 2

For peer review only - http://bmjopen.bmj.com/site/about/guidelines.xhtml

Table 2: Median (IQR) of the knowledge scores at baseline, 1-month and 3-month visits*

Variable	N=108	Median baseline knowledge score[a]	Median knowledge score at 1-month[a]	Median difference[a] (1-month – baseline score)	*P*-value[b]	N=107	Median baseline knowledge score[c]	Median knowledge score at 3-months[c]	Median difference[c] (3-month – baseline score)	*P*-value[b]
**All caregivers**	108	6.0 (5.0-7.0)	11.0 (8.0-14.0)	5.0 (2.0-8.0)	<0.0001	107	6.0 (5.0-7.0)	15.0 (10.0-20.0)	8.0 (5.0-14.0)	<0.0001
**Region**										
Rural	57	6.0 (5.0-7.0)	11.0 (8.0-15.0)	4.0 (2.0-9.0)	<0.0001	58	6.0 (5.0-7.0)	15.5 (10.0-21.0)	10.0 (5.0-15.0)	<0.0001
Urban	51	6.0 (4.5-7.0)	11.0 (7.0-14.0)	5.0 (2.0-7.0)	<0.0001	49	6.0 (4.5-7.0)	13.0 (11.0-19.0)	8.0 (5.0-12.0)	<0.0001
**Age (years)**										
<20	23	6.0 (5.0-7.0)	9.0 (8.0-14.0)	4.0 (2.0-7.0)	0.0001	23	6.0 (5.0-7.0)	12.0 (8.0-19.0)	6.0 (2.0-14.0)	<0.0001
21-30	60	6.0 (5.0-7.0)	11.0 (7.0-14.0)	4.0 (2.0-8.0)	<0.0001	61	6.0 (5.0-7.0)	15.0 (11.0-21.0)	8.0 (5.0-14.0)	<0.0001
>30	25	6.0 (4.0-7.0)	13.0 (10.0-16.0)	6.0 (4.0-10.0)	<0.0001	23	6.0 (4.0-7.0)	16.0 (12.0-17.0)	9.0 (6.0-13.0)	<0.0001
**Household income**										
<$50	19	6.0 (5.0-7.0)	11.0 (10.0-14.0)	5.0 (4.0-8.0)	0.0002	18	6.0 (5.0-7.0)	15.5 (13.0-23.0)	9.5 (6.0-17.0)	0.0002
$50-100	38	6.0 (5.0-7.0)	11.0 (8.0-14.0)	5.0 (3.0-7.0)	<0.0001	37	6.0 (5.0-7.0)	14.0 (11.0-20.0)	8.0 (5.0-13.0)	<0.0001
>$100	15	6.0 (5.0-7.0)	12.0 (8.5-15.5)	7.0 (2.5-9.5)	0.0009	13	6.0 (5.0-7.0)	17.0 (14.0-20.0)	10.0 (7.0-13.0)	0.0004
Not sure	36	6.0 (5.0-6.0)	9.0 (7.0-15.0)	3.5 (1.5-9.5)	<0.0001	39	6.0 (5.0-6.0)	12.0 (8.0-20.0)	7.0 (3.0-15.0)	<0.0001
**Level of education**										
None	41	6.0 (5.0-7.0)	11.0 (8.0-15.0)	5.0 (2.0-10.0)	<0.0001	41	6.0 (5.0-7.0)	15.0 (11.0-21.0)	9.0 (5.0-15.0)	<0.0001
Primary	11	5.0 (4.0-6.0)	10.5 (8.0-15.0)	5.5 (4.0-9.0)	0.0057	11	5.0 (4.0-6.0)	16.5 (12.0-22.0)	12.0 (8.0-17.0)	0.005
Secondary	19	6.0 (5.0-7.0)	11.0 (9.0-12.0)	5.0 (3.0-7.0)	0.0001	19	6.0 (5.0-7.0)	13.0 (10.0-16.5)	7.5 (5.0-10.0)	0.0001
Higher sec./tertiary	22	6.0 (5.0-7.0)	10.5 (8.0-13.0)	4.5 (2.0-8.0)	0.0002	21	6.0 (5.0-7.0)	16.5 (10.0-23.0)	11.0 (5.0-16.0)	0.0001
Arabic/French	15	6.5 (6.0-7.0)	10.0 (7.0-15.0)	3.0 (1.5-8.5)	0.0048	15	6.0 (5.0-7.0)	13.0 (8.0-17.5)	6.0 (2.5-11.5)	0.0009

[a] Median baseline knowledge score and median difference calculated using the same participants who were present at both baseline visit and 1-month follow-up visit
[b] Wilcoxon signed rank test P-values
[c] Median baseline knowledge score and median difference calculated using the same participants who were present at both baseline visit and 3-month follow-up visit
** Since median (IQR) of the differences are reported, these might not match the difference of the medians at each time point.*

For peer review only - http://bmjopen.bmj.com/site/about/guidelines.xhtml

Opinions of the caregivers

In general, the *SB* was very well received by the caregivers, and their opinions are summarised in Table 3. The caregivers found the *SB* increasingly easy to use 103/108 (95.4%) at 1 month, versus 107/107 (100%) at 3 months respectively), and most of them stated that they understood all the information in the book 100/108 (92.6%) at 1 month vs 98/107; 91.6% at 3 months. Many participants also shared the *SB* in their communities and expressed trust in the information provided. The survey results also showed that using the book was easy, with only 10 (9.3%) and 7 (6.5%) caregivers reporting some problems at 1-month and 3-month visits respectively.

Table 3: Opinions of caregivers about the *Speaking Book®*

Questions		1-month visit n=108	(%)	3-month visit n=107	(%)
Did you find the book easy to use?	Yes	103	95.4	107	100
	No	5	4.6	0	0
Did you like the pictures in the book	Yes	108	100	107	100
	No	0		0	0
Could you hear the person talking to you clearly?	Yes	108	100	107	100
	No	0		0	0
Did you understand all the information that you were told in the book	Yes	100	92.6	98	91.6
	No	8	7.4	9	8.4
Did you find the information in the book useful	Yes	107	99.1	106	99.1
	No	1	0.9	1	0.9
Did you trust the messages in the book?	Yes	108	100	106	99.1
	No	0	0	1	0.9
Did you learn new information from the book?	Yes	102	94.4	105	98.1
	No	6	5.6	2	1.9
How many times did you go through the whole book?	once	4	3.7	5	4.7
	2-5 times	40	37.0	33	30.8
	6-9 times	10	9.3	3	2.8
	≥10 times	54	50.0	66	61.7
Can you clearly explain the information in the book to your family and friends?	Yes	107	99.1	102	95.3
	No	1	1.0	5	4.7
Do you think that the information in the book gives you all the information needed to decide to immunize your child?	Yes	108	100	106	99.1
	No	0	0	1	0.9
Were there any problems you had when you were using the book?	Yes	10	9.3	7	6.5
	No	98	90.7	100	93.5
Do you think the book should include any other information or have any changes?	Yes	30	27.8	20	18.7
	No	78	72.2	87	81.3
Did you show the book to anyone in your Family?	Yes	107	99.1	106	99.1
	No	1	1.0	1	0.9
Did you show the book to anyone in your Community?	Yes	87	80.6	96	89.7
	No	17	15.7	11	10.3
	No Response	4	3.7	0	0.0
Did you show the book to anyone in your Mosque/Church?	Yes	4	3.7	2	1.9
	No	104	96.3	104	97.2
	No Response	0	0	1	0.9
Did you show the book to anyone at work?	Yes	21	19.4	16	15.0

For peer review only - http://bmjopen.bmj.com/site/about/guidelines.xhtml

	No	86	79.6	91	85.0
	No Response	1	0.9	0	0
Did you show the book to anyone at the clinic or hospital?	Yes	10	9.3	14	13.1
	No	95	88.0	90	84.1
	No Response	3	2.8	3	2.8
Do you think members of your family, mosque/church, and community will understand the information in the book if they were given this book to listen to?	Yes	106	98.1	100	93.5
	No	0	0.0	1	0.9
	I don't know	2	1.9	6	5.6
At what time do you think that the books should be given to a parent coming to get their child immunized?	At the time of first visit to the clinic only	82	75.9	102	95.3
	At first few visits to the clinic	11	10.2	5	4.7
	While speaking to the HCW	1	0.9	0	0
	Before speaking to the HCW	2	1.9	0	0
	After speaking to the HCW	10	9.3	0	0
	No Response	2	1.9	0	0
Do you know where the on/off switch is in the book?	Yes	108	100	107	100
	No	0	0.0	0	0
Would you be able to change the battery?	Yes	95	88.0	106	99.1
	No	11	10.2	1	0.9
	No Response	2	1.9	0	0
If you need to change the battery, do you have a screwdriver with which to do so?	Yes	83	76.9	97	90.7
	No	25	23.1	10	9.3

Themes from qualitative questions posed to Caregivers in questionnaire

In addition to the questions posed to caregivers on their experiences using the *SB* from the open-ended part of the questionnaire, a majority of caregivers (n=98, 90.7% and n=100, 93.5%) reported no problems while using the speaking books at 1- and 3-months post visit respectively. Reoccurring themes with supporting quotes are presented below:

- Information appropriateness

"I trust the messages in the book because the nurses tell me the same information as stated in the book." [32 years old, French language certificate, urban dweller]

- Clear presentation

"*… because the book is written in English… and also clearly translated in two local languages.*" [31 years old, Tertiary Education, urban dweller]

"*…the pictures make me believe the messages.*" [30 years old, No Education, rural dweller]

- Evidence of vaccine effectiveness

"*… I trust the messages because some diseases we used to see…. [giving examples] have now drastically reduce.*" [37 years old, Secondary (Grade 7–9), rural dweller]

For peer review only - http://bmjopen.bmj.com/site/about/guidelines.xhtml
For peer review only

Of the small number of caregivers who reported problems while using the speaking books at 1-month (n=10, 9.3%) and 3-month (n=7, 6.5%) follow-up visits respectively, the reoccurring themes with supporting quotes are presented below:

- Understanding information

"*... I found it difficult understanding the book at first, but after some time I began to understand better.*" [32 years old, Secondary (Grade 7–9), urban dweller]

"*... because I am not educated, I found the book difficult to learn despite the audio… I began to understand with the help of some students who read and explained to me.*" [25 years old, Secondary (Grade 7–9), urban dweller]

- Sense of responsibility

"... Initially I was afraid the book would go bad in my care but with time I was not afraid anymore." [27 years old, No education, rural dweller]

- Challenges with the batteries

"... The battery died and then I had to change it." [25 years old, Tertiary (University education), urban dweller]

Themes and sub-themes from in-depth interviews with HCW

In-depth interviews were conducted with 14/15 (93.3%) HCW at the immunization clinics. The mean age of the HCW interviewed was 33.4 years, and they were predominantly female (64.3%). One of the HCW could not be interviewed as they were absent from work due to ill-health. Themes and sub-themes are summarized in Figure 3. See also supplementary material 5.

Theme 1: General Challenges at the Clinics, independent of the SBs

Sub-theme 1.1: Delivering Health Education

From our discussions, we found out that HCW were expected to deliver 'health talks' in the clinics before immunization sessions. These health talks would typically include information about the vaccines their infants would receive that day, address the general expectations of caregivers, and remind the caregivers when to return for their next appointment. In some cases, HCW would add points that are important for the health of the babies like regular weighing, nutrition, among others.

- "*... we normally conduct pre-clinic health talks… We discuss vaccines, and sometimes we choose other topics… about their health and that of their babies.*" (Male, Rural clinic, 18 months in current position)

Discussions with HCW suggested that these vaccine clinic health talks are not standardised, and the content and quality vary between locations and situations. Immunization activities in facilities were concentrated in the morning hours of the day. While HCW planned pre-clinic health talks at the beginning of every immunization clinic, several of them ended up only briefly summarizing the plan

For peer review only - http://bmjopen.bmj.com/site/about/guidelines.xhtml

before vaccinating, while some resorted to giving caregivers briefs while administering vaccines at the immunization table. Others did not bother to give the health talk at all due to time constraints. This was confirmed by one of the HCW who said:

- *"When we have a large crowd, we also engage them in interpersonal communication while administering the vaccines, we tell them about the vaccines been administered and the possible side effects of the vaccine."* (Male, Rural clinic, 6 months in current position)

Sub-theme 1.2: Attitude of caregiver

Several HCW expressed dissatisfaction regarding the attitude of caregivers. According to them, some caregivers did not show up on their visit appointments, others came late, while others were impatient and always in a hurry to go home. HCW perceived caregivers to value domestic chores and activities more than getting the best out of the immunization clinic visit. This was confirmed by a HCW who said:

- *"When women come to the clinic, they are always in hurry to go back home to cook or going back to the garden, so most times they are in haste to go home."* (Male, Urban clinic, 5 years in current position)

In ensuring caregivers sat and listened to the vaccine health education, HCW developed various means of ensuring they stayed to receive the information. According to one of the HCW:

"What we do sometimes is to hold their cards and start weighing, and then when most of the women are around, we start giving the health education." (Male, Rural clinic, 8 months in current position)

Sub-theme 1.3: Organizational factors

Our discussions with HCW revealed that although clinic schedules were planned in advance, there were a limited number of immunization clinics in a month, especially in rural settings. According to a few HCW, overcrowding and congestion during immunization clinics was common as many of the caregivers visited at the same time on the limited number of days allotted to immunization services.

- *"...most of them [caregivers] come around the same time and the health care worker might not have time to carefully explain in detail to the women ... most times the environment is even very noisy."* (Male, Rural clinic, 3 years in current position)

The clinic setting was sometimes found to be very noisy and chaotic. According to one of the HCW,

- *".... some of the women will be standing and distracted by the cry of their children and may not even have the patience to listen to what we are saying to them." Adding that: "Interpersonal health education is usually better for us with the crowd (large numbers of caregivers)."* (Female, Rural clinic, 3 years in current position)

Our discussions revealed that these challenges cut across facilities in both the urban and rural centres. The HCW further emphasised the importance of the *SB* in filling the gaps of improving the knowledge of caregiver.

For peer review only - http://bmjopen.bmj.com/site/about/guidelines.xhtml

Sub-theme 1.4: Low manpower

Findings revealed that some facilities did not have adequate HCW/Public Health Officers in the clinic to deliver immunization services. Staff shortages were stressful for both HCW and Caregiver, resulting in long waiting time, poor service delivery and dissatisfaction. This was confirmed by one of the HCW who said:

- *"... when we started the implementation of the speaking books, I was the only Public Health Officer here without any form of assistance or back up."* (Male, Urban clinic, 5 years in current position)

Another HCW said:

- *"Sometimes I will be the only one immunizing, weighing and screening so sometimes it's difficult to use the speaking books."* (Male, Urban clinic, 5 years in current position)

Staff shortages are stressful for both HCW and caregivers, resulting in long waiting time, poor service delivery and dissatisfaction.

Theme 2: *SB* Impact

Sub-theme 2.1: Improved Knowledge

HCW revealed that they observed increased knowledge among caregivers as a result of the utilization of the *SB*. They added that the *SB* addresses vaccines issues adequately.

- *"Most times after listening to the books, we ask them questions on immunisation and they are able to give positive feedback."* (Male, Urban clinic, 18 months in current position)
- *"... I cannot assure you on their [all caregivers attending the immunisation clinic] increase in knowledge compared to women who have personal copies [recruited participants] with them at home*." (Male, Rural clinic, 8 months in current position)

Sub-theme 2.2: Efficiency

Over the course of the discussion, HCW expressed ways the *SB* has increased productivity with their work. Some of the HCW emphasized the importance of the local languages used in the book. The *SB* was judged to be a self-explanatory tool which most of the caregiver could use with little or no assistance.

- *"... for me it saves time because I just hand over the book to them (caregivers) and then they listen to everything... while I concentrate on the work and it makes work faster."* (Female, Rural clinic, 3 years in current position)

Some of the HCW expressed their desire have more copies of the *SB* in the facilities to further increase productivity with their work.

- *"... the issue is we have just one copy in the facility which... is not enough... they (caregivers) all have to keep waiting for each other to listen to the books... if we have at least five copies it would be better than everyone sharing one copy."* (Male, Urban clinic, 18 months in current position)

For peer review only - http://bmjopen.bmj.com/site/about/guidelines.xhtml

Sub-theme 2.3: Informative

Some of the HCW described the *SB* as an informative tool which served the needs of caregivers and HCW. The content was able to address most of the concerns of caregiver and presented vaccine information in a simple way that could be easily understood.

- *"In the past they give so many complains about adverse effects following vaccination……. even blame public health officers that administer the vaccines, they have a better understanding of the side effects after receiving the vaccines and appreciate the information gotten from the book.*" (Female, Rural clinic, 3 years in current position)
- *"It has addressed all the details and relevant information that will make mothers want to bring their children for immunization."* (Female, Rural clinic, 3 years in current position)

Sub-theme 2.4: Improved Clinic Attendance

HCW repeatedly highlighted enhanced clinic attendance by caregiver since the introduction of the *SB*, especially among those with personal copies of the *SB*. Caregivers appeared more motivated and dedicated to the immunization schedule. Some HCW suggested that the *SB* had resulted in increased uptake of vaccination, helping them to meet their coverage targets.

- *"Mothers are beginning to bring their babies to the clinic unlike before when they will just come to the clinic anytime they like.*" (Female, Urban clinic, 10 years in current position)
- *"In the past we used to have low coverages but with the introduction of the speaking books are now seeing more mothers coming.*" (Male, Urban clinic, 5 years in current position)

Theme 3: Outcome of use of the *SB*s

Sub-theme 3.1: Enhanced Communication

Discussions focusing on how the *SB* had improved communication between HCW and caregiver revealed that the two *SB* local languages had aided the sharing of vaccine information. Some HCW expressed their struggle in explaining vaccine preventable diseases in way that could be understood by mothers prior to the introduction of the *SB*.

- *"The book has made it easier for us to explain information on vaccines to the mothers…"* (Female, Urban clinic, 10 years in current position)
- *"… most of the women don't understand English but with the use of the local languages they understand the information easily… It has been easier for us to pass across the information because English is not our language, so it is easier for them to understand and easier for us to explain to them."* (Male, Urban clinic, 3 years in current position)

Sub-theme 3.2: Health Promotion Tool

As the discussions continued, some of the HCW highlighted the importance of the *SB* to them and their colleagues in other facilities. They considered the *SB* an important tool for HCW in general. They discussed the importance of sharing the information in the *SB* with HCW in other facilities, including through recoding the audio on a phone and sharing with colleagues on social media. One HCW said:

For peer review only - http://bmjopen.bmj.com/site/about/guidelines.xhtml

- *"… we are trying, by recording the audio on our phones and sharing with other health staff because not all health officers have access to the information in this book… We learnt a lot from it."* (Male, Urban clinic, 3 years in current position)

Another HCW expressing his own unique way of making use of the *SB* said:

- *"Because the book is not loud for all the mothers to hear, I recorded it on my phone, used a Bluetooth device to connect to a speaker for all the mothers to hear."* (Male, Urban clinic, 2 years in current position)

Sub-theme 3.3: Shared responsibility and community ownership

According to the HCW, some of the women shared information from the *SB* in the community and some women had been eager to take the responsibility of health talks during immunization clinics with their peers. HCW considered this as a way of spreading vaccine-related information and facilitating their own work.

- *"They share during lunch or when they sit together, they play the book."* (Male, Rural clinic, 8 months in current position)
- *"During the immunisation clinic days most of the women who have copies of the speaking books come along with the books and they normally take over the health talks with the use of their speaking books, sometimes I don't even play my own."* (Male, Urban clinic, 5 years in current position)

Further discussion revealed that caregivers made use of the *SB* in family and community gathering.

- "… *in the communities, mothers and caregivers look up to those who have benefitted from the study they are like mentors to other mothers and caregivers in the community where they live."* (Male, Rural clinic, 3 years in current position)

Theme 4: Further Suggestions

Sub-theme 4.1: SB utilization

The opinion of HCW regarding their preferences on the best setting for the utilization of *SB* varied. Some HCW did not make use of the *SB* during very busy clinics but instead preferred to use it when the clinic was quieter. Some suggested the *SB* to be more useful in homes with caregiver as they are able to go through the book at their own convenient time with the added advantage of sharing the *SB* with people in the communities who may not come to the clinic. They believed this would foster transfer of knowledge to the community members.

- *"I think is better they are given the books at their homes… in the clinic most times they are in hurry to go home… at home they will take out time to listen to the book and gain more information compared to listening to the books in the clinic."* (Male, Urban clinic, 5 years in current position)
- *"…. I believe the women will make better use of the speaking books if they are used in the facilities than in their homes ... at the health facility the health workers will be around to guide them, this will ensure a better use of the book…"* (Male, Urban clinic, 18 months in current position)

For peer review only - http://bmjopen.bmj.com/site/about/guidelines.xhtml

Sub-theme 4.2: Changes to the SB

The low sound volume of the *SB* remained a consistent challenge expressed by the HCW during the discussions; some even suggested modifying the *SB* so it could be connected to external devices that could amplify the volume.

- *"The only problem the speaking book has is that it cannot be used with a large audience because the volume is very low."* (Female, Rural clinic, 3 years in current position)
- *"..... it will be good to have a book that is very loud so we don't have to talk, we will just place it at the front of the Clinic, press the buttons and the book will start playing for the women to listen to it."* (Male, Rural clinic, 6 months in current position)

Sub-theme 4.3: Additions to the SB

Constraints with optimal utilization of the *SB* were linked to the use of only two languages in the audio translation of the book. In addition to the challenges faced in recruiting caregivers for this study, HCW further suggested the need to incorporate additional languages to make use of the book with more caregivers of different language backgrounds.

- *"... other languages need to be added.... because caregivers who do not understand any of the languages used in the book did not benefit from the information in the book..."* (Male, Urban clinic, 4 years in current position)

Some HCW suggested other health topics which could be added to the vaccines *SB*, such as nutrition and maternal health.

- *".... we can also consider adding information on nutrition and importance of antenatal care by pregnant women." (Female, Urban clinic, 10 years in current position)*

DISCUSSION

In this study, we assessed the impact of a bespoke, multi-media educational tool, the vaccine *Speaking Book*®, on vaccine-related knowledge of caregivers as well as on communication between HCW and caregivers in The Gambia. We distributed this tool to 113 caregivers visiting PHC for routine immunization services and evaluated changes in vaccine knowledge over a three-month period.

We saw significant improvement in the knowledge scores with the median scores nearly doubling at 1-month and nearly tripling at 3-month follow-up visits, compared to the baseline scores, independent of region of the country (urban or rural), age of caregiver, household income or highest level of education. The *SB* was given to the caregivers to take home, thus giving them several opportunities to listen to and understand the information in the book, which might have contributed to the ongoing improvement over the 3-month period.

Study participants discussed and shared content of the *SB* extensively within their family, community, and social groups, using a variety of platforms such as peer group meetings, community and religious gatherings. This is likely to lead to a significant and possibly measurable multiplier effect. Our results

For peer review only - http://bmjopen.bmj.com/site/about/guidelines.xhtml

For peer review only

support the usefulness of the *SB* beyond individuals' knowledge gain and in both the health facility and community setting. Our findings of "added value" are similar to observations by other investigators who used a similar tool.[17, 19] One study in South Africa reflected the use of a *SB* tool as significant in increasing knowledge on Clinal trials among recruited study participants.[17] Another study reflected the effectiveness of the tool in increasing knowledge of biobanking and genetics among a group of non-academic university staff in South Africa. This qualitative experimental study reported a significant increase overall knowledge score, similar to our findings, this increase in knowledge score did not occur for all questions.[17, 19]

Our results support the importance of providing targeted, vaccine-related education to caregivers and communities as an effective and practical strategy to improve vaccine-related knowledge in low literacy settings such as The Gambia. [20, 21]

Our data is comparable to findings from a previous study conducted by our team in The Gambia[22] where the use of the *SB* equally resulted in significant improvement in knowledge gained among study participants. However, the previous speaking book was directed at providing information about a specific clinical trial of pneumococcal vaccines, not the EPI program and vaccine-related knowledge in general. It was also conducted in a community with previous exposure to vaccine research, a setting we deliberately avoided in the project presented here as it is likely to lead to significant bias. Illustrations in the previous *SB* had also not been adapted to the local Gambia context. During the stakeholder engagement for our study, we received feedback that led to specific alterations with regards to dress code for example, and to derive a *SB* that reflects local communities was an important message received.

While the *SB* was able to significantly improve the vaccine-related knowledge, a knowledge gap remained: although we reported significant improvements in the mean knowledge scores from a very low baseline, the highest score obtained was 19.0, still 20 points below the maximum possible score of 39. It is possible that the detailed knowledge needed to obtain these remaining points is more difficult to retain compared to some other answers and we wondered if there was an educational bias. The subgroup of caregivers with tertiary education did not achieve significantly higher scores, maybe because they already had a higher baseline score. We are unable to draw firmer conclusions about the impact of higher education, since there were only two participants in this group. It is entirely possible that the knowledge score developed was overambitious.

A majority of the caregivers reported that HCW were their usual source of information about vaccines. Hence further training and retraining the HCW to deliver the additional information to the caregivers could significantly contribute to reducing the remaining knowledge gap.[23] In a study similar to ours, the *SB* was used regularly for health promotion in health posts, home visits and local gatherings and HCW referred to the *SB* as a "job aid".[15] They carried out observations, structured

For peer review only - http://bmjopen.bmj.com/site/about/guidelines.xhtml

interviews and focus group discussions with HCW within a selected region of Ethiopia, this informed their reports on the contribution, appropriateness, and challenges of the *SB*.[15]

Most caregivers had listened to the *SB* multiple times and suggested that it would be important for mothers to receive a copy of the *SB* at their first clinic visit, especially while pregnant or when their babies are newly born. Studies have reported distribution of the *SB* to study participants who have either had to opportunity to spend days with the tool, use the tool at workstations or take the tool home as their personal property.[15, 22] Trust in the messages provided by the book was almost unanimous and the illustrations, which were adapted to the local context, were approved by all participants. The initial stakeholder consultation in the design phase of the book was therefore very important. We highly recommend to involve the local communities in the design of such tools, as their identification with the visual materials is likely to increase its acceptancy.[12, 16]

Results obtained from the HCW interviews demonstrated that the *SB* had empowered them to answer concerns and questions of caregivers, emphasising the importance of the local language translations which presented vaccine information in a simple, easy to understand manner. HCW reported increase in productivity and improved clinic attendance and considered the *SB* as an important health promotion tool for both HCW and caregivers in the Gambia. It was evident that the *SB* had a positive impact on their day-to-day practice, and its content was well received, which facilitated communication on vaccines.

Vaccine confidence remains high in countries like The Gambia, but in the overall context of increasing vaccine hesitancy worldwide, tools such as the *SB* or similar approaches tailored to local context might add significant value to engage individuals and communities in communication about the personal and societal value of vaccination. They could be an important counter-measure to vaccine hesitancy.[24-26] It is interesting to note that none of the participants reported obtaining their vaccine-related information from the media, unlike in many high-income countries.

We acknowledge the lack of a control group in our study, but this was beyond our means in this project. We agree that the inclusion of a control group not receiving the *SB* would have been desirable but funding restrictions did not allow us to implement this design as we needed to make sure we had a large enough sample of caregivers who actually received the *SB* and were also representative of the country. This included urban and rural areas and a different set up of health clinics. Further evaluations could be carried out in a properly designed and powered trial in the future and we believe that our study provides useful data for the conduct of such a study.

Overall vaccine literacy is more complex than "just" being knowledgeable about schedules and target diseases of the vaccines provided in the EPI schedule, which was the focus of most of our knowledge

For peer review only

For peer review only - http://bmjopen.bmj.com/site/about/guidelines.xhtml

test. It encompasses understanding, appraisal and application of vaccine-related information services, additional components not captured in this project.

Further research will now be required to quantify any potential effect on vaccine uptake at the health center level and measure cost-effectiveness. In discussions with the Gambian EPI program, both are expressed areas of interest. The *SB* used in this study was only recorded in two of the locally spoken languages (*Wolof* and *Mandinka*) in The Gambia and therefore, excluded caregivers who did not speak or understand either of these languages. We were asked to produce books in additional local languages in the future as well as design books for additional topics, such as pregnancy and nutrition.

While useful and possibly a first step to behaviour change, the intervention (i.e. the *SB*) can of course not address multiple other barriers families face in getting their children vaccinated or the health system barriers' such as lack of staff, identified by the HCW.

CONCLUSION

In conclusion, the vaccine *Speaking Book*® is a health education tool that resulted in significant increase in vaccine-related knowledge of caregivers, irrespective of their age, location, household income or educational level. It also served as a valuable tool for HCW in their interactions with caregivers and might lead to increased uptake of immunization services.

Contributors

BK and SK conceptualised and designed the study. ON, OW and AC were involved in data collection. and analysed the data. ON wrote the first draft; BK, SK, OW, PJ and MK provided critical feedback and helped shape the research, analysis and manuscript. All the authors revised the work for important intellectual content and contributed to the final draft and finally approved it to be published.

Acknowledgements

We thank the field assistants for detailed data collection and transcription from the local languages to English for data analysis. We thank Alexander Jarde for statistical support in the analysis of the study data. We also thank the heads of the participating health facilities and members of staff for granting us access to the EPI clinics and their support throughout the project.

Competing interests

None.

Data sharing statement

The data sets generated and analysed during this study are available from the corresponding author upon reasonable request.

Funding

For peer review only

This study was funded by a project grant from Bull City Learning. BK is supported by funding from the UKRI (MC_UP_A900/1122). The Vaccines & Immunity research led by BK is jointly funded by the UK Medical Research Council (MRC) and the UK Department for International Development (DFID) under the MRC/DFID Concordat agreement and is also part of the EDCTP2 programme supported by the European Union.

For peer review only

For peer review only - http://bmjopen.bmj.com/site/about/guidelines.xhtml

REFERENCES

1. Ventola CL. Immunization in the United States: Recommendations, Barriers, and Measures to Improve Compliance: Part 1: Childhood Vaccinations. P t. 2016 Jul;41(7):426-36
2. Negussie A, Kassahun W, Assegid S, Hagan AK. Factors associated with incomplete childhood immunization in Arbegona district, southern Ethiopia: a case--control study. BMC Public Health. 2016 Jan 12;16:27
3. Nnaji CA, Owoyemi AJ, Amaechi UA, Wiyeh AB, Ndwandwe DE, Wiysonge CS. Taking stock of global immunisation coverage progress: the gains, the losses and the journey ahead. Int Health. 2020 Jan 11
4. Esposito S, Principi N, Cornaglia G. Barriers to the vaccination of children and adolescents and possible solutions. Clin Microbiol Infect. 2014 May;20 Suppl 5:25-31
5. Sato R, Fintan B. Fear, knowledge, and vaccination behaviors among women in Northern Nigeria. Hum Vaccin Immunother. 2020 Feb 21:1-11
6. Ianni A, Tedeschi R, Marchetti A, Basso D, Virgili R, Piredda M, et al. [The role of nurses in health education about vaccines: analysis of style and communication models of institutional vaccination campaigns]. Ig Sanita Pubbl. 2019 Sep-Oct;75(5):355-69
7. Gil Cuesta J, Whitehouse K, Kaba S, Nanan-N'Zeth K, Haba B, Bachy C, et al. 'When you welcome well, you vaccinate well': a qualitative study on improving vaccination coverage in urban settings in Conakry, Republic of Guinea. Int Health. 2020 Jan 13
8. Smith LE, Webster RK, Weinman J, Amlôt R, Yiend J, Rubin GJ. Psychological factors associated with uptake of the childhood influenza vaccine and perception of post-vaccination side-effects: A cross-sectional survey in England. Vaccine. 2017 Apr 4;35(15):1936-45
9. Owais A, Hanif B, Siddiqui AR, Agha A, Zaidi AK. Does improving maternal knowledge of vaccines impact infant immunization rates? A community-based randomized-controlled trial in Karachi, Pakistan. BMC Public Health. 2011 Apr 17;11:239
10. Guzman-Holst A, DeAntonio R, Prado-Cohrs D, Juliao P. Barriers to vaccination in Latin America: A systematic literature review. Vaccine. 2020 Jan 16;38(3):470-81
11. Damnjanović K, Graeber J, Ilić S, Lam WY, Lep Ž, Morales S, et al. Parental Decision-Making on Childhood Vaccination. Front Psychol. 2018;9:735
12. Castelnuovo B, Newell K, Manabe YC, Robertson G. Multi-Media Educational Tool Increases Knowledge of Clinical Trials in Uganda. J Clin Res Bioeth. 2014 Jan 2;5:165
13. Tefera YA, Wagner AL, Mekonen EB, Carlson BF, Boulton ML. Predictors and Barriers to Full Vaccination among Children in Ethiopia. Vaccines (Basel). 2018 Apr 10;6(2)
14. Wariri O, Edem B, Nkereuwem E, Nkereuwem OO, Umeh G, Clark E, et al. Tracking coverage, dropout and multidimensional equity gaps in immunisation systems in West Africa, 2000-2017. BMJ Glob Health. 2019;4(5):e001713

For peer review only - http://bmjopen.bmj.com/site/about/guidelines.xhtml

15. Endale E, Tesfaye S. Rapid Qualitative Assessment of Maternal and Newborn Health Care (MNHC) Speaking Book in Two Districts in the Amhara Region, Ethiopia. UNICEF 2012.
16. Afolabi MO, McGrath N, D'Alessandro U, Kampmann B, Imoukhuede EB, Ravinetto RM, et al. A multimedia consent tool for research participants in the Gambia: a randomized controlled trial. Bull World Health Organ. 2015 May 1;93(5):320-8a
17. Dhai A, Etheredge H, Cleaton-Jones P. A pilot study evaluating an intervention designed to raise awareness of clinical trials among potential participants in the developing world. J Med Ethics. 2010 Apr;36(4):238-42
18. Braun V, Clarke V. Using thematic analysis in psychology. Qual Res Psychol. 2006;3(2):77-101
19. Bedeker A, Anderson D, Lose T, Mgwatyu Y, Luus R, Blignaut R, et al. Understanding biobanking: An assessment of the public engagement speaking book intervention Biobanking and Me. South African J Bioeth Law. 2019;12(2):87
20. Masters NB, Tefera YA, Wagner AL, Boulton ML. Vaccine hesitancy among caregivers and association with childhood vaccination timeliness in Addis Ababa, Ethiopia. Hum Vaccin Immunother. 2018;14(10):2340-7
21. Oku A, Oyo-Ita A, Glenton C, Fretheim A, Ames H, Muloliwa A, et al. Perceptions and experiences of childhood vaccination communication strategies among caregivers and health workers in Nigeria: A qualitative study. PLoS One. 2017;12(11):e0186733
22. Mboizi RB, Afolabi MO, Okoye M, Kampmann B, Roca A, Idoko OT. Recall and decay of consent information among parents of infants participating in a randomized controlled clinical trial using an audio-visual tool in The Gambia. Hum Vaccin Immunother. 2017 Sep 2;13(9):2185-91
23. Arogundade L, Akinwumi T, Molemodile S, Nwaononiwu E, Ezika J, Yau I, et al. Lessons from a training needs assessment to strengthen the capacity of routine immunization service providers in Nigeria. BMC Health Serv Res. 2019 Sep 14;19(1):664
24. Dubé E, Gagnon D, MacDonald NE. Strategies intended to address vaccine hesitancy: Review of published reviews. Vaccine. 2015 Aug 14;33(34):4191-203
25. Wellcome. World survey reveals people trust experts but want to know more about science [cited 2020 Feb 5]. Available from: https://wellcome.ac.uk/news/world-survey-reveals-people-trust-experts-want-know-more-about-science.
26. World Health Organization. Improving vaccination demand and addressing hesitancy [cited 2020 Feb 5]. Available from: https://www.who.int/immunization/programmes_systems/vaccine_hesitancy/en/.

For peer review only - http://bmjopen.bmj.com/site/about/guidelines.xhtml

FIGURE LEGENDS

Figure 1: Photo of the *Speaking Book*®

Figure 2: Flow chart of participant recruitment process

Figure 3: Themes and sub-themes from in-depth interviews with healthcare workers

For peer review only

Figures 1

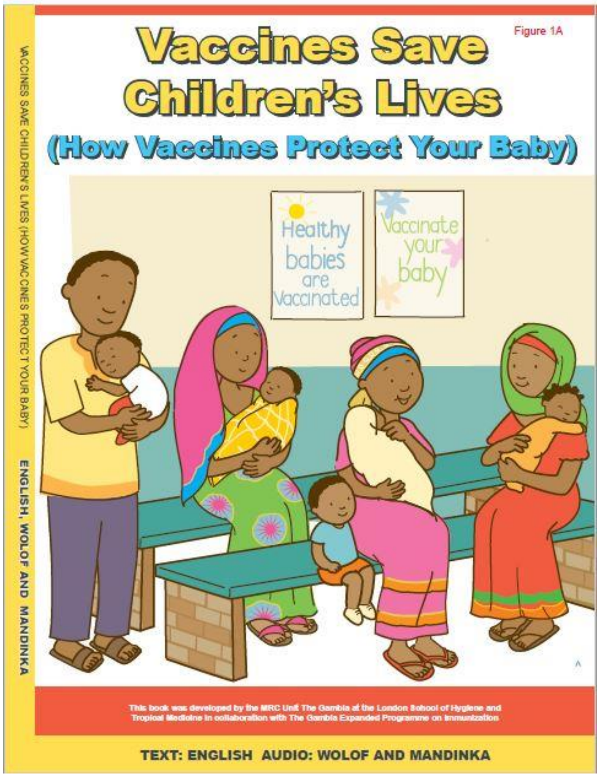
Vaccines Save Children's Lives
(How Vaccines Protect Your Baby)
Figure 1A
Healthy babies are Vaccinated
Vaccinate your baby
VACCINES SAVE CHILDREN'S LIVES (HOW VACCINES PROTECT YOUR BABY)
ENGLISH, WOLOF AND MANDINKA
This book was developed by the MRC Unit The Gambia at the London School of Hygiene and Tropical Medicine in collaboration with The Gambia Expanded Programme on Immunization
TEXT: ENGLISH AUDIO: WOLOF AND MANDINKA

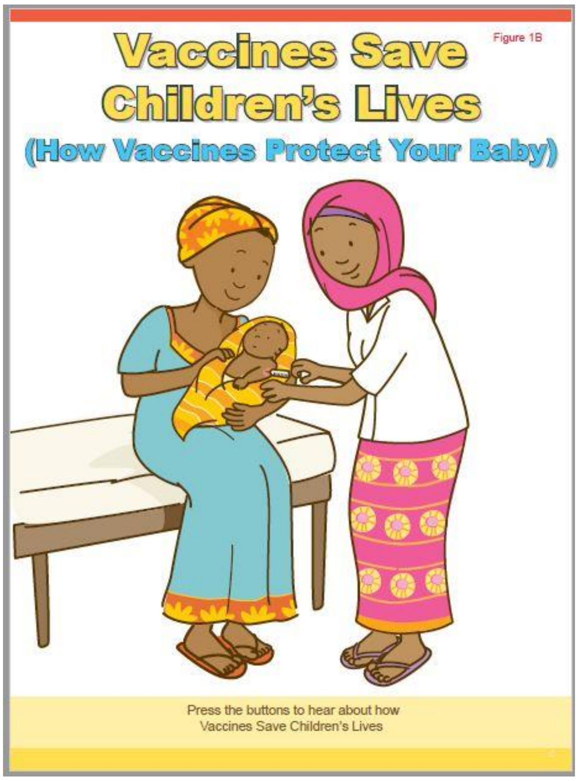
Vaccines Save Children's Lives
(How Vaccines Protect Your Baby)
Figure 1B
Press the buttons to hear about how Vaccines Save Children's Lives

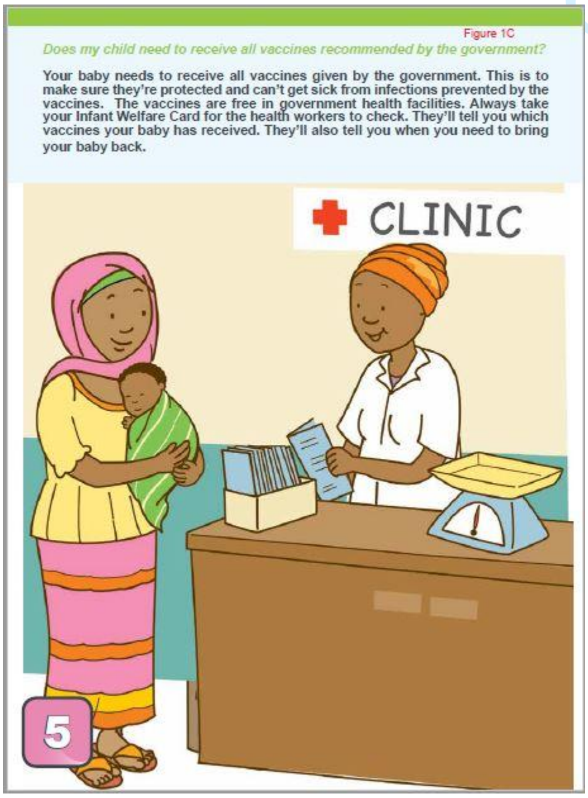
Figure 1C
Does my child need to receive all vaccines recommended by the government?
Your baby needs to receive all vaccines given by the government. This is to make sure they're protected and can't get sick from infections prevented by the vaccines. The vaccines are free in government health facilities. Always take your Infant Welfare Card for the health workers to check. They'll tell you which vaccines your baby has received. They'll also tell you when you need to bring your baby back.
CLINIC
5

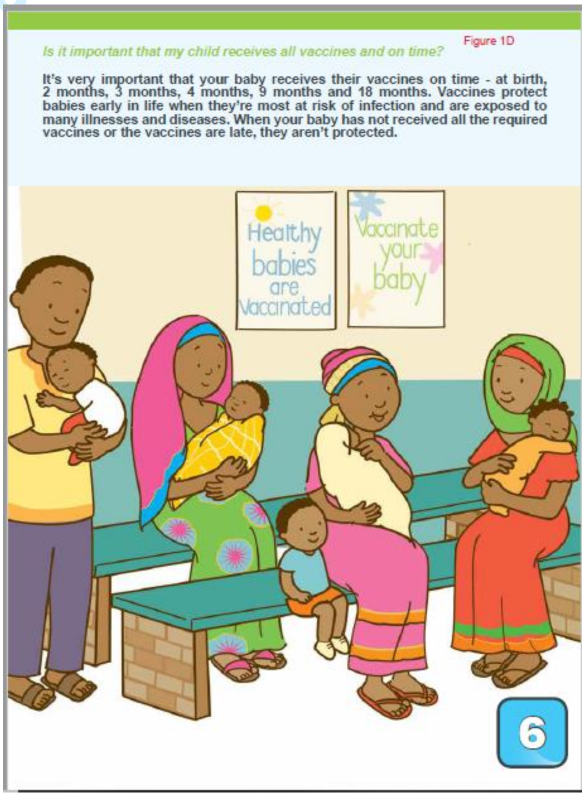
Figure 1D
Is it important that my child receives all vaccines and on time?
It's very important that your baby receives their vaccines on time - at birth, 2 months, 3 months, 4 months, 9 months and 18 months. Vaccines protect babies early in life when they're most at risk of infection and are exposed to many illnesses and diseases. When your baby has not received all the required vaccines or the vaccines are late, they aren't protected.
Healthy babies are Vaccinated
Vaccinate your baby
6

Figure 2

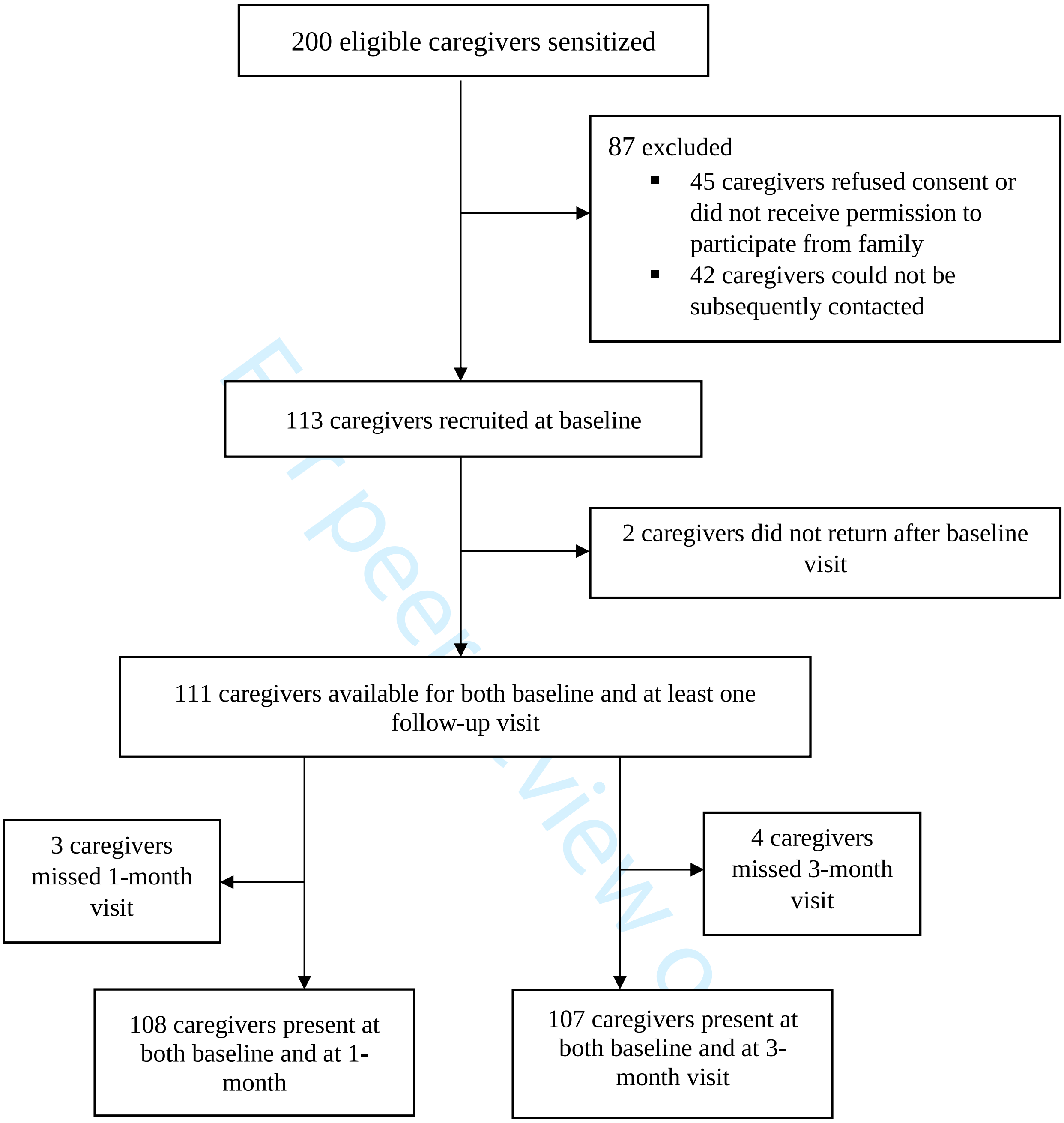
200 eligible caregivers sensitized
87 excluded
45 caregivers refused consent or did not receive permission to participate from family
42 caregivers could not be subsequently contacted
113 caregivers recruited at baseline
2 caregivers did not return after baseline visit
111 caregivers available for both baseline and at least one follow-up visit
3 caregivers missed 1-month visit
4 caregivers missed 3-month visit
108 caregivers present at both baseline and at 1-month
107 caregivers present at both baseline and at 3-month visit

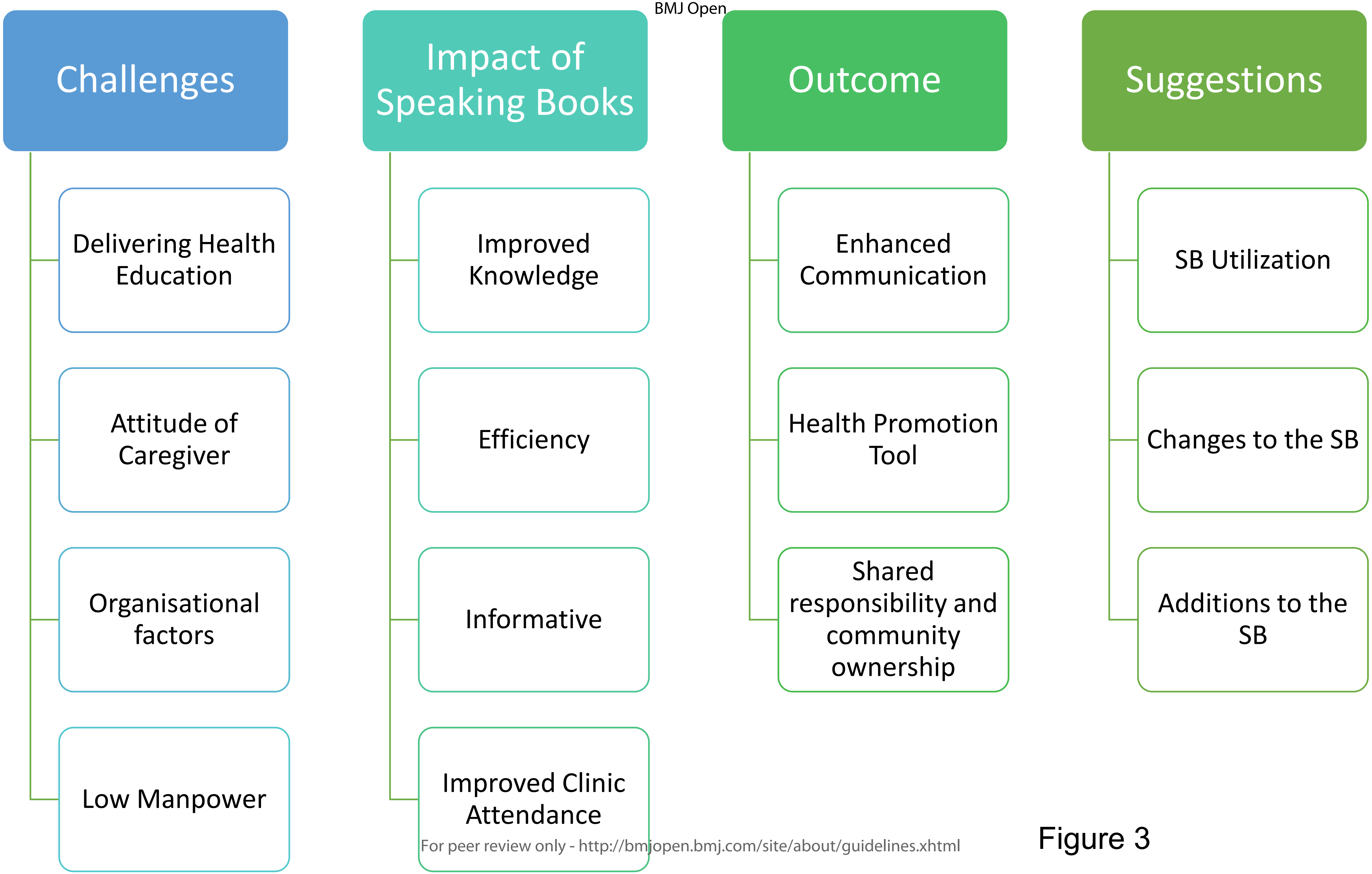
Challenges
Delivering Health Education
Attitude of Caregiver
Organisational factors
Low Manpower
Impact of Speaking Books
Improved Knowledge
Efficiency
Informative
Improved Clinic Attendance
Outcome
Enhanced Communication
Health Promotion Tool
Shared responsibility and community ownership
Suggestions
SB Utilization
Changes to the SB
Additions to the SB

Figure 3

Supplementary material 1_Photos of the Speaking Book

Photos of the speaking book

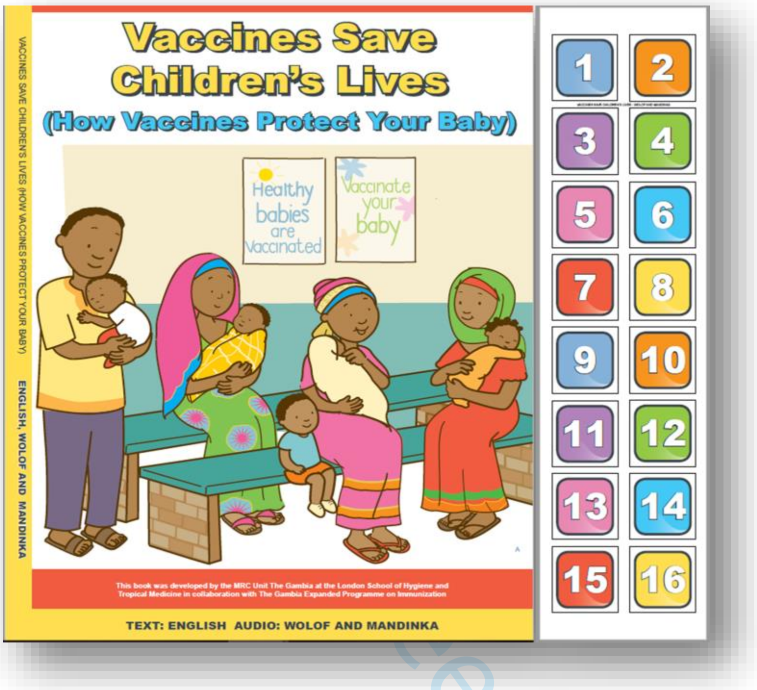

Front view of the SB

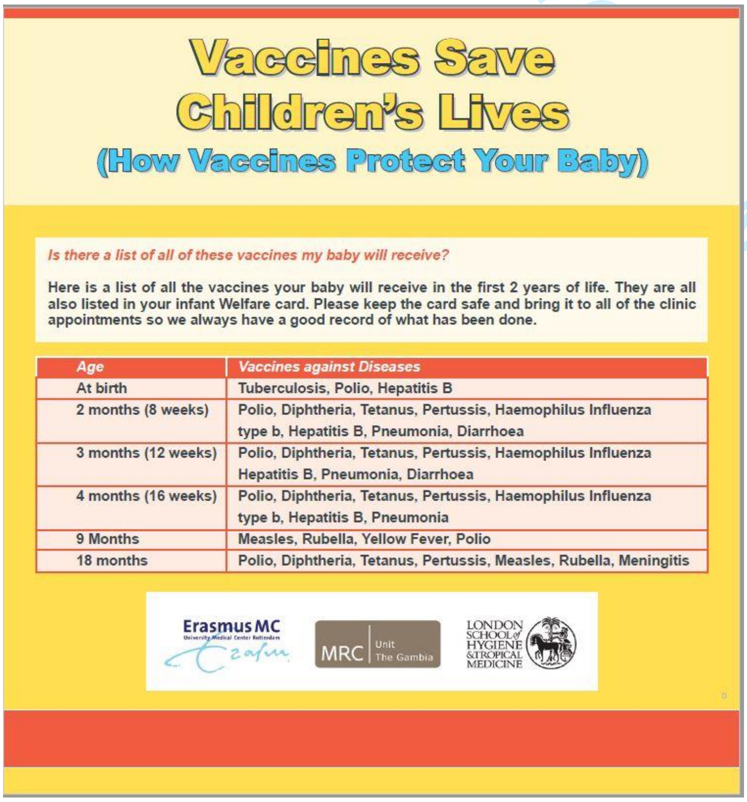

Vaccines Save Children's Lives
(How Vaccines Protect Your Baby)

Is there a list of all of these vaccines my baby will receive?

Here is a list of all the vaccines your baby will receive in the first 2 years of life. They are all also listed in your infant Welfare card. Please keep the card safe and bring it to all of the clinic appointments so we always have a good record of what has been done.

Age	Vaccines against Diseases
At birth	Tuberculosis, Polio, Hepatitis B
2 months (8 weeks)	Polio, Diphtheria, Tetanus, Pertussis, Haemophilus Influenza type b, Hepatitis B, Pneumonia, Diarrhoea
3 months (12 weeks)	Polio, Diphtheria, Tetanus, Pertussis, Haemophilus Influenza Hepatitis B, Pneumonia, Diarrhoea
4 months (16 weeks)	Polio, Diphtheria, Tetanus, Pertussis, Haemophilus Influenza type b, Hepatitis B, Pneumonia
9 Months	Measles, Rubella, Yellow Fever, Polio
18 months	Polio, Diphtheria, Tetanus, Pertussis, Measles, Rubella, Meningitis

Erasmus MC University Medical Center Rotterdam — MRC Unit The Gambia — London School of Hygiene & Tropical Medicine

Back view of the SB

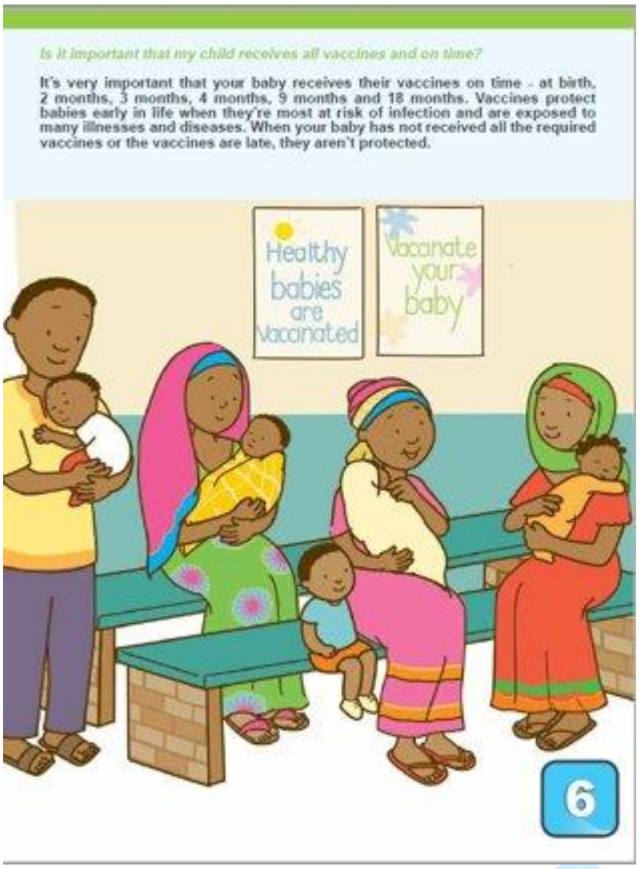

Page 6 of the SB

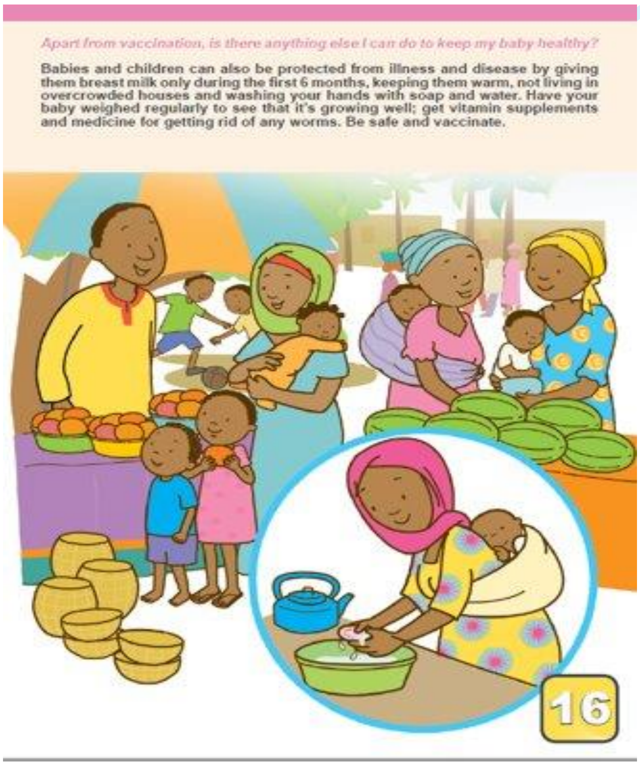

Page 16 of the SB

For peer review only

Study Title: The Vaccines Speaking Book Project

SCC:	1598	Version:	2.1

Sponsor & Funder: MRC & Bull City Learning

For peer review only

Primary Care Givers for the Implementation Study: Section A

Record ID: ______________________________

Facility name: ______________________________

Field worker initials: ______________________________

Participants ID: ______________________________

Date of entry: ______________________________

Primary Care Givers (PCGs)

1. Full Name: ______________________________
2. Contact details (Please collect all available numbers):

   ____

   ____
3. Detailed Address (You can draw behind):

   ____

   ____
4. Age (years): ______________________________
5. Sex:
   - ☐ 1. Female
   - ☐ 2. Male
6. Religion:
   - ☐ Muslim
   - ☐ Christian
   - ☐ Others

6a. If others, specify ______________________________

7. Marital status:
   - ☐ Single
   - ☐ Married
   - ☐ Widowed
   - ☐ Others

7a. If others, specify ______________________________

8. Highest level of Education (Primary care giver):
   - ☐ No education
   - ☐ Primary (Grade 1-6)

SCC:	1598	Version v 1.2

☐ Secondary (Grade 7–9)
☐ Higher Secondary (Grade 10–12)
☐ Tertiary (University education)
☐ Others

8a. If others, specify ____________________

9. Education level of other parent (If applicable): ____________________

10. Employment status:
☐ Employed
☐ Self Employed
☐ Unemployed

11. Employment status of other parent (If applicable):
☐ Employed
☐ Self Employed
☐ Unemployed

12. Occupation: ____________________

13. Occupation of other parent if applicable: ____________________

14. Monthly household income:
☐ I don't know
☐ Below D2500
☐ D2, 500-D5, 000
☐ D5, 000- D10, 000
☐ D10, 000- D20, 000
☐ Above D20, 000

15. Presence of a working radio in the house:
☐ Yes
☐ No

16. Presence of a working television in the house:
☐ Yes
☐ No

17. Family size (number of people eating from one pot): ____________________

18. Mothers age at marriage (years): ____________________

19. Age at first child (years): ____________________

IMMUNISATION

20. Mothers receipt of Tetanus Toxoid during pregnancy:
☐ Yes
☐ No

21. How many times: ____________________

22. Distance from home to Health Facility (Minutes/hours): ____________________

23. Source of information regarding Immunisation:
☐ Healthcare Worker
☐ Information written on the vaccination cards
☐ Family member or friends
☐ Radio/TV
☐ Poster
☐ Others

23a. If others, specify: ____________________

For peer review only - http://bmjopen.bmj.com/site/about/guidelines.xhtml

SCC:	1598	Version v 1.2

24. What do you consider to be the challenge or problem you face attending immunization clinic
____
____

CHILD

25. Age: ____________________
26. Date of Birth: (DD/MON/YEAR) ____________________
27. Sex:
    - ☐ Female
    - ☐ Male
28. Birth order: ____________________
29. Date of starting vaccination: ____________________
30. Evidence of completeness of immunizations if available:
    - ☐ Yes
    - ☐ No
    - ☐ Information not available
31. Place of Delivery of this child:
    - ☐ Home
    - ☐ Health Facility
    - ☐ Others

31a. If others, specify: ____________________

32. Mother's utilization of ANC during the pregnancy:
    - ☐ Yes
    - ☐ No
33. How many visits? ____________________

For peer review only

SCC:	1598	Version v 1.2

Primary Care Givers for the Implementation Study: Section B

Record ID: ______________________________

Facility name: ______________________________

Field worker initials: ______________________________

Participants ID: ______________________________

Date of entry: ______________________________

1. How do vaccines work?
   - ☐ Vaccines help a baby or a pregnant woman's body fight illness and diseases
   - ☐ Vaccines work by making security guards in the body called 'anti-bodies'
   - ☐ I don't know
   - ☐ Others

1a. If others specify: ______________________________

2. Why are vaccines given to pregnant women?
   - ☐ Vaccines ensure mother and child are protected against infectious diseases
   - ☐ If the pregnant woman is vaccinated, the baby is also protected for a little while after birth
   - ☐ I don't know
   - ☐ Others

2a. If others, specify: ______________________________

3. What do you do if your child is not well on the day the vaccines need to be given?
   - ☐ I will take my child to the hospital for the doctor or nurse to decide if my child can be immunised
   - ☐ I will wait at home until my child is well
   - ☐ I don't know
   - ☐ Others

3a. If others, specify: ______________________________

4. Do you think it is proper for your child to be given more than one vaccine at a visit?
   - ☐ Yes, different vaccines prevent different infections/diseases
   - ☐ No, administering more than one vaccine at a time prevents the vaccine from working effectively
   - ☐ I don't know
   - ☐ Others

4a. If others, specify: ______________________________

5. Why do you think your child is sometimes given the same vaccines more than once?
   - ☐ Repeat doses are needed to make the antibodies (body soldiers) stronger
   - ☐ Repeat doses help the baby's body learn to be strong against infections for a long time
   - ☐ I don't know
   - ☐ Others

For peer review only - http://bmjopen.bmj.com/site/about/guidelines.xhtml

SCC:	1598	Version v 1.2

5a. If others, specify: ____
____
____

6. When do I need to consider the adverse event of a vaccine serious?
- ☐ If my baby has high fever for more than a day
- ☐ If my baby is sleeping a lot
- ☐ I don't know
- ☐ Others

6a. If others, specify: ____
____
____

7. What vaccines does your baby receive at each visit? Tick all applicable

7a. At birth
- ☐ Tuberculosis
- ☐ Polio
- ☐ Hepatitis B
- ☐ Diphtheria
- ☐ Whooping Cough
- ☐ Meningitis
- ☐ Pneumonia
- ☐ Diarrhoea
- ☐ Measles
- ☐ Yellow Fever
- ☐ I don't know

7b. At 2 Months
- ☐ Tuberculosis
- ☐ Polio
- ☐ Hepatitis B
- ☐ Diphtheria
- ☐ Whooping Cough
- ☐ Meningitis
- ☐ Pneumonia
- ☐ Diarrhoea
- ☐ Measles
- ☐ Yellow Fever
- ☐ I don't know

7c. At 3 Months
- ☐ Tuberculosis
- ☐ Polio
- ☐ Hepatitis B
- ☐ Diphtheria
- ☐ Whooping Cough
- ☐ Meningitis
- ☐ Pneumonia
- ☐ Diarrhoea
- ☐ Measles
- ☐ Yellow Fever
- ☐ I don't know

7d. At 4 Months
- ☐ Tuberculosis
- ☐ Polio

For peer review only - http://bmjopen.bmj.com/site/about/guidelines.xhtml
For peer review only

SCC:	1598	Version v 1.2

- ☐ Hepatitis B
- ☐ Diphtheria
- ☐ Whooping Cough
- ☐ Meningitis
- ☐ Pneumonia
- ☐ Diarrhoea
- ☐ Measles
- ☐ Yellow Fever
- ☐ I don't know

7e. At 9 Months

- ☐ Tuberculosis
- ☐ Polio
- ☐ Hepatitis B
- ☐ Diphtheria
- ☐ Whooping Cough
- ☐ Meningitis
- ☐ Pneumonia
- ☐ Diarrhoea
- ☐ Measles
- ☐ Yellow Fever
- ☐ I don't know

7f. At 18 Months

- ☐ Tuberculosis
- ☐ Polio
- ☐ Hepatitis B
- ☐ Diphtheria
- ☐ Whooping Cough
- ☐ Meningitis
- ☐ Pneumonia
- ☐ Diarrhoea
- ☐ Measles
- ☐ Yellow Fever
- ☐ I don't know

8. What other ways can you keep your baby healthy apart from vaccination?
   - ☐ Exclusive breast feeding for 6 months
   - ☐ Keeping baby warm
   - ☐ Regular hand washing with soap and water
   - ☐ Regular weighing of the baby at the clinic
   - ☐ Others

8a. If others, specify: ____

____

____

For peer review only

Version 2.1 – 29th January 2019

MRC Unit The Gambia at the
London School of Hygiene
and Tropical Medicine

Study Title: The Vaccines Speaking Book Project

SCC:	1598	Version:	2.1

Sponsor & Funder: MRC & Bull City Learning

For peer review only

Primary Care Givers for the Implementation Study: Section B

Record ID: ______________________________

Facility name: ______________________________

Field worker initials: ______________________________

Participants ID: ______________________________

Date of entry: ______________________________

1. How do vaccines work?
   - ☐ Vaccines help a baby or a pregnant woman's body fight illness and diseases
   - ☐ Vaccines work by making security guards in the body called 'anti-bodies'
   - ☐ I don't know
   - ☐ Others

1a. If others specify: ______________________________

2. Why are vaccines given to pregnant women?
   - ☐ Vaccines ensure mother and child are protected against infectious diseases
   - ☐ If the pregnant woman is vaccinated, the baby is also protected for a little while after birth
   - ☐ I don't know
   - ☐ Others

2a. If others, specify: ______________________________

3. What do you do if your child is not well on the day the vaccines need to be given?
   - ☐ I will take my child to the hospital for the doctor or nurse to decide if my child can be immunised
   - ☐ I will wait at home until my child is well
   - ☐ I don't know
   - ☐ Others

3a. If others, specify: ______________________________

4. Do you think it is proper for your child to be given more than one vaccine at a visit?
   - ☐ Yes, different vaccines prevent different infections/diseases

For peer review only - http://bmjopen.bmj.com/site/about/guidelines.xhtml

SCC:	1598	Version v 1.2

☐ No, administering more than one vaccine at a time prevents the vaccine from working effectively
☐ I don't know
☐ Others

4a. If others, specify: ____
____
____

5. Why do you think your child is sometimes given the same vaccines more than once?
☐ Repeat doses are needed to make the antibodies (body soldiers) stronger
☐ Repeat doses help the baby's body learn to be strong against infections for a long time
☐ I don't know
☐ Others

5a. If others, specify: ____
____
____

6. When do I need to consider the adverse event of a vaccine serious?
☐ If my baby has high fever for more than a day
☐ If my baby is sleeping a lot
☐ I don't know
☐ Others

6a. If others, specify: ____
____
____

7. What vaccines does your baby receive at each visit? Tick all applicable

7a. At birth
☐ Tuberculosis
☐ Polio
☐ Hepatitis B
☐ Diphtheria
☐ Whooping Cough
☐ Meningitis
☐ Pneumonia
☐ Diarrhoea
☐ Measles
☐ Yellow Fever
☐ I don't know

7b. At 2 Months
☐ Tuberculosis
☐ Polio
☐ Hepatitis B
☐ Diphtheria
☐ Whooping Cough
☐ Meningitis
☐ Pneumonia
☐ Diarrhoea
☐ Measles
☐ Yellow Fever
☐ I don't know

7c. At 3 Months
☐ Tuberculosis
☐ Polio
☐ Hepatitis B

For peer review only - http://bmjopen.bmj.com/site/about/guidelines.xhtml

SCC:	1598	Version v 1.2

- ☐ Diphtheria
- ☐ Whooping Cough
- ☐ Meningitis
- ☐ Pneumonia
- ☐ Diarrhoea
- ☐ Measles
- ☐ Yellow Fever
- ☐ I don't know

7d. At 4 Months

- ☐ Tuberculosis
- ☐ Polio
- ☐ Hepatitis B
- ☐ Diphtheria
- ☐ Whooping Cough
- ☐ Meningitis
- ☐ Pneumonia
- ☐ Diarrhoea
- ☐ Measles
- ☐ Yellow Fever
- ☐ I don't know

7e. At 9 Months

- ☐ Tuberculosis
- ☐ Polio
- ☐ Hepatitis B
- ☐ Diphtheria
- ☐ Whooping Cough
- ☐ Meningitis
- ☐ Pneumonia
- ☐ Diarrhoea
- ☐ Measles
- ☐ Yellow Fever
- ☐ I don't know

7f. At 18 Months

- ☐ Tuberculosis
- ☐ Polio
- ☐ Hepatitis B
- ☐ Diphtheria
- ☐ Whooping Cough
- ☐ Meningitis
- ☐ Pneumonia
- ☐ Diarrhoea
- ☐ Measles
- ☐ Yellow Fever
- ☐ I don't know

8. What other ways can you keep your baby healthy apart from vaccination?
   - ☐ Exclusive breast feeding for 6 months
   - ☐ Keeping baby warm
   - ☐ Regular hand washing with soap and water
   - ☐ Regular weighing of the baby at the clinic
   - ☐ Others

For peer review only
For peer review only - http://bmjopen.bmj.com/site/about/guidelines.xhtml

SCC:	1598	Version v 1.2

8a. If others, specify: ____
____
____

For peer review only

SCC:	1598	Version v 1.2

Primary Care Givers for the Implementation Study: Section C

Record ID: ______________________

Facility name: ______________________

Field worker initials: ______________________

Participants ID: ______________________

Date of entry: ______________________

1. Did you find the book easy to use? Yes ☐ No ☐

1a. Can you please give reasons for your response above?

____
____
____
____

2. Did you like the pictures in the book? Yes ☐ No ☐

2a. Can you please give reasons for your response above?

____
____
____
____

3. Could you hear the person talking to you clearly? Yes ☐ No ☐

3a. Can you please give reasons for your response above?

____
____
____
____

4. Did you understand all the information that you were told in the book? Yes ☐ No ☐

4b. Can you please give reasons for your response above?

____
____
____
____

5. Did you find any information in the book useful? Yes ☐ No ☐

For peer review only - http://bmjopen.bmj.com/site/about/guidelines.xhtml

SCC:	1598	Version v 1.2

5a. Can you please give reasons for your response above?

____
____
____
____

5b. If yes, what was the information you found most useful?

____
____
____
____

6. Did you trust the messages in the book? Yes ☐ No ☐

6a. Can you please give reasons for your response above?

____
____
____
____

7. Did you learn new information from the book? Yes ☐ No ☐

7a. Can you please give reasons for your response above?

____
____
____
____

8. How many times did you go through the whole book? 1 ☐ 2-5 ☐ 6-9 ☐ 10 or more times ☐

9. Can you clearly explain the information in the book to your family and friends? Yes ☐ No ☐

9a. Can you please give reasons for your response above?

____
____
____
____

10. Do you think that the information in the book gives you all the information needed to make a decision to immunize your child? Yes ☐ No ☐

For peer review only - http://bmjopen.bmj.com/site/about/guidelines.xhtml

SCC:	1598	Version v 1.2

10b. Can you please give reasons for your response above?

____
____
____
____

11. Were there any problems you had when you were using the book? Yes ☐ No ☐

11b. If Yes, what problem(s) did you experience?

____
____
____
____

12. Do you think the book should include any other information or have any changes? Yes ☐ No ☐

12b. If Yes, what changes would you suggest?

____
____
____
____

13. Did you show the book to anyone in your Family? Yes ☐ No ☐

13a. If yes, how many people ______________

13b. Did you show the book to anyone in your Community? Yes ☐ No ☐

13c. If yes, how many people ______________

13d. Did you show the book to anyone in your Mosque/Church? Yes ☐ No ☐

13e. If yes, how many people ______________

13f. Did you show the book to anyone at work? Yes ☐ No ☐

13g. If yes, how many people ______________

13h. Did you show the book to anyone in your clinic or mosque? Yes ☐ No ☐

13i. If yes, how many people ______________

13j. Can you please specify any other place you shared the book? ______________

14. Do you think members of your family, mosque/church, and community will understand the information in the book if they were given this book to listen to? Yes ☐ No ☐ I don't know ☐

For peer review only - http://bmjopen.bmj.com/site/about/guidelines.xhtml

SCC:	1598	Version v 1.2

14a. Can you please give reasons for your response above?

____
____
____
____

15. At what time do you think that the books should be given to a parent coming to get their child immunized?
    - At the time of first visit to the clinic only ☐
    - At first few visits to the clinic ☐
    - While speaking to the Health care worker ☐
    - Before speaking to the Healthcare Worker ☐
    - After speaking to the Healthcare worker ☐
    - Copy of the book is useful to stay with the parent at home ☐

16. Do you know where the on/off switch is in the book? Yes ☐ No ☐

17. Would you be able to change the battery? Yes ☐ No ☐

18. If you need to change the battery, do you have a screwdriver with which to do so? Yes ☐ No ☐

For peer review only

Version	1.2	Date	8th January 2019

Questionnaire for Health Care Workers (HCPs)/ Heads of the Health Centres where the SB invention was implemented

Record ID: ________________________________

Facility name: ________________________________

Field worker initials: ________________________________

Participants ID: ________________________________

Date of entry: ________________________________

1. Age: ____________________
2. Gender: Female ☐ Male ☐
3. Time of working in the current position (years): ____________________
4. Do HCWs have enough time to make sure that Primary Care Givers (PCG) completely understand all the information explained to them about immunization? Yes ☐ No ☐

4a. If No, why not?

____
____
____
____

5. Did the book help you in addressing the Primary Care Givers (PCGs) questions and concerns? Yes ☐ No ☐

5a. Can you please give reasons for your response above?

____
____
____
____

6. Did the book make more efficient use of your time and energy? Yes ☐ No ☐

6a. Can you please give reasons for your response above?

____
____
____
____

For peer review only - http://bmjopen.bmj.com/site/about/guidelines.xhtml

SCC:	1598	Version v 2.1

7. Did the book increase knowledge of the PCG? Yes ☐ No ☐ I don't know ☐

7a. Can you please give a reason for your answer above?

____
____
____
____

8. Did the book increase communication between the PCG and you? Yes ☐ No ☐

8a. Can you please give a reason for your answer above?

____
____
____
____

9. Was there any other impact of the speaking book on the Primary Care Givers? Yes ☐ No ☐

If yes, what was the impact?

____
____
____

9a. Did the Primary Care Givers (PCGs) ask more questions? Yes ☐ No ☐

9b. Did you observe timelier Immunisation done of their child by the PCG? ` Yes ☐ No ☐ I don't know ☐

9c. Other observations? (Please mention)

____
____
____

10. When do you think the SB should be given to the PCGs?
- At the time of first visit to the clinic only ☐
- At first few visits to the clinic ☐
- While speaking to the Health care worker ☐
- Before speaking to the Healthcare Worker ☐
- After speaking to the Healthcare worker ☐
- Copy of the book is useful to stay with the parent at home ☐

11. Do you think the book should include any other information or have any changes? Yes ☐ No ☐

11a. What information would you suggest to be added?

____
____
____
____

For peer review only - http://bmjopen.bmj.com/site/about/guidelines.xhtml

Supplementary material 5_Themes, sub-themes and quotes

Themes	Subthemes	Supporting quotes
General Challenges at the Clinics, independent of the SBs	Delivering Health Education	"… we normally conduct pre-clinic health talks… we discuss vaccines, and sometimes we choose other topics…. about their health and that of their babies" **(Male, Rural clinic, 18 months in current position)**<br><br>"When we have a large crowd, we also engage them in interpersonal communication while administering the vaccines, we tell them about the vaccines been administered and the possible side effects of the vaccine"**(Male, Rural clinic, 6 months in current position)**
	Attitude of caregiver	"When women come to the clinic, they are always in hurry to go back home to cook or going back to the garden, so most times they are in haste to go home" **(Male, Urban clinic, 5 years in current position)**<br><br>"What we do sometimes is to hold their cards and start weighing, and then when most of the women are around, we start giving the health education" **(Male, Rural clinic, 8 months in current position)**
	Organizational factors	"…most of them [caregivers] come around the same time and the health care worker might not have time to carefully explain in detail to the women … most times the environment is even very noisy" **(Male, Rural clinic, 3years in current position)**<br><br>"…. some of the women will be standing and distracted by the cry of their children and may not even have the patience to listen to what we are saying to them" Adding that: "Interpersonal health education is usually better for us with the crowd (High numbers of caregivers)" **(Female, Rural clinic, 3 years in current position)**
	Low manpower	"… when we started the implementation of the speaking books, I was the only Public Health Officer here without any form of assistance or back up." **(Male, Urban clinic, 5 years in current position)**<br><br>"Sometimes I will be the only one immunizing, weighing and screening so sometimes it's difficult to use the speaking books" **(Male, Urban clinic, 5 years in current position)**
SB Impact	Improved Knowledge	"Most times after listening to the books, we ask them questions on immunisation and they are able to give positive feedback" **(Male, Urban clinic, 18 months in current position)**<br><br>"… I cannot assure you on their [all caregivers attending the immunisation clinic] increase in knowledge compared to women who have personal copies [recruited participants] with them at home." **(Male, Rural clinic, 8months in current position)**
	Efficiency	"…for me it saves time because I just hand over the book to them [Caregiver] and then they listen to everything …. while I concentrate on the work and it makes work faster." **(Female, Rural clinic, 3 years in current position)**

For peer review only

		"… the issue is we have just one copy in the facility which …. is not enough…. they all [Caregiver] have to keep waiting for each other to listen to the books… if we have at least 5 copies it would be better than everyone sharing one copy." **(Male, Urban clinic, 18 months in current position)**
	Informative	"In the past they give so many complains about adverse effects following vaccination……. even blame public health officers that administer the vaccines, they have a better understanding of the side effects after receiving the vaccines and appreciate the information gotten from the book" **(Female, Rural clinic, 3 years in current position)**<br><br>"It has addressed all the details and relevant information that will make mothers want to bring their children for immunization" **(Female, Rural clinic, 3 years in current position)**
	Improved Clinic Attendance	"Mothers are beginning to bring their babies to the clinic unlike before when they will just come to the clinic anytime they like" **(Female, Urban clinic, 10 years in current position)**<br><br>"In the past we used to have low coverages but with the introduction of the speaking books are now seeing more mothers coming" **(Male, Urban clinic, 5 years in current position)**
Outcome of use of the SBs	Enhanced Communication	"The book has made it easier for us to explain information on vaccines to the mothers…" **(Female, Urban clinic, 10 years in current position)**<br><br>"… most of the women don't understand English but with the use of the local languages they understand the information easily……. it has been easier for us to pass across the information because English is not our language, so it is easier for them to understand and easier for us to explain to them" **(Male, Urban clinic, 3 years in current position)**
	Health Promotion Tool	"… we are trying, by recording the audio on our phones and sharing with other health staff because not all health officers have access to the information in this book…. we learnt a lot from it." **(Male, Urban clinic, 3 years in current position)**<br><br>"…… because the book is not loud for all the mothers to hear, I recorded it on my phone, used a Bluetooth device to connect to a speaker for all the mothers to hear" **(Male, Urban clinic, 2 years in current position)**
	Shared responsibility and community ownership	"They share during lunch or when they sit together, they play the book" **(Male, Rural clinic, 8months in current position)**<br><br>"During the immunisation clinic days most of the women who have copies of the speaking books come along with the books and they normally take over the health talks with the use of their speaking books, sometimes I don't even play my own"**(Male, Urban clinic, 5 years in current position)**
Further Suggestions	SB utilization	"I think is better they are given the books at their homes …. in the clinic most times they are in hurry to go home …. at home they will take out time to listen to the book and gain more information compared to listening to the books in the clinic" **(Male, Urban clinic, 5 years in current position)**

For peer review only

		"…. I believe the women will make better use of the speaking books if they are used in the facilities than in their homes ... at the health facility the health workers will be around to guide them, this will ensure a better use of the book…." **(Male, Urban clinic, 18 months in current position)**
	Changes to the SB	"The only problem the speaking book has is that it cannot be used with a large audience because the volume is very low" **(Female, Rural clinic, 3years in current position)**<br><br>"….. it will be good to have a book that is very loud so we don't have to talk, we will just place it at the front of the Clinic, press the buttons and the book will start playing for the women to listen to it" **(Male, Rural clinic, 6 months in current position)**
	Additions to the SB	"…other languages need to be added…. because caregivers who do not understand any of the languages used in the book did not benefit from the information in the book…." **(Male, Urban clinic, 4 years in current position)**<br><br>"…. we can also consider adding information on nutrition and importance of antenatal care by pregnant women" **(Female, Urban clinic, 10 years in current position**

For peer review only
